# Genome-wide analyses reveal a potential role for the *MAPT*, *MOBP*, and *APOE* loci in sporadic frontotemporal dementia

**DOI:** 10.1016/j.ajhg.2024.05.017

**Published:** 2024-06-17

**Authors:** Claudia Manzoni, Demis A. Kia, Raffaele Ferrari, Ganna Leonenko, Beatrice Costa, Valentina Saba, Edwin Jabbari, Manuela MX. Tan, Diego Albani, Victoria Alvarez, Ignacio Alvarez, Ole A. Andreassen, Antonella Angiolillo, Andrea Arighi, Matt Baker, Luisa Benussi, Valentina Bessi, Giuliano Binetti, Daniel J. Blackburn, Merce Boada, Bradley F. Boeve, Sergi Borrego-Ecija, Barbara Borroni, Geir Bråthen, William S. Brooks, Amalia C. Bruni, Paola Caroppo, Sara Bandres-Ciga, Jordi Clarimon, Rosanna Colao, Carlos Cruchaga, Adrian Danek, Sterre CM. de Boer, Itziar de Rojas, Alfonso di Costanzo, Dennis W. Dickson, Janine Diehl-Schmid, Carol Dobson-Stone, Oriol Dols-Icardo, Aldo Donizetti, Elise Dopper, Elisabetta Durante, Camilla Ferrari, Gianluigi Forloni, Francesca Frangipane, Laura Fratiglioni, Milica G. Kramberger, Daniela Galimberti, Maurizio Gallucci, Pablo García-González, Roberta Ghidoni, Giorgio Giaccone, Caroline Graff, Neill R. Graff-Radford, Jordan Grafman, Glenda M. Halliday, Dena G. Hernandez, Lena E. Hjermind, John R. Hodges, Guy Holloway, Edward D. Huey, Ignacio Illán-Gala, Keith A. Josephs, David S. Knopman, Mark Kristiansen, John B. Kwok, Isabelle Leber, Hampton L. Leonard, Ilenia Libri, Alberto Lleo, Ian R. Mackenzie, Gaganjit K. Madhan, Raffaele Maletta, Marta Marquié, Ales Maver, Manuel Menendez-Gonzalez, Graziella Milan, Bruce L. Miller, Christopher M. Morris, Huw R. Morris, Benedetta Nacmias, Judith Newton, Jørgen E. Nielsen, Christer Nilsson, Valeria Novelli, Alessandro Padovani, Suvankar Pal, Florence Pasquier, Pau Pastor, Robert Perneczky, Borut Peterlin, Ronald C. Petersen, Olivier Piguet, Yolande AL. Pijnenburg, Annibale A. Puca, Rosa Rademakers, Innocenzo Rainero, Lianne M. Reus, Anna MT. Richardson, Matthias Riemenschneider, Ekaterina Rogaeva, Boris Rogelj, Sara Rollinson, Howard Rosen, Giacomina Rossi, James B. Rowe, Elisa Rubino, Agustin Ruiz, Erika Salvi, Raquel Sanchez-Valle, Sigrid Botne Sando, Alexander F. Santillo, Jennifer A. Saxon, Johannes CM. Schlachetzki, Sonja W. Scholz, Harro Seelaar, William W. Seeley, Maria Serpente, Sandro Sorbi, Sabrina Sordon, Peter St George-Hyslop, Jennifer C. Thompson, Christine Van Broeckhoven, Vivianna M. Van Deerlin, Sven J. Van der Lee, John Van Swieten, Fabrizio Tagliavini, Julie van der Zee, Arianna Veronesi, Emilia Vitale, Maria Landqvist Waldo, Jennifer S. Yokoyama, Mike A. Nalls, Parastoo Momeni, Andrew B. Singleton, John Hardy, Valentina Escott-Price

**Affiliations:** 1 UCL School of Pharmacy, London, UK; 2 Department of Clinical and Movement Neurosciences, UCL Queen Square Institute of Neurology, London, UK; 3 Division of Psychological Medicine and Clinical Neurosciences, UK Dementia Research Institute, School of Medicine, Cardiff University, Cardiff, UK; 4 Medical and Genomic Statistics Unit, Department of Brain and Behavioral Sciences, University of Pavia, Pavia, Italy; 5 Department of Neurology, Oslo University Hospital, Oslo, Norway; 6 Istituto di Ricerche Farmacologiche Mario Negri IRCCS, Milano, Italy; 7 Hospital Universitario Central de Asturias, Oviedo, Spain; 8 Instituto de Investigación Sanitaria del Principado de Asturias, Oviedo, Spain; 9 Memory Disorders Unit, Department of Neurology, Hospital Universitari Mutua de Terrassa, Terrassa, Barcelona, Spain; 10 Fundació Docència i Recerca MútuaTerrassa, Terrassa, Barcelona, Spain; 11 NORMENT Centre, Institute of Clinical Medicine, University of Oslo, Oslo, Norway; 12 Division of Mental Health and Addiction, Oslo University Hospital, Oslo, Norway; 13 Centre for Research and Training in Medicine of Aging, Department of Medicine and Health Science “V. Tiberio,” University of Molise, Campobasso, Italy; 14 Fondazione IRCCS Ca' Granda, Ospedale Maggiore Policlinico, Milan, Italy; 15 Department of Neuroscience, Mayo Clinic Jacksonville, Jacksonville, FL, USA; 16 Molecular Markers Laboratory, IRCCS Istituto Centro San Giovanni di Dio Fatebenefratelli, Brescia, Italy; 17 Department of Neuroscience, Psychology, Drug Research and Child Health, University of Florence, Florence, Italy; 18 MAC-Memory Clinic and Molecular Markers Laboratory, IRCCS Istituto Centro San Giovanni di Dio Fatebenefratelli, Brescia, Italy; 19 University of Sheffield, Sheffield, UK; 20 Research Center and Memory Clinic. Ace Alzheimer Center Barcelona – Universitat Internacional de Catalunya, Barcelona, Spain; 21 CIBERNED, Network Center for Biomedical Research in Neurodegenerative Diseases, National Institute of Health Carlos III, Madrid, Spain; 22 Department of Neurology, Mayo Clinic Rochester, Rochester, MN, USA; 23 Alzheimer’s Disease and Other Cognitive Disorders Unit, Service of Neurology. Hospital Clínic de Barcelona, Fundació Clínic Barcelona-IDIBAPS, Barcelona, Spain; 24 Department of Clinical and Experimental Sciences, University of Brescia, Brescia, Italy; 25 Department of Neurology and Clinical Neurophysiology, University Hospital of Trondheim, Trondheim, Norway; 26 Department of Neuromedicine and Movement Science, Faculty of Medicine and Health Sciences, Norwegian University of Science and Technology, Trondheim, Norway; 27 Neuroscience Research Australia, and Randwick Clinical Campus, UNSW Medicine and Health, University of New South Wales, Sydney, Australia; 28 Regional Neurogenetic Centre, ASPCZ, Lamezia Terme, Italy; 29 Unit of Neurology (V) and Neuropathology, Fondazione IRCCS Istituto Neurologico Carlo Besta, Milano, Italy; 30 Center for Alzheimer’s and Related Dementias, National Institute on Aging and National Institute of Neurological Disorders and Stroke, National Institutes of Health, Bethesda, MD, USA; 31 Memory Unit, Neurology Department and Sant Pau Biomedical Research Institute, Hospital de la Santa Creu i Sant Pau, Universitat Autònoma de Barcelona, Barcelona, Spain; 32 Department of Psychiatry, Washington University School of Medicine, St. Louis, MO, USA; 33 NeuroGenomics and Informatics Center, Washington University School of Medicine, St. Louis, MO, USA; 34 Neurologische Klinik, LMU Klinikum, Munich, Germany; 35 Alzheimer Center Amsterdam, Neurology, Vrije Universiteit Amsterdam, Amsterdam UMC location VUmc, Amsterdam, the Netherlands; 36 Amsterdam Neuroscience, Neurodegeneration, Vrije Universiteit Amsterdam, Amsterdam UMC location VUmc, Amsterdam, the Netherlands; 37 Brain and Mind Centre, University of Sydney, Sydney, NSW, Australia; 38 Department of Psychiatry and Psychotherapy, Klinikum rechts der Isar, Technical University of Munich, School of Medicine, Munich, Germany; 39 kbo-Inn-Salzach-Klinikum, Wasserburg, Germany; 40 School of Medical Sciences, University of Sydney, Sydney, NSW, Australia; 41 Department of Biology, University of Naples Federico II, Naples, Italy; 42 Department of Neurology & Alzheimer Center, Erasmus University Medical Center, Rotterdam, the Netherlands; 43 Immunohematology and Transfusional Medicine Service, Local Health Authority n.2 Marca Trevigiana, Treviso, Italy; 44 Karolinska Institutet, Department NVS, KI-Alzheimer Disease Research Center, Stockholm, Sweden; 45 Theme Inflammation and Aging, Karolinska Universtiy Hospital, Stockholm, Sweden; 46 Department of Neurology, University Medical Center, Medical faculty, Ljubljana University of Ljubljana, Ljubljana, Slovenia; 47 Karolinska Institutet, Department of Neurobiology, Care Sciences and Society (NVS), Division of Clinical Geriatrics, Huddinge, Sweden; 48 Department of Biomedical, Surgical and Dental Sciences, University of Milan, Milan, Italy; 49 Cognitive Impairment Center, Local Health Authority n.2 Marca Trevigiana, Treviso, Italy; 50 Unit for hereditary dementia, Karolinska Universtiy Hospital-Solna, Stockholm, Sweden; 51 Department of Neurology, Mayo Clinic Jacksonville, Jacksonville, FL, USA; 52 Shirley Ryan AbilityLab, Chicago, IL, USA; 53 Laboratory of Neurogenetics, National Institute on Aging, National Institutes of Health, Bethesda, MD, USA; 54 Neurogenetics Clinic & Research Lab, Danish Dementia Research Centre, Copenhagen University Hospital, Copenhagen, Denmark; 55 Anne Rowling Regenerative Neurology Clinic, University of Edinburgh, Edinburgh, UK; 56 Bio Med Psychiatry & Human Behavior, Brown University, Providence, RI, USA; 57 UCL Genomics, London, UK; 58 UCL Great Ormond Street Institute of Child Health, London, UK; 59 Zayed Centre for Research into Rare Disease in Children, London, UK; 60 Sorbonne Université, INSERM U1127, CNRS 7225, Institut du Cerveau - ICM, Paris, France; 61 AP-HP Sorbonne Université, Pitié-Salpêtrière Hospital, Department of Neurology, Institute of Memory and Alzheimer’s Disease, Paris, France; 62 Data Tecnica International LLC, Washington, DC, USA; 63 DZNE Tübingen, Tübingen, Germany; 64 Department of Pathology, University of British Columbia, Vancouver, Canada; 65 Department of Pathology, Vancouver Coastal Health, Vancouver, Canada; 66 Clinical institute of Genomic Medicine, University Medical Center Ljubljana, Ljubljana, Slovenija; 67 Universidad de Oviedo, Medicine Department, Oviedo, Spain; 68 Geriatric Center "Frullone" ASL NA1, Naples, Italy; 69 Memory and Aging Center, Department of Neurology, Weill Institute for Neurosciences, University of California, San Francisco, San Francisco, CA, USA; 70 Global Brain Health Institute, University of California, San Francisco, San Francisco, CA, USA; 71 Trinity College Dublin, Dublin, Ireland; 72 Newcastle Brain Tissue Resource, Newcastle University, Edwardson Building, Nuns Moor Road, Newcastle upon Tyne, UK; 73 IRCCS Fondazione Don Carlo Gnocchi, Florence, Italy; 74 Department of Clinical Sciences, Neurology, Lund University, Lund/Malmö, Sweden; 75 Centro Cardiologico Monzino IRCCS, Milan, Italy; 76 University of Lille, Lille, France; 77 CHU Lille, Lille, France; 78 Inserm, Labex DISTALZ, LiCEND, Lille, France; 79 Unit of Neurodegenerative Diseases, Department of Neurology, University Hospital Germans Trias i Pujol, Badalona, Barcelona, Spain; 80 The Germans Trias i Pujol Research Institute (IGTP) Badalona, Barcelona, Spain; 81 Department of Psychiatry and Psychotherapy, LMU Hospital, Ludwig-Maximilians-Universität Munich, Munich, Germany; 82 German Center for Neurodegenerative Diseases (DZNE) Munich, Munich, Germany; 83 Munich Cluster for Systems Neurology (SyNergy), Munich, Germany; 84 Ageing Epidemiology (AGE) Research Unit, School of Public Health, Imperial College London, London, UK; 85 Sheffield Institute for Translational Neuroscience (SITraN), University of Sheffield, Sheffield, UK; 86 School of Psychology, University of Sydney, Sydney, NSW, Australia; 87 Department of Medicine, Surgery and Dentistry "Scuola Medica Salernitana," University of Salerno, Fisciano, Italy; 88 Cardiovascular Research Unit, IRCCS MultiMedica, Milan, Italy; 89 VIB Center for Molecular Neurology, VIB, Antwerp, Belgium; 90 Department of Biomedical Sciences, University of Antwerp, Antwerp, Belgium; 91 Department of Neuroscience, "Rita Levi Montalcini," University of Torino, Torino, Italy; 92 Center for Alzheimer’s Disease and Related Dementias, Department of Neuroscience and Mental Health, A.O.UCittà della Salute e della Scienza di Torino, Torino, Italy; 93 Center for Neurobehavioral Genetics, Semel Institute for Neuroscience and Human Behavior, University of California, Los Angeles, Los Angeles, CA, USA; 94 Manchester Centre for Clinical Neurosciences, Northern Care Alliance NHS Trust, Manchester Academic Health Sciences Unit, University of Manchester, Manchester, UK; 95 Department of Psychiatry, Saarland University, Homburg, Germany; 96 Tanz Centre for Research in Neurodegenerative Diseases and Department of Medicine, University of Toronto, Toronto, ON, Canada; 97 Department of Biotechnology, Jožef Stefan Institute, Ljubljana, Slovenia; 98 Faculty of Chemistry and Chemical Technology, University of Ljubljana, Ljubljana, Slovenia; 99 Division of Neuroscience and Experimental Psychology, School of Biological Sciences, University of Manchester, Manchester, UK; 100 Department of Neurology, University of California, San Francisco, San Francisco, CA, USA; 101 University of Cambridge Department of Clinical Neurosciences and Cambridge University Hospitals NHS Trust, Cambridge, UK; 102 Unit of Neuroalgologia (III), Fondazione IRCCS Istituto Neurologico Carlo Besta, Milano, Italy; 103 Data science center, Fondazione IRCCS Istituto Neurologico Carlo Besta, Milan, Italy; 104 Department of Clinical Sciences, Clinical Memory Research Unit, Faculty of Medicine, Lund University, Lund/Malmö, Sweden; 105 Department of Cellular and Molecular Medicine, University of California, San Diego, La Jolla, CA, USA; 106 Neurodegenerative Diseases Research Unit, National Institute of Neurological Disorders and Stroke, Bethesda, MD, USA; 107 Department of Neurology, Johns Hopkins University Medical Center, Baltimore, MD, USA; 108 Department of Neurology, Columbia University, New York, NY, USA; 109 Neurodegenerative Brain Diseases, VIB Center for Molecular Neurology, VIB, Antwerp, Belgium; 110 Perelman School of Medicine at the University of Pennsylvania, Department of Pathology and Laboratory Medicine, Center for Neurodegenerative Disease Research, Philadelphia, PA, USA; 111 Section Genomics of Neurodegenerative Diseases and Aging, Department of Clinical Genetics, Vrije Universiteit Amsterdam, Amsterdam UMC, Amsterdam, the Netherlands; 112 Institute of Biochemistry and Cell Biology, National Research Council (CNR), Naples, Italy; 113 School of Integrative Science and Technology Department of Biology Kean University, Union, NJ, USA; 114 Clinical Sciences Helsingborg, Department of Clinical Sciences, Lund University, Lund, Sweden; 115 Department of Radiology and Biomedical Imaging, University of California, San Francisco, San Francisco, CA, USA; 116 Rona Holdings, Cupertino, CA, USA; 117 UK Dementia Research Institute at UCL and Department of Neurodegenerative Disease, UCL Queen Square Institute of Neurology, London, UK; 118 Reta Lila Weston Institute, UCL Queen Square Institute of Neurology, London, UK; 119 NIHR University College London Hospitals Biomedical Research Centre, London, UK; 120 Institute for Advanced Study, The Hong Kong University of Science and Technology, Hong Kong SAR, China

## Abstract

Frontotemporal dementia (FTD) is the second most common cause of early-onset dementia after Alzheimer disease (AD). Efforts in the field mainly focus on familial forms of disease (fFTDs), while studies of the genetic etiology of sporadic FTD (sFTD) have been less common. In the current work, we analyzed 4,685 sFTD cases and 15,308 controls looking for common genetic determinants for sFTD. We found a cluster of variants at the *MAPT* (rs199443; *p* = 2.5 × 10^−12^, OR = 1.27) and *APOE* (rs6857; *p* = 1.31 × 10^−12^, OR = 1.27) loci and a candidate locus on chromosome 3 (rs1009966; *p* = 2.41 × 10^−8^, OR = 1.16) in the intergenic region between *RPSA* and *MOBP*, contributing to increased risk for sFTD through effects on expression and/or splicing in brain cortex of functionally relevant in-*cis* genes at the *MAPT* and *RPSA*-*MOBP* loci. The association with the *MAPT* (H1c clade) and *RPSA*-*MOBP* loci may suggest common genetic pleiotropy across FTD and progressive supranuclear palsy (PSP) (*MAPT* and *RPSA*-*MOBP* loci) and across FTD, AD, Parkinson disease (PD), and cortico-basal degeneration (CBD) (*MAPT* locus). Our data also suggest population specificity of the risk signals, with *MAPT* and *APOE* loci associations mainly driven by Central/Nordic and Mediterranean Europeans, respectively. This study lays the foundations for future work aimed at further characterizing population-specific features of potential FTD-discriminant *APOE* haplotype(s) and the functional involvement and contribution of the *MAPT H1c* haplotype and *RPSA*-*MOBP* loci to pathogenesis of sporadic forms of FTD in brain cortex.

## Teaser

We identified associations at the *MAPT*, *APOE*, and *MOBP* loci with FTD, suggesting potential common genetic denominators across multiple neurological conditions (i.e., FTD, PSP, ALS, AD, PD, and CBD). These findings will aid in the further characterization of the common and disease-specific pathogenesis of, and interventions for, these disorders.

## Introduction

The set of neuropathologies known as frontotemporal lobar degeneration (FTLD; MIM: 607485) make up the second most common cause of early-onset dementia after Alzheimer disease (AD; MIM: 104310).^1^^,^^2^ The most common presentation is frontotemporal dementia (FTD; MIM: 600274), which itself comprises heterogeneous clinical, pathological, and genetic features.

Clinically, the two major syndromes are the behavioral (bvFTD) and the language variants (primary progressive aphasias [PPAs]).^3^^,^^4^ The latter are further subdivided into semantic dementia (SD or semantic variant PPA [svPPA]) and progressive non-fluent aphasia (PNFA or nonfluent/agrammatic variant PPA [nfvPPA]).^3^^,^^5^ FTD can also overlap with motor-neuron disease (FTD-MND)^6^ and share clinical features with progressive supranuclear palsy (PSP; MIM: 601104) and the corticobasal syndrome (CBS; no MIM).^7^ Pathologically, Tau and TDP-43 are the most frequent protein aggregates that define the pathological subtypes of FTLD-tau and FTLD-TDP (≤45% and ≤50% of all affected individuals, respectively).^8^ Genetically, familial FTD (fFTD; ∼30% of all FTD-affected individuals) is predominantly linked to mutations in *MAPT* (MIM: 157140), *GRN* (MIM: 138945), and *C9orf72* (MIM: 614260)^9^^,^^10^; of note, *GFRA2* (MIM: 601956) and *TMEM106B* (MIM: 613413) (previously reported in a cohort with TDP-43 pathology^11^) were found to be associated with increased risk in a *GRN* mutation FTD cohort.^12^ Sporadic FTD (sFTD; ∼70% of all affected individuals) has been associated with genetic risk markers at the *TMEM106B*, *DPP6* (MIM: 126141), *UNC13A* (MIM: 609894) and *HLA-DQA2* (MIM: 613503) loci in cohorts with TDP-43 pathology^11^^,^^13^ and *HLA-DRs* (MIM: 142860) reported in an sFTD cohort encompassing all clinical subtypes.^14^

Efforts in the field have mainly focused on fFTDs, while fewer studies sought to determine the genetic etiology of apparently sFTDs (discussed in Eichler et al., 2010 and Ferrari et al., 2019^15^^,^^16^). In the current work, we analyzed 4,685 sFTD cases and 15,308 controls to identify common genetic determinants contributing to increased risk of sFTD and assess their potential biological impact.

## Subjects and methods

### Study population

Individuals included in the study were clinically diagnosed with a variant of FTD, including the subtypes bvFTD, SD/svPPA, PNFA/nfvPPA, FTD-MND, and FTD-unspecified (i.e., if individuals were diagnosed with FTD but could not be assigned to a specific subtype; see introduction section for acronym definitions). Diagnoses were made according to international consensus criteria: Neary et al. (for FTD, until 2011), Rascovsky et al. (for bvFTD), Gorno-Tempini et al. (for PPA, svPPA and nfvPPA), and Strong et al. (for FTD-MND).^3^^,^^4^^,^^5^^,^^17^ Individuals with logopenic variant PPA were excluded because of its major association with AD.

A previous FTD cohort, divided into discovery (cohort I) and replication (cohort II) sets,^14^ was further elaborated by (1) accruing updated metadata leading to the exclusion of individuals that did not meet the diagnostic criteria detailed above, or for which updates were not provided, and (2) inclusion of additional sets of controls. A new cohort of samples (cohort III) was progressively collected between 2016 and 2019 by clinicians and research groups based in Europe (Belgium, France, Germany, Italy, the Netherlands, Norway, Slovenia, Spain, Sweden, and UK) and North America (USA and Canada). Each contributing site obtained written informed consent for the samples to be part of this genetic study (IRB approval #9811/001). All samples (cohorts I, II, and III) were progressively sent as extracted DNA from tissues (blood and/or brain) and stored at −80°C upon receipt (see also Ferrari et al., 2014^14^). The bulk of the control samples used in this study (from France, Germany, Italy, the Netherlands, Spain, Sweden, UK, and USA) was available through a previous study,^14^ and additional control data were obtained from collaborators (at National Institutes of Health, USA and University College London, UK) and genotyped during the cohort III genotyping iterations (also including additional controls from Italy, Norway, and Slovenia). Overall, the control population at hand consisted of 16,821 samples (from France, Germany, Italy, the Netherlands, Spain, Sweden, UK, USA, Norway, and Slovenia), free of neurological illness at the time of sampling, and matched to cases based on population ancestry (see also QC—Samples below).

### Cohorts

Before quality control checks (QCs), cohort I included 2,026 cases and 8,387 controls, cohort II 1,121 cases and 5,091 controls, and cohort III 2,504 cases and 3,343 controls. The three cohorts were independently QCed (see below), leaving 2,006 cases and 8,350 controls for cohort I, 1,090 cases and 5,067 controls for cohort II, and 2,315 cases and 3,230 controls for cohort III.

**Figure 1:**
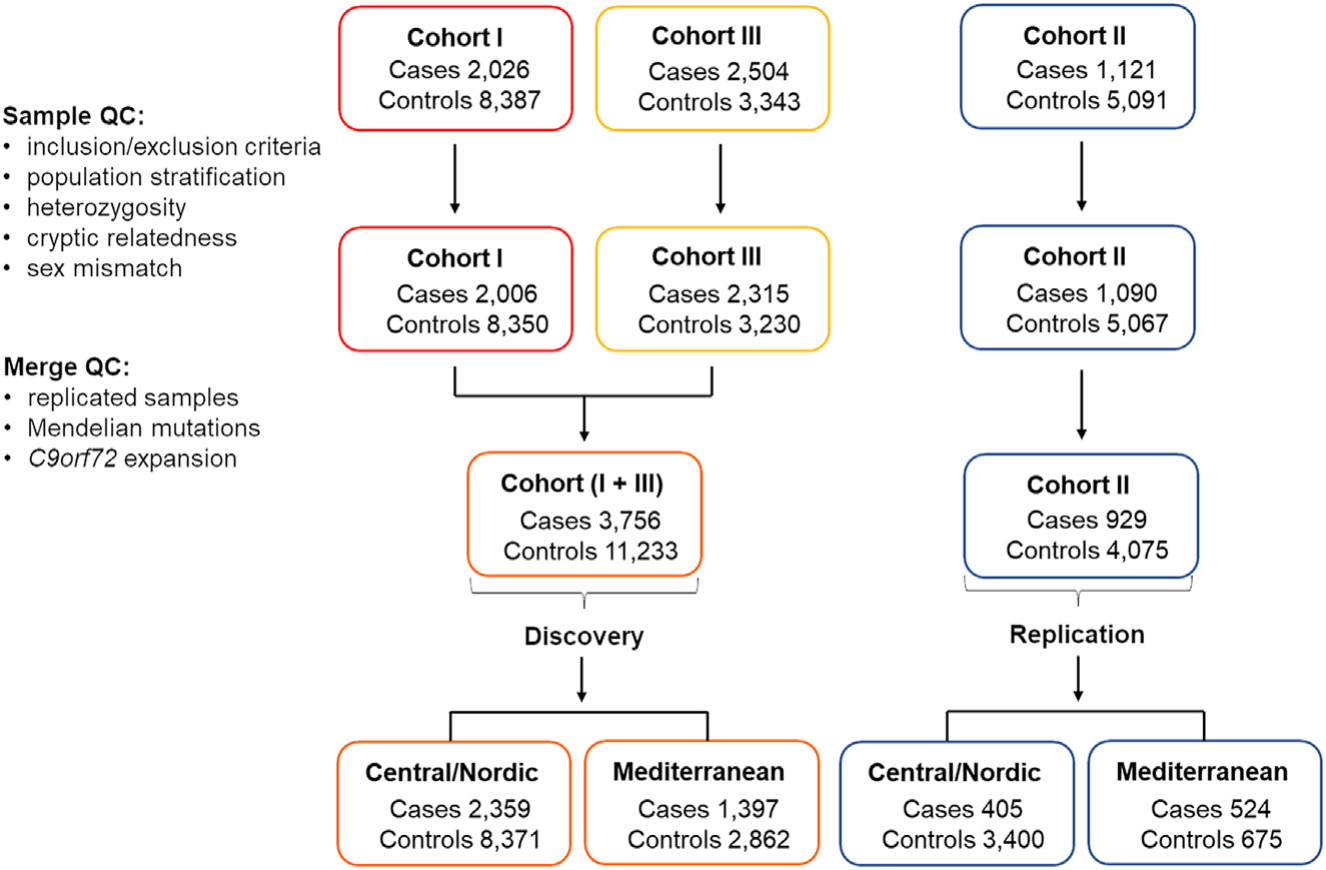
QC pipeline for the three cohorts contributing to discovery and replication The discovery and replication sets were further subdivided into Central/Nordic and Mediterranean Europeans (PCA-based genetically estimated ancestry) to assess potential population-specific disease risk loci.

Cohorts I and III (run on genotyping chips) were combined into a new discovery cohort. Cohort II (run on NeuroX array^14^^,^^18^) was used as replication set. Duplicated samples and samples known to carry Mendelian mutations in neurodegenerative genes (*APP* [MIM: 104760], *CHCHD10* [MIM: 615903], *CHMP2B* [MIM: 609512], *FUS* [MIM: 137070], *GRN*, *HNRNPA1* [MIM: 164017], *LRRK2* [MIM: 609007], *MAPT*, *NOTCH3* [MIM: 600276], *PSEN1* [MIM: 104311], *PSEN2* [MIM: 600759], *SERPINI1* [MIM: 602445], *SORL1* [MIM: 602005], *SQSTM1* [MIM: 601530], *TMEM106B*, *TBK1* [MIM: 604834], *VCP* [MIM: 601023]), or a *C9orf72* pathogenic expansion, were removed from the analyses, leading to the following final study cohorts (before sample QC): 3,756 cases and 11,233 controls for the discovery phase (cohorts I + III) and 929 cases and 4,075 controls for the replication phase (cohort II). A breakdown of samples (and FTD subtypes) is shown in Table S1. The three cohorts, the QC procedures, and the discovery and replication sets are shown in Figure 1. Of note, given the small number of samples for some of the FTD subtypes (SD, PNFA, and FTD-MND), subtype analysis was not informative, and therefore it is not reported.

### Sample genotyping

Cohort I cases had been genotyped on either 660K or Omni-Express Illumina array chips.^14^ Cohort II cases had been genotyped on the NeuroX array.^14^^,^^18^ Cohort III cases were genotyped on NeuroChip.^19^ Samples were genotyped at the Laboratory of Neurogenetics of the National Institute on Aging, NIH or at the core facility at the Institute of Child Health, UCL (UCL Genomics). All arrays were run on the Illumina Infinium platform as per the manufacturer’s instructions. Control samples had been genotyped using a variety of array chips, including 330K, 550K, 660K, Omni-Express, NeuroX, and NeuroChip, and cohorts I and II were previously genotyped as per Ferrari et al., 2014;^14^ cohort III samples were genotyped within the current study (IRB approval #9811/001). Data were QCed following standard procedure using samples with genotyping call rate ≥95% and markers with GenTrain score ≥0.7.

### QC—Samples

Sample-level QC was performed before carrying out separate imputation for each of the three cohorts. Genotypes were used to inform on population substructure via principal-component analysis (PCA). Linkage disequilibrium (LD)-pruned markers with a 95% genotyping rate (less than 5% missing), Hardy-Weinberg equilibrium exact test (HWE) *p* value ≥1 × 10^−10^ midp,^20^ and minor allele frequency (MAF) ≥0.01 were used to assess ancestry via PCA against HapMap Phase3 (hapmap3_r3_b36_fwd.consensus.qc.poly). This analysis allowed us to address population substructure and led to the exclusion of population outliers; briefly, PCA on the three different cohorts was performed, and overlap with European samples from HapMap was used to identify outliers (see details in Figure S1).

We also assessed samples’ potential contamination by evaluating individuals’ heterozygosity (removing samples with inbreeding coefficient estimates outside 4 standard deviations from the mean distribution) and cryptic relatedness (removing sample pairs with estimated identity by descent = PI_HAT greater than 0.125) using a set of LD-pruned high-quality SNPs (markers with a 95% genotyping rate, HWE ≥1 × 10^−10^ midp, and MAF ≥0.01). Finally, we excluded samples with a possible mismatch for sex by assessing the X chromosome heterozygosity (for cohorts II and III only, as for cohort I genotyping for the X chromosome was not available).

### QC—Markers

Variant-level QC cleaning was performed before imputation for each of the three cohorts. Palindromic markers and markers with missing call rates exceeding 5% were removed. Finally, we assessed differences between case and control genotype data via non-random missingness, excluding markers with Bonferroni’s corrected *p* values <0.1 (indicating significant differential missingness between cases and controls).

### Imputation

The three cohorts were imputed separately. Cohort I was converted from GRCh36/hg18 to GRCh37/hg19 using the LIFTOVER tool prior to imputation. We imputed markers through the Michigan server (https://imputationserver.sph.umich.edu/) using the following specifications and thresholds: Minimac4; HRC reference panel (GRCh37/hg19); imputation filter rsq 0.3; Eagle v.2.4 phasing; European (EUR) population. Multiallelic and palindromic variants were removed following imputation. Post-imputation dataset sizes were as follows: cohort I, ∼22 M markers; cohort II, ∼5 M markers; cohort III, ∼21 M markers. Imputation for cohort II resulted in a smaller number of imputed markers due to this cohort being genotyped on the NeuroX array, an exome chip not originally designed for imputation.^14^^,^^18^

### Discovery cohort merge

Relatedness across the three cohorts was evaluated using genotyped-only communal markers (cohorts I ꓵ II = 7,480 genotyped communal markers, II ꓵ III = 10,328, I ꓵ III = 94,773). A set of LD-pruned high-quality SNPs (markers with a 95% genotyping rate, HWE ≥1 × 10^−10^ midp, and MAF ≥0.01) was used to evaluate cryptic relatedness (removing sample pairs with estimated identity by descent = PI_HAT greater than 0.125). Cohort I and III post-imputation were then combined using the overlapping markers as discovery cohort (3,756 cases and 11,233 controls; ∼18 M markers genotyped and imputed), while cohort II was used as replication cohort (929 cases and 4,075 controls; ∼5 M markers genotyped and imputed) (Figure S2).

### Association analyses

We performed association analysis using markers with MAF >1% and HWE >10^−4^ midp through the PLINK case-control logistic regression association analysis with 20 principal components (PCs), sex, and study cohort (I or III) as covariates. The top markers were confirmed by running a similar association analysis in MAOS (https://dlin.web.unc.edu/software/maos/) using 20 PCs, sex, study cohort (I or III), and FTD subtypes as covariates.^21^ The same analytical pipeline was applied to the replication cohort. Markers of interest were meta-analyzed (discovery + replication) using METAL (run with STDERR scheme).^22^

Genome-wide significant markers were annotated using the Ensembl Variant Effect Predictor (VEP). Frequencies for 1000 Genomes (European samples) were used to further control for marker frequencies in the general population: a variation of >15% between our controls and the European general population as per 1000 Genomes was used as threshold to exclude variants from the current study. When single populations from 1000 Genomes were used, they were selected as follows: GBR = British in England and Scotland; CEU = Northern Europeans from Utah; IBR = Iberian populations in Spain; TSI = Tuscans from Italy.

The Bonferroni threshold for genome-wide significance was p ≤ 5 × 10^−8^.^23^ Variants with *p* values between 1 × 10^−5^ and 5 × 10^−8^ were reported as suggestive (only loci containing ≥20 markers in those *p* value ranges).

### Heritability and genetic correlation analyses

We used LD score regression (LDSC)^24^ to derive an SNP-based heritability estimate (h^2^) for the discovery FTD summary statistics (3,756 cases and 11,233 controls). The analysis was performed using the pre-computed European SNP LD scores (https://data.broadinstitute.org/alkesgroup/LDSCORE/eur_w_ld_chr.tar.bz2).

Then, LDSC was employed for estimation of genetic correlation (rg) between FTD and publicly available genome-wide association study (GWAS) summary statistics of five neurodegenerative disorders: (1) clinical AD GWAS of 63,926 samples (AD)^25^; (2) AD clinical/proxy GWAS and related dementias (ADRD) of 487,511 samples^26^; (3) Parkinson Disease GWAS (PD; MIM: 168600) of 1,474,097 samples^27^; (4) amyotrophic lateral sclerosis GWAS (ALS; MIM: 612069) of 138,086 samples^28^; and (5) Lewy body dementia GWAS (LBD; MIM: 127750) of 6,618 samples.^29^

BUHMBOX (https://software.broadinstitute.org/mpg/buhmbox/) was run according to Han et al., 2016.^30^ SNPs associated with AD^25^ were extracted in the *APOE* (MIM: 107741) region and filtered to remove FTD SNPs with *p* < 0.05. The remaining SNPs were clumped (with r^2^ = 0.1 in 10,000-kb window), resulting in 64 SNPs for the analysis.

### Locus analysis

Risk loci at chromosome 17 (*MAPT* region) and chromosome 19 (*APOE* region) were further characterized by extracting the genotypes of the following markers: rs17650901 (A:G) and rs242557 (G:A) on chromosome 17, where the A alleles tag the H1 and H1c clade, respectively,^31^^,^^32^ and rs429358 (C:T) and rs7412 (T:C) on chromosome 19 to assess the *APOE* alleles (C/C, C/C = Ɛ4/Ɛ4; T/T, T/T = Ɛ2/Ɛ2; and T/T, C/C = Ɛ3/Ɛ3). Additional controls for the *APOE* markers were downloaded from the 1000 Genomes Project using the Ensembl Genome Browser and obtained from an independent cohort of controls (731 Italian controls and 347 Central/Nordic European controls). Differences in Ɛ allele counts were assessed using Pearson’s χ^*2*^ test with Yates’ continuity correction.

### Functional analysis

We used the full distribution of SNP *p* values and the top markers (post joint analysis) to further characterize biological and functional effects, including potential effects on expression and splicing, using the GTEx (https://gtexportal.org/) and Functional Mapping and Annotation (FUMA) (https://fuma.ctglab.nl/)^33^ platforms.

### Software

All analyses were performed using R (R v.3.5.2), R studio (R v.3.6.2, studio v.1.2.1335), PLINK v.1.9, MAOS (http://dlin.web.unc.edu/files/2011/08/maos-1.2-linux.tar_.gz), and METAL (version for Windows, released March 25, 2011). LD score regression was run using LDSC v.1.0.1 (https://github.com/bulik/ldsc).

## Results

### Discovery association analyses

We performed GWAS analysis for the discovery cohort (3,756 cases and 11,233 controls). The genomic inflation factor (λ) was 1.035 (λ_1000_ = 1.006) (Figure S3). 1,886 markers, mapping to the *MAPT* (1,880 markers; chr17:43,572,419–44,862,347 [GRCh37/hg19]) and *APOE* (6 markers; chr19:45,387,596–45,396,144 [GRCh37/hg19]) loci, were genome-wide significant.

**Figure 2:**
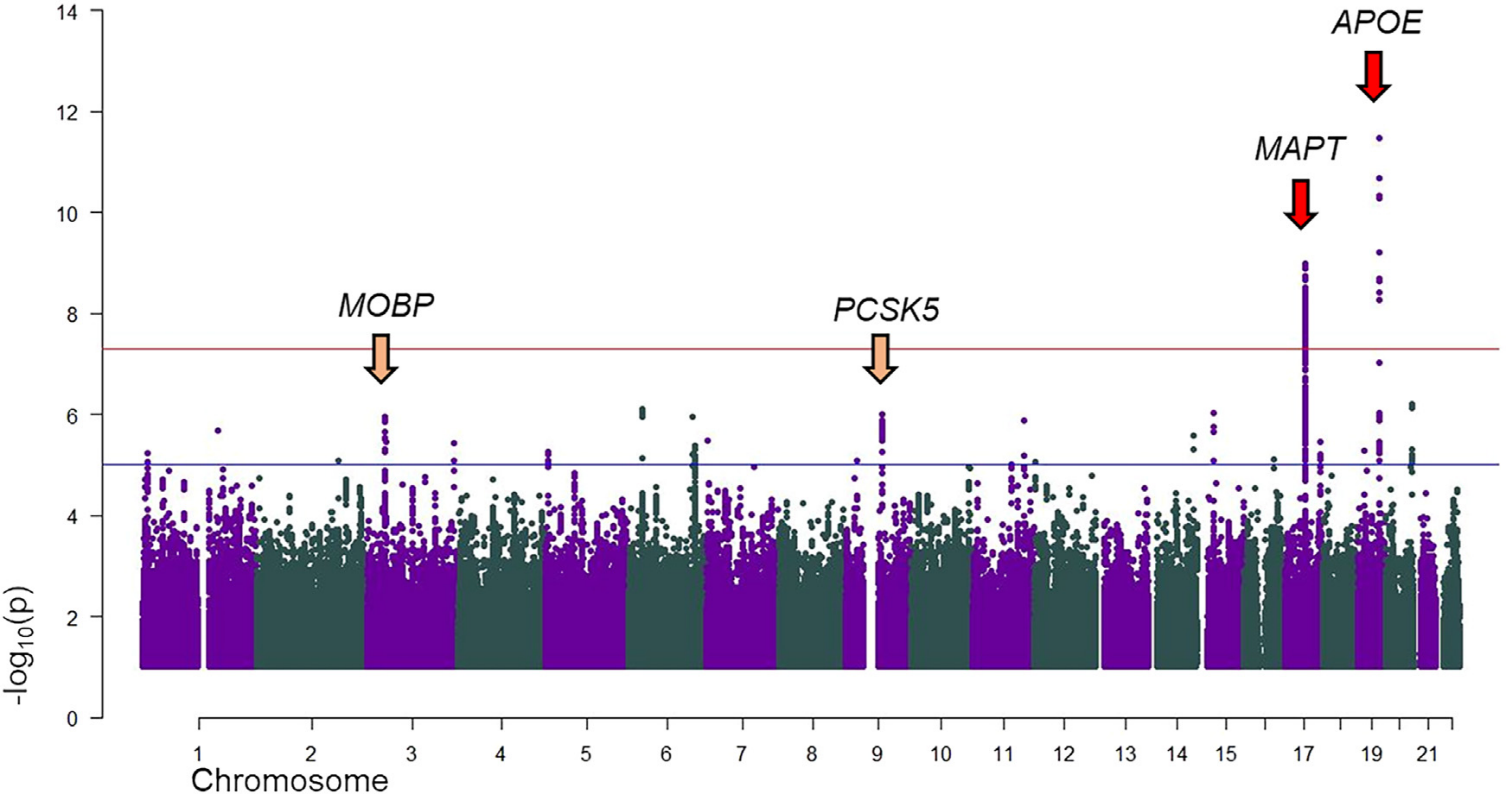
Discovery phase: Manhattan plot Red arrows identify genome-wide-significant signals (chromosomes 17 and 19); yellow arrows identify suggestive towers (10^−8^ < *p* > 10^−5^) including at least 20 markers (chromosomes 3 and 9). The plot is cut at −log_10_(*p*) = 1. The gene symbols represent the locus and do not necessarily imply functional/biological relevance.

The SNPs with the lowest *p* values were rs199443 (*MAPT* locus; *p* = 1.03 × 10^−9^; β = 0.229 [C = risk allele, major allele]; SE = 0.037) and rs6857 (*APOE* locus; *p* = 6.2 × 10^−10^; β = 0.239 [T = risk allele, minor allele]; SE = 0.039) (Figure 2; Table 1; Table S2). After conditioning the regional analyses on the index SNPs, each association disappeared, suggesting there is a unique signal per locus (Figures S4A and S4B). To further support our discovery findings, we used another statistical method (MAOS; see subjects and methods), which allowed us to run five different association analyses, assessing each subtype, accounting for the same control sets, and meta-analyzing the outcomes. The genome-wide-significant hits shown above were confirmed and displayed improved statistics (Table S3).

**Table 1:** Top marker summary statistics at the significant loci

**rs number**	**Chr**	**Base pair**	**Locus**	**A1/A2**	**Discovery**								**Replication**							**Joint Analysis**					
					**ImpScore (rsq)**^1^^,^^2^	**Minor allele frequency**			**PLINK**				**ImpScore (rsq)**	**Minor allele frequency**		**PLINK**				**METAL**					
						**Controls**	**Cases**	**1000G EUR-**	**Beta**	**OR**	**SE**	***p* value**		**Controls**	**Cases**	**Beta**	**OR**	**SE**	***p* value**	**Beta**	**OR**	**SE**	***p* value**	**I**^**2**^	**HetPVal**
rs199443	17	44,819,565	*MAPT*	T/C^∗^	0.96, 0.96	0.224	0.187	0.223	0.229	1.257	0.037	1.03E-09	genotyped	0.220	0.210	0.267	1.306	0.077	5.34E-04	0.236	1.266	0.034	2.50E-12	0	6.53E-01
rs6857	19	45,392,254	*APOE*	T^∗^/C	0.96, 0.98	0.151	0.179	0.165	0.239	1.270	0.039	6.22E-10	0.99	0.161	0.181	0.249	1.283	0.072	5.01E-04	0.241	1.273	0.034	1.31E-12	0	9.00E-01
rs1009966	3	39,473,591	*MOBP*	G/A^∗^	0.93, 0.97	0.475	0.433	0.460	0.144	1.155	0.030	1.12E-06	0.77	0.469	0.413	0.157	1.17	0.057	6.18E-03	0.147	1.158	0.026	2.36E-08	0	8.47E-01

Discovery, replication, and joint analysis statistics are shown for the top markers (smallest *p* value) at the significant loci. A1, minor allele; ^∗^, risk allele; ImpScore (rsq),^1^^,^^2^ imputation scores in phases I and III combined in discovery; 1000G, 1000 Genomes.

We identified six suggestive signals comprising at least four markers with *p* < 1 × 10^−5^ and sought to take two of them (comprising at least 20 markers with *p* < 1 × 10^−5^) forward for replication and joint analysis to screen for their potential relevance (Table S4): chromosome 3 (top SNP rs13081054; myelin-associated oligodendrocyte basic protein [*MOBP*; MIM: 600948] locus) and chromosome 9 (top SNP rs76573513; *PCSK5* [MIM: 600488] locus).

### Replication and joint analyses

The following top markers in discovery analysis (*n* = 6 for the *APOE* locus; *n* = 1,880 for the *MAPT* locus; *n* = 29 for the *MOBP* locus) were present in the replication set (see also Tables S2 and S4).

In the replication set, the chromosome 17 and chromosome 19 top SNPs (rs199443 and rs6857, respectively) reached *p* value = 5.3 × 10^−4^ (β = 0.267; SE = 0.077) and *p* value = 5 × 10^−4^ (β = 0.249; SE = 0.072), respectively. Following joint analysis, each marker was genome-wide significant (*p* = 2.5 × 10^−12^; β = 0.236; SE = 0.034 for rs199443 [*MAPT* locus] and *p* = 1.31 × 10^−12^; β = 0.241; SE = 0.034 for rs6857 [*APOE* locus]), with the effect being in the same direction (Table 1; Table S2).

For the suggestive signals, only markers on chromosome 3 were available in the replication set (due to lower imputation coverage for the NeuroX exome-chip; see subjects and methods): rs1009966 was replicated and reached lowest *p* value (genome-wide significant) after joint analysis (*p* value = 2.36 × 10^−8^; β = 0.147; SE = 0.026), with the effect being in the same direction (Table S4).

### *MAPT* and *APOE* loci

We further characterized the *MAPT* locus in the discovery cohort using rs17650901 (A:G) and rs242557 (G:A), where the A alleles tag the H1 and H1c clade, respectively. The frequencies of the A allele and the homozygous A/A genotype were significantly increased in cases compared to controls for rs17650901 (*p* = 1.9 × 10^−9^ and *p* = 7.7 × 10^−9^, respectively) and rs242557 (*p* = 5 × 10^−3^ and *p* = 8.8 × 10^−3^, respectively), suggesting association of the H1/H1 haplotype and H1c clade with sFTD (Table 2).

**Table 2:** Haplotype analysis at the *MAPT* locus

**Marker**	**Genotype**	**Haplotype**	**Cases**		**Controls**		**χ**^**2**^**A**	**χ**^**2**^**AA**
			**Count**	**Freq**	**Count**	**Freq**		
chr17:44039691A>G (rs17650901)	G/G	H2/H2	163	0.04	644	0.06	1.9 × 10^−9^	7.7 × 10^−9^
	G/A	H1/H2	1,221	0.33	4,098	0.36		
	A/A	H1/H1	2,372	0.63	6,491	0.58		
chr17:44019712G>A (rs242557)	A/A	H1c/H1c	509	0.14	1,338	0.12	5 × 10^−3^	8.8 × 10^−3^
	A/G	H1/H1c	1,733	0.46	5,148	0.46		
	G/G	H1/H1	1,514	0.40	4,747	0.42		

The H1 and H2 haplotype distribution is shown for cases and controls. Count, number of subjects; freq, frequency of the haplotype.

The significant markers at chromosome 19 revealed a (genetically estimated) ancestry-related difference in allele frequencies (Table 3): whereas individuals with FTD showed similar frequencies for the chromosome 19 markers regardless of ancestry, Mediterranean European controls showed remarkably decreased frequencies compared to Central/Nordic European controls (Table 3). This trend was further confirmed in an independent cohort of 731 Italian and 347 Continental European controls (Table 3; additional characterization of the *APOE* locus [*APOE Ɛ4* alleles] is included in the supplemental information [Note S1]).

**Table 3:** Ancestry-related difference at the *APOE* locus

						**Minor allele frequency**								
						**Discovery**		**1000G**	**Discovery Nordic/Central European**		**Independent Nordic/Central European cohort**	**Discovery Mediterranean**		**Independent Italian cohort**
Chr	rs number	Base pair	*p* value	OR	Allele	Cases	Controls	EUR	Cases	Controls	Controls	Cases	Controls	Controls
19	rs6857	45,392,254	6.22E-10	1.27	T	0.1793	0.1508	0.165	0.1855	0.1651	0.1686	0.1689	0.109	0.1204
19	rs12972970	45,387,596	2.10E-09	1.28	A	0.1531	0.1283	0.1352	0.1598	0.1422	0.147	0.1417	0.0877	0.1026
19	rs34342646	45,388,130	2.35E-09	1.276	A	0.1571	0.1321	0.1392	0.1636	0.1455	0.147	0.146	0.09294	0.1026
19	rs71352238	45,394,336	3.87E-09	1.273	C	0.155	0.1304	0.1332	0.1624	0.1444	0.1527	0.1424	0.08945	0.1033
19	rs2075650	45,395,619	5.29E-09	1.272	G	0.1523	0.128	0.1312	0.1598	0.1427	0.1455	0.1396	0.08491	0.1033
19	rs11556505	45,396,144	5.50E-09	1.272	T	0.1519	0.1278	0.1312	0.1594	0.1426	0.1455	0.1392	0.08456	0.1033

The allele frequencies of the significant markers at the APOE locus are shown in different cohorts (the entire discovery cohort; the 1000 Genomes European cohort, the discovery cohort divided into Central European/Nordic and Mediterranean European populations, and independent samples of Central European/Nordic and Mediterranean European ancestry). 1000G, 1000 Genomes.

### Central/Nordic and Mediterranean European independent analyses

To better understand the population-specific contribution to the discovery analysis results, we split the discovery cohort into Central/Nordic (2,359 cases and 8,371 controls) and Mediterranean Europeans (1,397 cases and 2,862 controls), based on genetically estimated ancestry (PCA based), and performed association analysis for these two sub-cohorts separately (Figures S5 and S6). The genomic inflation factors were λ = 1.0135 and λ = 1.0356, respectively.

**Figure 3:**
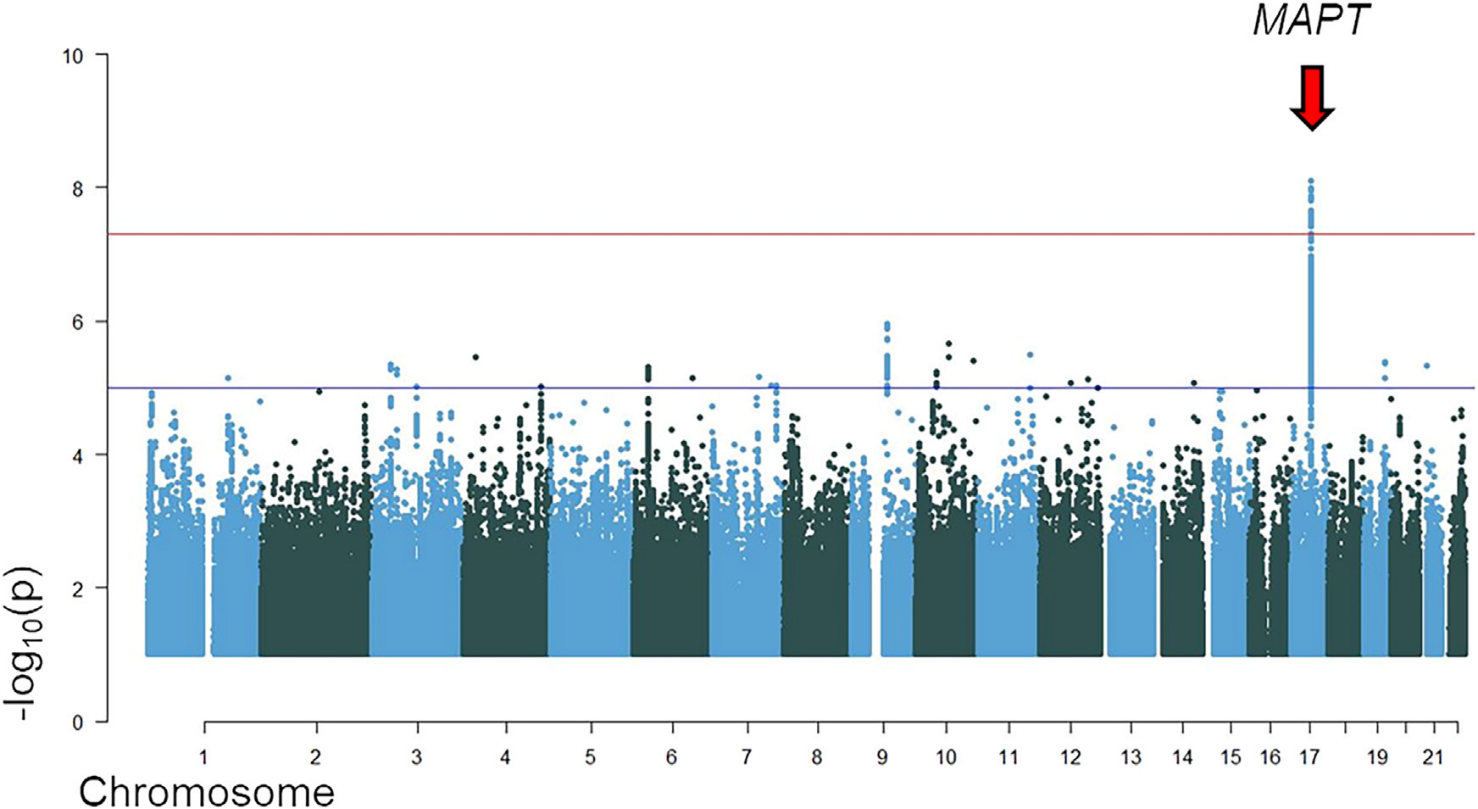
Manhattan plot for discovery-phase Central/Nordic European cohort Red arrow indicates the genome-wide significant signal (chromosome 17). The plot is cut at −log_10_(*p*) = 1. The gene symbols represent the locus and do not necessarily imply functional/biological relevance.

Association analysis for the Central/Nordic European cohort revealed one genome-wide-significant signal on chromosome 17 at the *MAPT* locus (rs199443; *p* = 1.08 × 10^−8^; β = 0.256 [C = risk allele, major allele]; SE = 0.045). In the replication set (also subdivided in Central/Nordic [405 cases and 3,400 controls] and Mediterranean Europeans [524 cases and 675 controls]), the chromosome 17 top SNP reached *p* value = 2.19 × 10^−2^ (β = 0.249; SE = 0.109), and joint analysis confirmed genome-wide-significant statistics (*p* = 8.02 × 10^−10^; β = 0.256; SE = 0.042), with the effect being in the same direction (Figure 3; Table 4). The *APOE* locus signal on chromosome 19 (rs6857) was not genome-wide significant (*p* value = 7.9 × 10^−5^) in the discovery Central/Nordic European cohort; it reached *p* value = 2.55 × 10^−2^ in the replication set and remained not significant (*p* = 6.8 × 10^−6^; β = 0.183; SE = 0.041) after joint analysis (Table 4).

**Table 4:** Top marker summary statistics for the Central European/Nordic and Mediterranean European cohorts

						**Discovery**										**Replication**						**Joint Analysis**			
						**Minor allele frequency**						**PLINK**				**Minor allele frequency**		**PLINK**				**METAL**			
Cohort	rs number	Chr	Base pair	Locus	A1/A2	Controls	Cases	1000G IBR	1000G TSI	1000G GBR	1000G CEU	OR	Beta	SE	*p* value	Controls	Cases	OR	Beta	SE	*p* value	Beta	OR	SE	*p* value
Central European/Nordic	rs199443	17	44,819,565	*MAPT*	T/C	0.215	0.175	-	-	0.231	0.197	1.293	0.256	0.045	1.08E-08	0.211	0.179	1.283	0.249	0.109	2.19E-02	0.256	1.291	0.042	8.018E-10
	rs6857	19	45,392,254	*APOE*	T/C	0.165	0.186	-	-	0.170	0.197	1.193	0.176	0.045	7.90E-05	0.168	0.203	1.235	0.211	0.095	2.55E-02	0.183	1.201	0.041	6.799E-06
Mediterranean European	rs199443	17	44,819,565	*MAPT*	T/C	0.250	0.207	0.262	0.308	-	-	1.184	0.170	0.070	1.52E-02	0.265	0.234	1.142	0.133	0.108	2.19E-01	0.159	1.172	0.059	6.80E-03
	rs6857	19	45,392,254	*APOE*	T/C	0.109	0.169	0.164	0.117	-	-	1.457	0.376	0.082	4.00E-06	0.124	0.165	1.281	0.248	0.126	4.88E-02	0.338	1.402	0.068	7.67E-07

Discovery, replication, and joint analysis statistics are shown for the top markers (smallest *p* value) at the significant loci. 1000G, 1000 Genomes MAF; GBR, British in England and Scotland; CEU, Northern Europeans from Utah; IBR, Iberian populations in Spain; TSI, Tuscans from Italy.

Association analysis for the Mediterranean cohort did not yield significant results (probably because of power issues due to the small Mediterranean cohort size). However, it is worth noting that, although the signal on chromosome 17 showed *p*_joint_ = 6.80 × 10^−3^, the signal on chromosome 19 was suggestive (*p*_joint_ = 7.67 × 10^−7^; β = 0.338; SE = 0.068) (Figure S7; Table 4).

**Figure 4:**
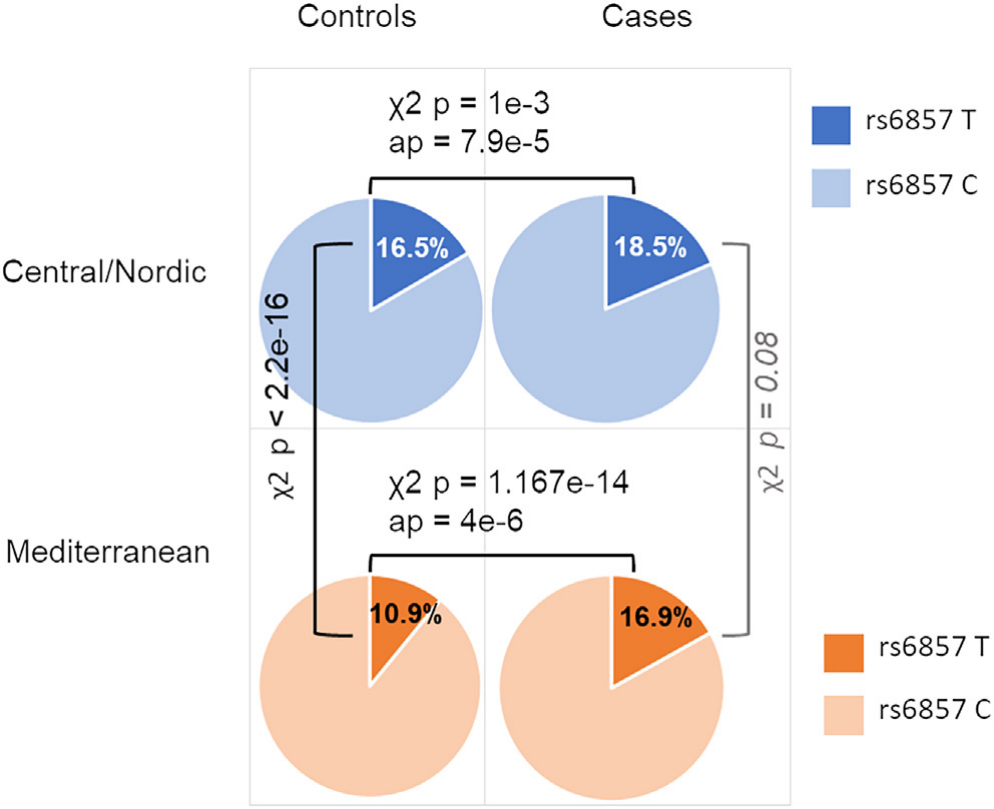
Risk allele frequencies of top marker (rs6857) at the chromosome 19 locus in the Central/Nordic and Mediterranean European discovery cohorts Uncorrected *p* values calculated via χ^2^ are reported; association *p* values (ap) are reported for the case-control comparisons.

To further shed light on the *APOE* locus signal on chromosome 19 (rs6857) in the two cohorts, we performed χ^2^ test (cases vs. controls) for rs6857 in the Central/Nordic European and Mediterranean European cohorts (Figure 4): the unadjusted *p* value for the former group was 1 × 10^−3^, while for the latter it was 1.2 × 10^−14^ (and driven by the control frequencies).

In summary, taken together, these data suggest that, in our extended cohort, the signal at the *MAPT* locus appeared to be mainly driven by the Central/Nordic European cohort and that at the *APOE* locus by the Mediterranean European cohort.

### Functional analysis

We found no meaningful impacted biological pathways using the full distribution of SNP *p* values (Table S5). The assessment of potential effects on expression and splicing (e/sQTL) exerted by the risk markers highlighted in the current study revealed the following (Table S6): (1) there was no significant effect on expression or splicing of in-*cis* genes for the *APOE* locus marker (rs6857); (2) the *MAPT* locus marker (rs199443) revealed effects on both expression and splicing, in *cis*, in brain cortex, affecting genes involved in transcription regulation (e.g., KANSL1 antisense RNA 1 [*KANSL1-AS1*; MIM: 612452]), protein trafficking (e.g., ADP ribosylation factor-like GTPase 17A [*ARL17A*]), and signal transduction (e.g., corticotropin-releasing hormone receptor 1 [*CRHR1*; MIM: 122561]), as well as *MAPT* itself; and (3) the top marker on chromosome 3 (rs1009966) revealed effects on expression in the cerebellum and splicing in brain cortex of the ribosomal protein SA gene (*RPSA*; MIM: 150370); the RPSA protein is involved in stabilizing ribosomal subunits and in the signal transduction as a cell surface receptor for laminin.^34^

### Heritability and genetic correlation analyses

Heritability estimation with LDSC regression for FTD summary statistics returned h^2^ = 0.118 (se = 0.02) (Table S7A). Genetic correlation of FTD with five other traits related to neurodegenerative diseases revealed positive significant correlation with all of them (*p* < 0.05), with the exception of Alzheimer disease-related dementia (ADRD) GWAS^26^ (see Table S7B). The largest genetic correlations were with LBD (rg = 0.91), ALS (rg = 0.71), and AD (rg = 0.55), and the overall results of this analysis indicated substantial shared genetic liability or potential misdiagnoses of FTD with all of the following conditions: LBD, ALS, AD, and PD. BUHMBOX was run to compare the *APOE* region (chr19:44.4–46.5 Mb) between AD and FTD, resulting in *p* = 0.006 and Mendelian randomization (MR) *p* = 5.8 × 10^−6^.

### Cross-check of markers across different neurodegenerative diseases

We sought to verify the statistics of key markers previously identified in other neurodegenerative conditions (etiologically close to FTD) in our current dataset (Table S8). The *TMEM106B* and *GFRA2* markers^11^^,^^12^ showed negligible association in our dataset (likely because of the current study design, i.e., clinical cohort excluding individuals with *GRN* mutations). Some of the significant ALS markers as shown in van Rheenen et al., 2021 and Nicolas et al., 2018^28^^,^^35^ reached *p* = 1.07 × 10^−3^ and *p* = 1.6 × 10^−4^ for the *UNC13A* (MIM: 609894) and *MOBP* loci markers (rs12973192 [proxy] and rs631312, respectively) with similar effects sizes (OR = 1.1), while the *C9orf72* marker (rs3849943, highly significant in ALS) showed negligible association in our dataset (probably because of the current study design, i.e., individuals with *C9orf72* expansion were excluded from the study). Most historically established AD markers—including rs6656401 and rs679515 (*CR1*; MIM: 120620), rs6733839 (*BIN1*; MIM: 601248), rs9331896 and rs11787077 (*CLU*; MIM: 185430), and rs6605556 (*HLA*)^26^^,^^36^^,^^37^—showed negligible association. *PICALM* (rs3851179; MIM: 603025) showed *p* = 6.5 × 10^−3^ and, interestingly, *MAPT* (rs199515) *p* = 1.1 × 10^−9^.^26^^,^^36^

In addition, for the *APOE* locus, we assessed several markers that were extensively reported as being genome-wide significant in AD (rs4420638, rs439401, and rs7412)^37^^,^^38^: one of these markers showed significant *p* value levels, though displaying smaller effect size in the current cohort (rs4420638; *p* = 9.6 × 10^−8^ with OR = 3.95 in AD and 1.2 in FTD), while the other two did not (rs439401; *p* = 8.1 × 10^−1^ and rs7412; *p* = 1.9 × 10^−4^). Conversely, we also sought to verify the *MOBP* hit reported as a novel potential FTD locus in the current work (rs1009966) in AD datasets: it showed negligible *p* values in two recent AD GWASs, i.e., *p* = 3.4 × 10^−1^ and 7.5 × 10^−1^.^25^,^26^ A previously reported hit at the *HLA* locus^14^ reached suggestive significance (rs9268877; *p* = 9.57 × 10^−7^). Furthermore, whereas several previously reported PSP and CBD risk variants^39^^,^^40^^,^^41^ showed negligible association, some PSP risk markers reached *p* values in the range of 10^−3^ (rs1411478 [*STX6*; MIM: 603944] and rs11568563 [*SLCO1A2*; MIM: 602883]) and 10^−4^ (rs1768208 [*MOBP*]) in our dataset. Finally, the *MAPT* locus risk variants previously reported in PD, AD, PSP, and CBD^26^^,^^39^^,^^40^^,^^42^ all resulted genome-wide significant in our dataset.

## Discussion

In the current work, we analyzed 4,685 sFTD cases and 15,308 controls, looking for common genetic determinants contributing to increased risk of sFTD. Compared to the previous work,^14^ we here increased sample size and improved the cohort (updated diagnoses) and provided insights highlighting a cluster of variants at the *MAPT* (rs199443; chromosome 17) and *APOE* (rs6857; chromosome 19) loci and a candidate locus on chromosome 3 (rs1009966 in the intergenic region between *RPSA* and *MOBP*) contributing to increased risk for sFTD by potentially mediating effects on expression and splicing of functionally relevant in-*cis* genes at the *MAPT*^43^ and *RPSA*-*MOBP* loci.

Further analysis of the *MAPT* locus suggested an increase of the H1/H1 haplotype and of the H1c clade in individuals with sFTD. This signature was previously reported in two small FTD cohorts from France and the UK,^44^^,^^45^ and it also appears to be consistent across different neurodegenerative conditions, as the H1 haplotype was shown to be associated with AD,^46^^,^^47^ CBD, PSP,^48^^,^^49^^,^^50^ and PD.^51^ This, and the fact that the *MAPT* locus risk variants previously reported in PD, AD, PSP, and CBD^26^^,^^39^^,^^40^^,^^42^ all resulted genome-wide significant in our dataset (and in complete and perfect LD with each other), supports the notion that the *MAPT* locus may be a common denominator at the crossroad of multiple etiologically close conditions such as FTD, AD,^26^ PSP, CBD,^50^ and PD.^52^

The association with the *RPSA*-*MOBP* locus appears of particular interest considering its involvement in PSP^39^ and ALS.^28^ Our reported marker with the lowest *p* value after joint analysis (rs1009966; *p* = 2.36 × 10^−8^, OR = 1.16) is in complete (D’ = 1) although not perfect (R^2^ = 0.3) LD in the European population (LDlink [https://ldlink.nih.gov/?tab=home] CEU 1000 Genomes, GRCh37) with the PSP (rs1768208) and ALS (rs631312) genome-wide-significant markers, suggesting potential pleiotropy at this locus across a subset of individuals with FTD, PSP, and ALS.

The signal at the *APOE* locus appeared to be population specific and driven by the control frequencies. More specifically, although the frequencies of the markers at the *APOE* locus were relatively similar in the Central/Nordic European (18.5%) and Mediterranean European (16.9%) cases making up the study cohort, they were significantly less frequent in Mediterranean (10.9%) compared to Central/Nordic European (16.5%) controls. The frequency patterns observed in our Central/Nordic vs. Mediterranean European controls were further supported by the 1000 Genomes cohort (IBR + TSI vs. CEU + GBR general populations), an independent cohort of controls (Italian and Central/Nordic Europeans), and a population-specific study analyzing *APOE* frequencies in the Treviso longevity (TRELONG) longitudinal study.^53^ All this taken together may suggest there being a genuine variation in the genetic architecture of these two (close yet different) European population groups at this locus.^54^^,^^55^ The link to the *APOE* locus could lead to different explanations/interpretations: (1) it may reflect a presence of individuals in our cohort with behavioral variant AD that mimics FTD^56^^,^^57^; (2) it may underpin comorbidities at play, given that some individuals with FTD coming to autopsy can show concurrent AD or vascular changes in addition to changes attributed directly to FTD^58^^,^^59^^,^^60^; (3) it may represent a genuine association pertaining to a population-specific subset of individuals with FTD. In relation to this latter point, it is worth noting that, per study design, we excluded all samples diagnosed with the logopenic variant in the current work (thus reducing potential diagnosis bias), and we verified that many of the historical AD risk loci (e.g., *CR1*, *BIN1*, *CLU*) reached negligible *p* values in our cohort, while the *MOBP* locus hit reported for FTD in the current work shows negligible *p* values in two recent AD GWASs. Moreover, a literature survey supports the notion of the involvement of the *APOE* locus in some forms of FTD.^52^^,^^61^^,^^62^^,^^63^^,^^64^

Previous work^14^ reported association with the *HLA* locus; in the current analyses, although genome-wide-significant levels are not reached, we show rs9268863 as the top SNP at the *HLA* locus (*p* = 7.86 × 10^−7^, OR = 1.2; A = risk allele) in the discovery analysis, with rs9268877 and rs9268863 being in complete (D’ = 1) and perfect (R^2^ = 1) LD in the European population (LDlink [https://ldlink.nih.gov/?tab=home] CEU 1000 Genomes, GRCh37). Clearly, despite other work supporting the notion of an involvement of immune system processes in the pathogenesis of sFTD,^65^ additional studies are needed to shed light on the level and type of involvement of the *HLA* locus in sFTD.

In summary, we report that the *MAPT* (pointing to the H1c clade), *APOE*, and *RPSA*-*MOBP* loci contribute to increased genetic risk of sFTD pathogenesis. Notably, the involvement of the *MAPT* locus in sFTD, and its association also with AD, PSP, PD, and CBD, strongly suggests potential common genetic pleiotropy for these neurological conditions at this locus. Moreover, our results pointing at the *RPSA*-*MOBP* locus shed light on an additional potential genetic overlap between FTD and PSP and between FTD and ALS. It is worth noting that the diagnosis of neurodegenerative diseases is challenging due to subtle overlap of clinical presentations^62^; therefore, future studies will need to be powered enough to allow for further assessment of pleiotropy vs. potential misdiagnosis (e.g., through structural equation modeling^66^). Moreover, an interesting way forward will be to not only continue addressing the genetic etiology of familial vs. sporadic FTD separately, but also broaden approaches by carefully designing studies to test for modifiers and the polygenic nature of Mendelian cases, provided adequate power.

Our study, however, gives grounds to the inference that FTD may be part of a wider spectrum of genetically mediated neurodegenerative conditions^67^ spanning from ALS to AD phenotypes and encompassing PSP, PD, and CBD. The current study also lays the foundations for future work aimed at further characterizing population-specific features of potential FTD-discriminant *APOE* haplotype(s) and the functional involvement and contribution of the *MAPT* H1c haplotype and *RPSA*-*MOBP* loci to pathogenesis in brain cortex of sporadic forms of FTD.

## Data and code availability

The accession number for the summary statistics (available for download from UCL Research Data Repository) is https://doi.org/10.5522/04/25600692.v1. Underlying participant-level data are available to potential collaborators, where individual-study data-access consent and pre-approved ethics are obtained to permit such data sharing: directly contact the site PIs (Note S2) to obtain both permission and consent to access their samples’ data.

## Supplemental information

Document S1. Figures S1–S7, Tables S1 and S5–S8, and Notes S1 and S2

Table S2. *MAPT* and *APOE* loci-significant markers

Table S3. Association analysis using MAOS (A1, minor allele; bold, risk allele)

Table S4. Summary of suggestive markers taken forward for replication

Document S2. Article plus supplemental information
