# Supplementary material for: Genome-wide analyses reveal a potential role for the MAPT, MOBP, and APOE loci in sporadic frontotemporal dementia
Source: Am J Hum Genet. 2024 Jun 17;111(7):1316–29. doi: 10.1016/j.ajhg.2024.05.017 (PMC11267522; doi:10.1016/j.ajhg.2024.05.017)
Supplement: Document S1. Figures S1–S7, Tables S1 and S5–S8, and Notes S1 and S2 [file mmc1.pdf]

The American Journal of Human Genetics, Volume *111*

Supplemental information

Genome-wide analyses reveal a potential role for the *MAPT*, *MOBP*, and *APOE* loci in sporadic frontotemporal dementia

Claudia Manzoni, Demis A. Kia, Raffaele Ferrari, Ganna Leonenko, Beatrice Costa, Valentina Saba, Edwin Jabbari, Manuela MX. Tan, Diego Albani, Victoria Alvarez, Ignacio Alvarez, Ole A. Andreassen, Antonella Angiolillo, Andrea Arighi, Matt Baker, Luisa Benussi, Valentina Bessi, Giuliano Binetti, Daniel J. Blackburn, Merce Boada, Bradley F. Boeve, Sergi Borrego-Ecija, Barbara Borroni, Geir Bråthen, William S. Brooks, Amalia C. Bruni, Paola Caroppo, Sara Bandres-Ciga, Jordi Clarimon, Rosanna Colao, Carlos Cruchaga, Adrian Danek, Sterre CM. de Boer, Itziar de Rojas, Alfonso di Costanzo, Dennis W. Dickson, Janine Diehl-Schmid, Carol Dobson-Stone, Oriol Dols-Icardo, Aldo Donizetti, Elise Dopper, Elisabetta Durante, Camilla Ferrari, Gianluigi Forloni, Francesca Frangipane, Laura Fratiglioni, Milica G. Kramberger, Daniela Galimberti, Maurizio Gallucci, Pablo García-González, Roberta Ghidoni, Giorgio Giaccone, Caroline Graff, Neill R. Graff-Radford, Jordan Grafman, Glenda M. Halliday, Dena G. Hernandez, Lena E. Hjermind, John R. Hodges, Guy Holloway, Edward D. Huey, Ignacio Illán-Gala, Keith A. Josephs, David S. Knopman, Mark Kristiansen, John B. Kwok, Isabelle Leber, Hampton L. Leonard, Ilenia Libri, Alberto Lleo, Ian R. Mackenzie, Gaganjit K. Madhan, Raffaele Maletta, Marta Marquié, Ales Maver, Manuel Menendez-Gonzalez, Graziella Milan, Bruce L. Miller, Christopher M. Morris, Huw R. Morris, Benedetta Nacmias, Judith Newton, Jørgen E. Nielsen, Christer Nilsson, Valeria Novelli, Alessandro Padovani, Suvankar Pal, Florence Pasquier, Pau Pastor, Robert Perneczky, Borut Peterlin, Ronald C. Petersen, Olivier Piguet, Yolande AL. Pijnenburg, Annibale A. Puca, Rosa Rademakers, Innocenzo Rainero, Lianne M. Reus, Anna MT. Richardson, Matthias Riemenschneider, Ekaterina Rogaeva, Boris Rogelj, Sara Rollinson, Howard Rosen, Giacomina Rossi, James B. Rowe, Elisa Rubino, Agustin Ruiz, Erika Salvi, Raquel Sanchez-Valle, Sigrid Botne Sando, Alexander F. Santillo, Jennifer A. Saxon, Johannes CM. Schlachetzki, Sonja W. Scholz, Harro Seelaar, William W. Seeley, Maria Serpente, Sandro Sorbi, Sabrina Sordon, Peter St George-Hyslop, Jennifer C. Thompson, Christine Van Broeckhoven, Vivianna M. Van Deerlin, Sven J. Van der Lee, John Van Swieten, Fabrizio Tagliavini, Julie van der Zee, Arianna Veronesi, Emilia Vitale, Maria Landqvist Waldo, Jennifer S. Yokoyama, Mike A. Nalls, Parastoo Momeni, Andrew B. Singleton, John Hardy, and Valentina Escott-Price

Figure S1

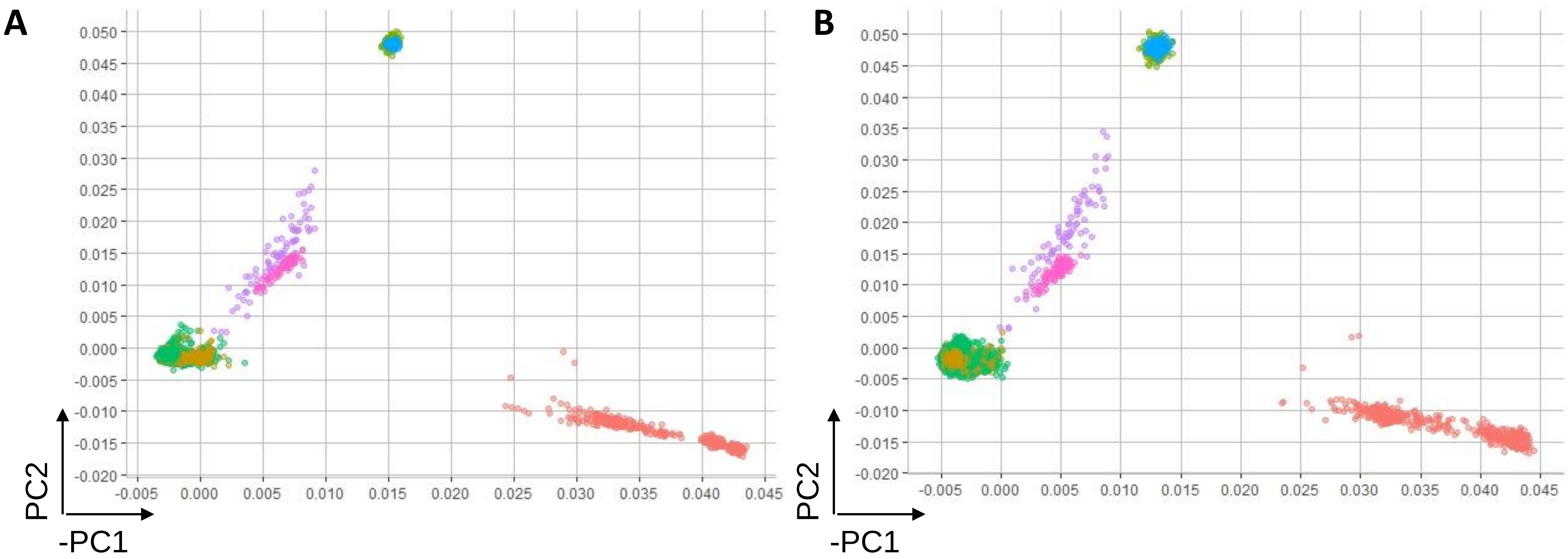

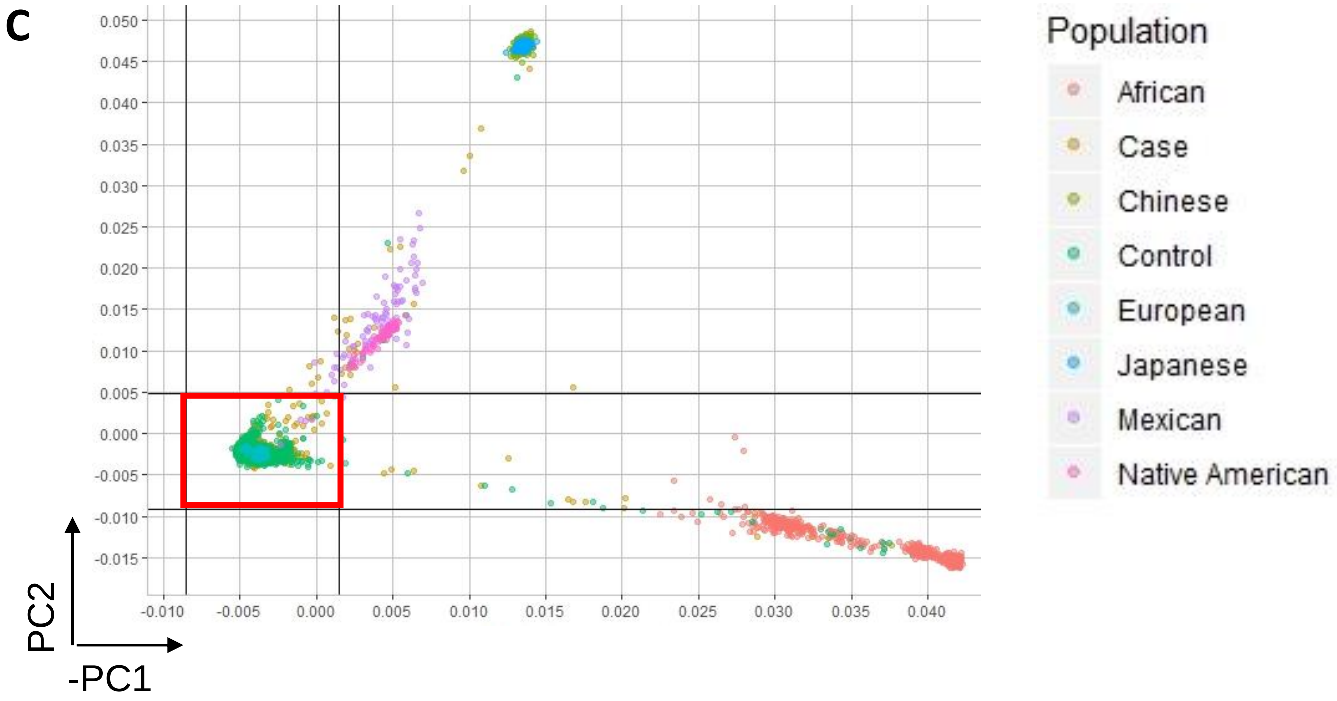

Figure S1 – *Principal component analysis on the 3 different cohorts. While cohorts I (A) and II (B) did not present outliers for population stratification with both cases and controls presenting with a relatively good overlap with European samples from HapMap, cohort-III (C) presented with a number of outliers that were removed by keeping only the samples (cases and controls) contained in the red square. We labelled outliers of the population with European ancestry individuals with > (or <) 1.5 standard deviation in component vector 1 and > 2.5 (or <) standard deviation for component vector 2*

Figure S2

A

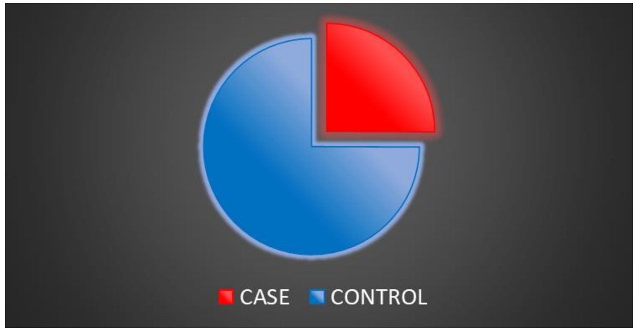

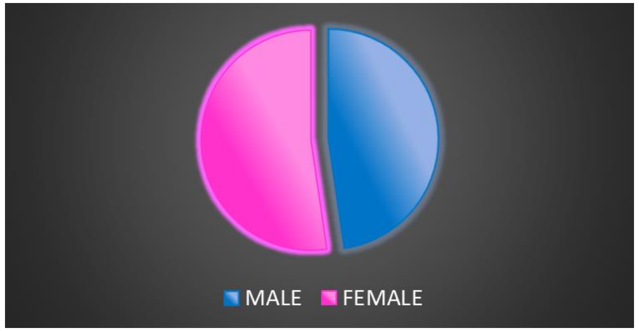

CASES: 3,756
CONTROLS: 11,233

MALES: 7,147
FEMALES: 7,842

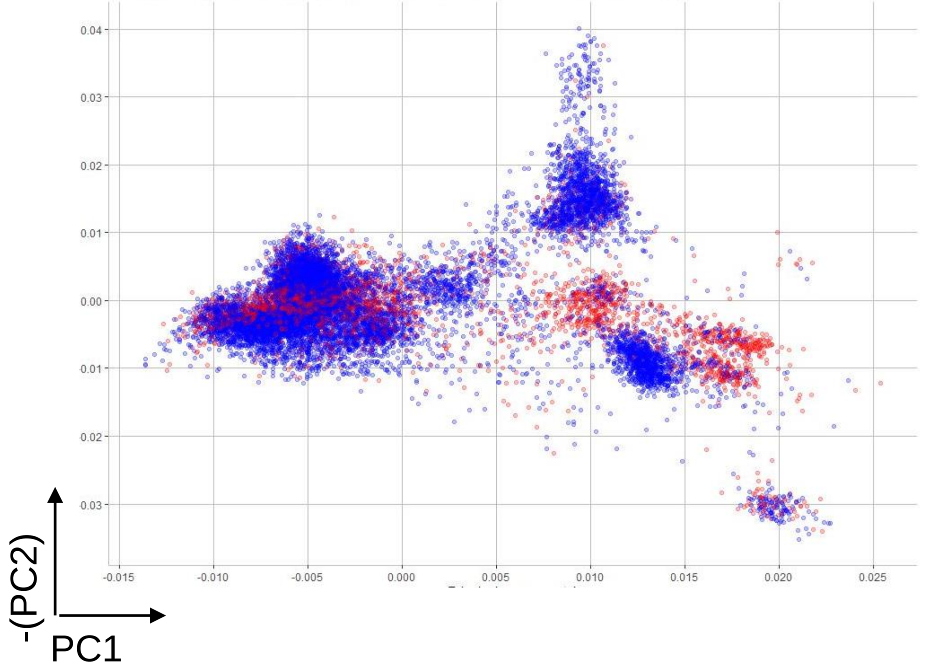

B

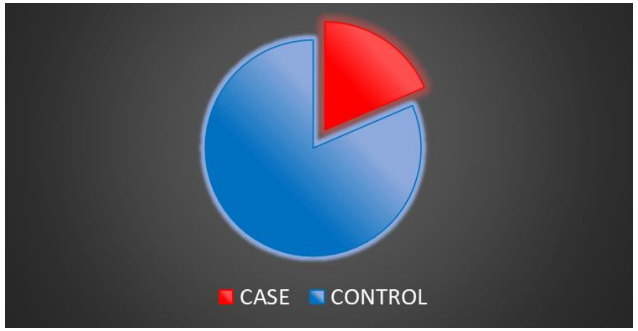

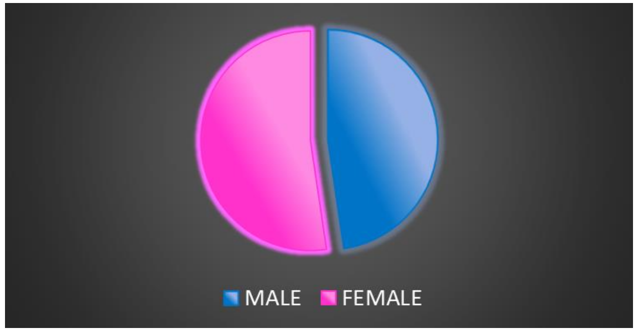

CASES: 929
CONTROLS: 4,075

MALES: 2,385
FEMALES: 2,619

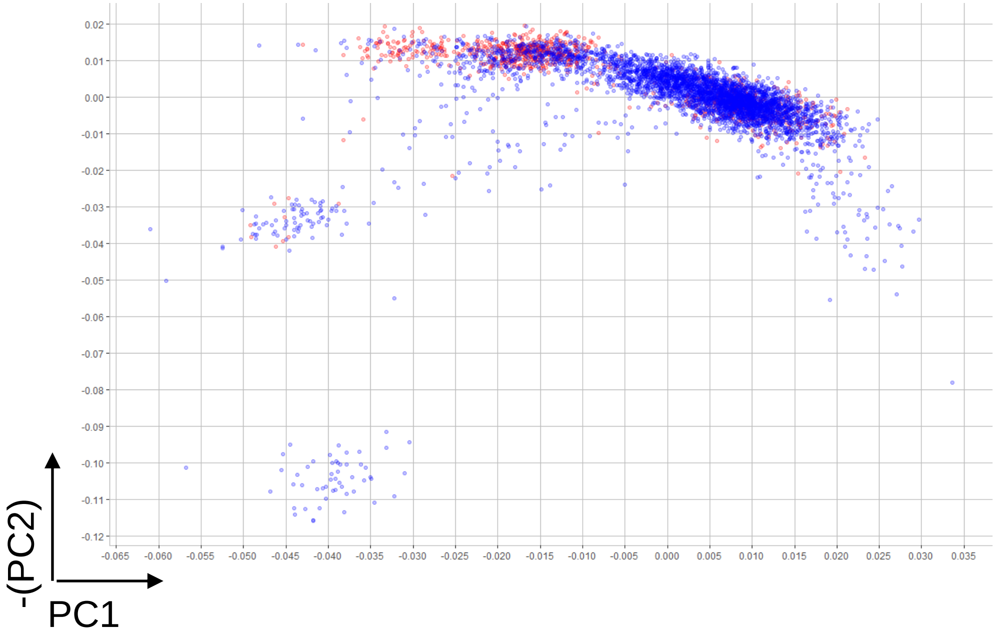

Figure S2 – *features of the discovery (A) and replication (B) cohorts. Division case/control and male/female; PC1 and PC2 were plotted and samples color-coded based on case/control status to verify similar population stratifications in cases in controls*

Figure S3

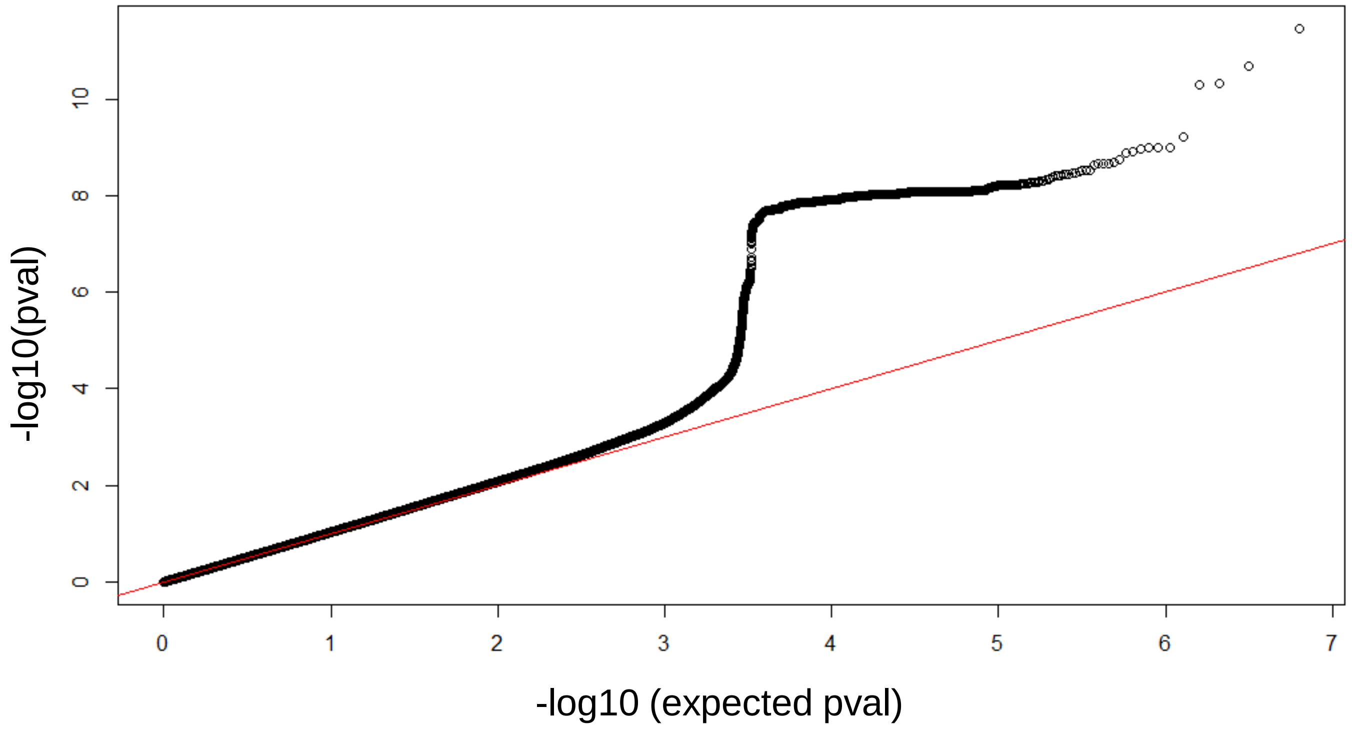

Figure S3 – *QQ plot of discovery phase*

Figure S4

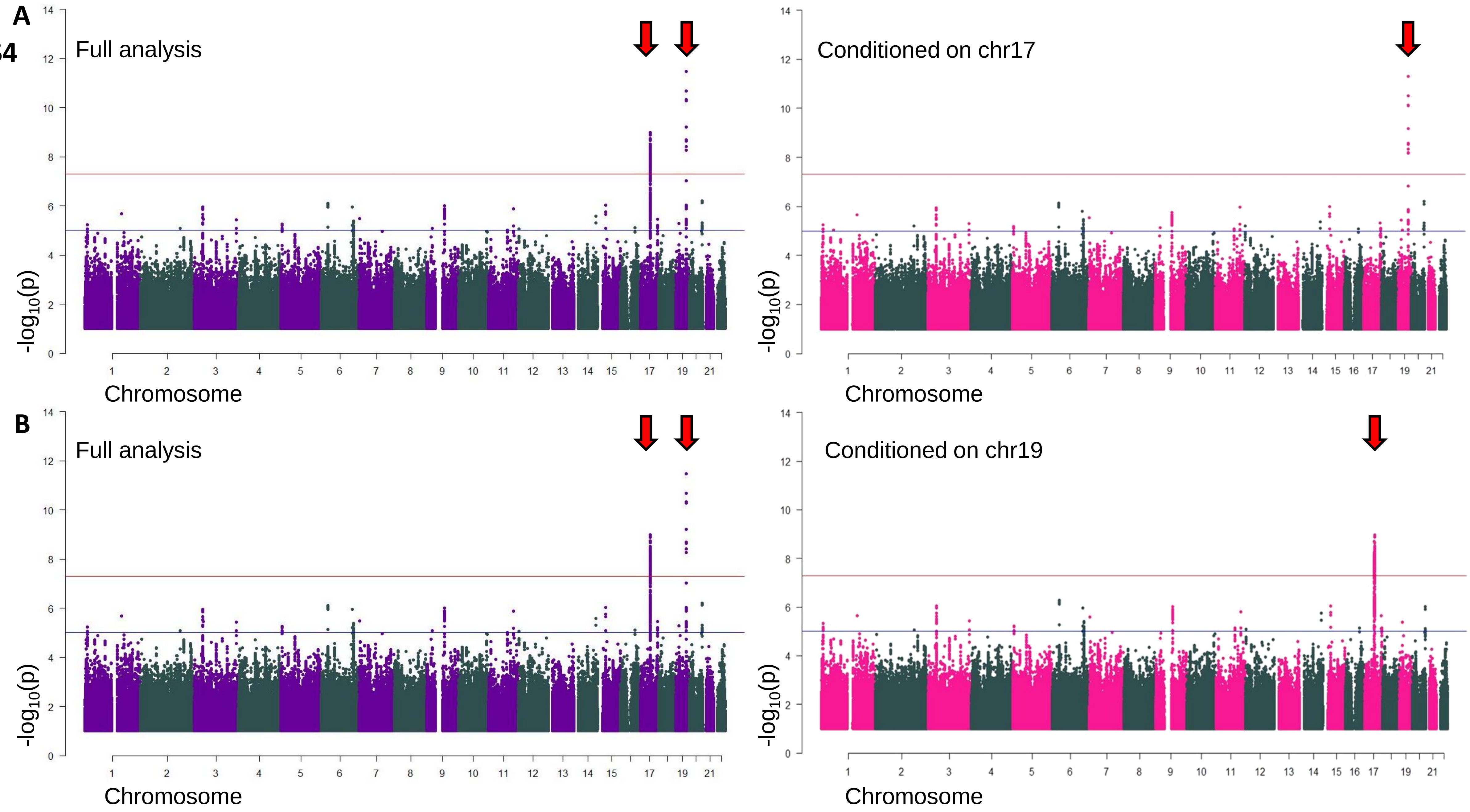

Figure S4 – *Manhattan plot for discovery phase. Arrows indicate genome-wide significant towers (on chromosomes 17 and 19) A. Full analysis followed by analysis after conditioning for the top SNP on chromosome 17; as highlighted, the signal on chromosome 17 disappears. B. Full analysis followed by analysis after conditioning for the top SNP on chromosome 19; as highlighted, the signal on chromosome 19 disappears. The plot is cut at -log*$_{10}$*(p) = 1.*

Figure S5

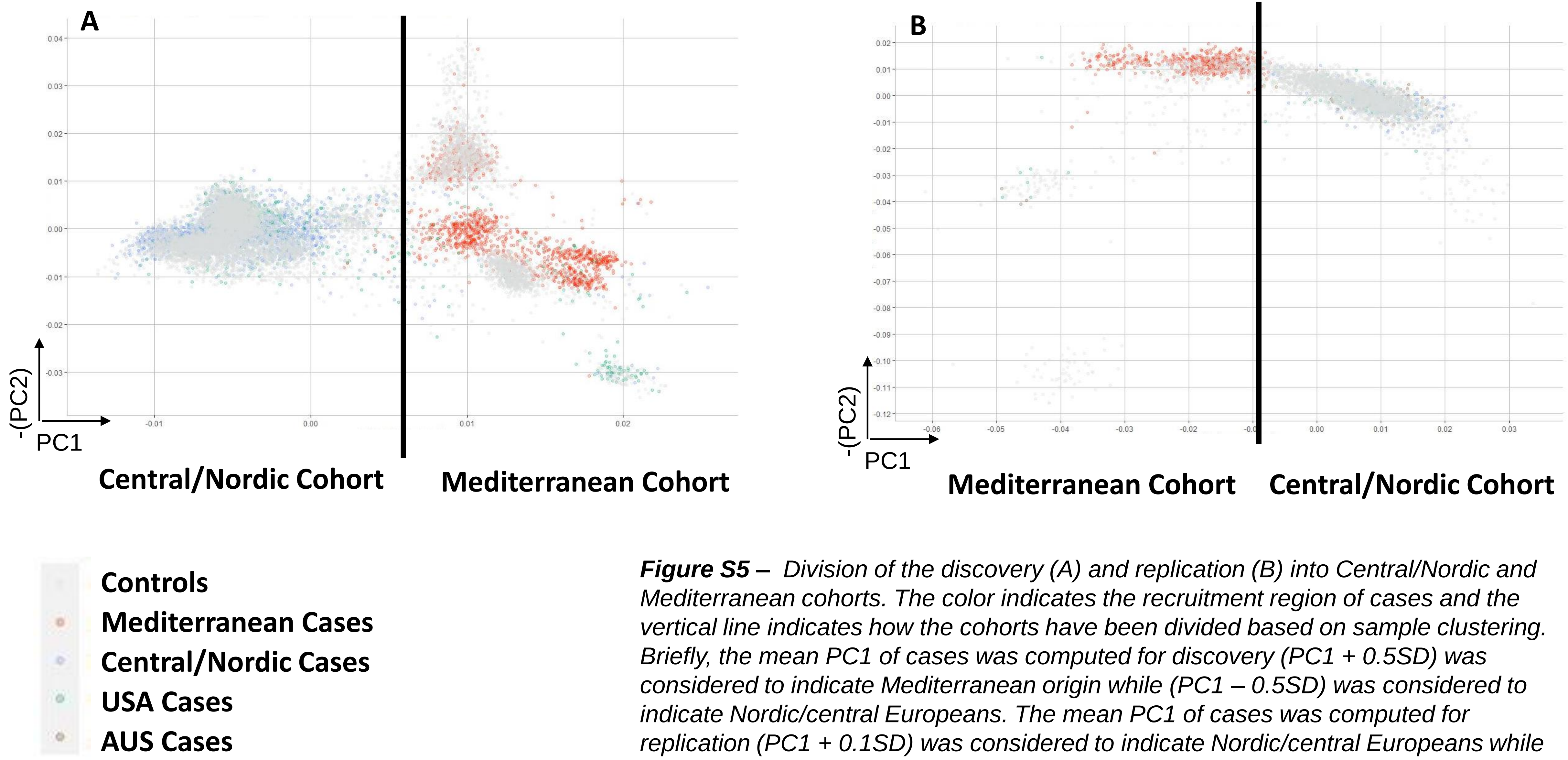

Figure S5 ***–*** *Division of the discovery (A) and replication (B) into Central/Nordic and Mediterranean cohorts. The color indicates the recruitment region of cases and the vertical line indicates how the cohorts have been divided based on sample clustering. Briefly, the mean PC1 of cases was computed for discovery (PC1 + 0.5SD) was considered to indicate Mediterranean origin while (PC1 – 0.5SD) was considered to indicate Nordic/central Europeans. The mean PC1 of cases was computed for replication (PC1 + 0.1SD) was considered to indicate Nordic/central Europeans while (PC1 – 0.1SD) was considered to indicate Mediterranean origin.*

Figure S6

A

Discovery Central/Nordic

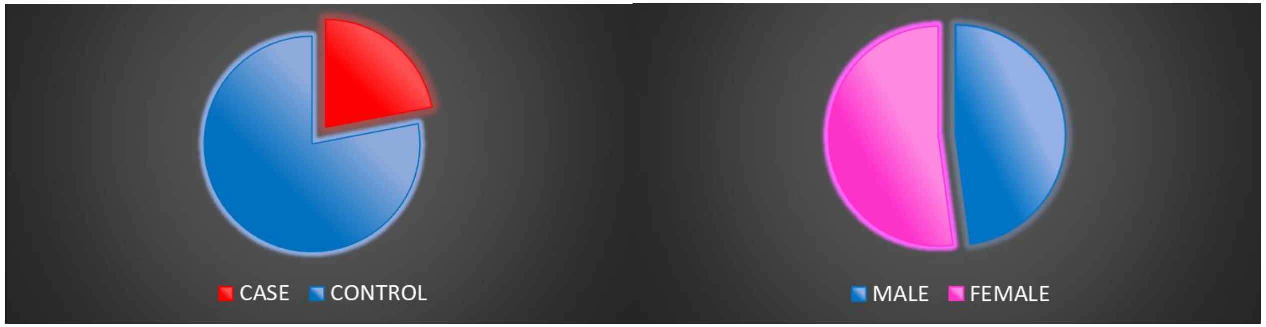

CASES: 2,359
CONTROLS: 8,371

MALES: 5,136
FEMALES: 5,594

Discovery Mediterranean

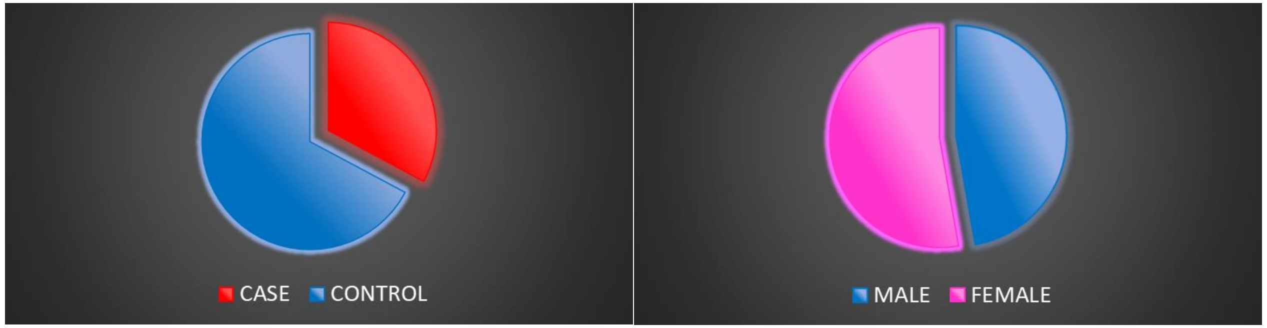

CASES: 1,397
CONTROLS: 2,862

MALES: 2,011
FEMALES: 2,248

B

Replication Central/Nordic

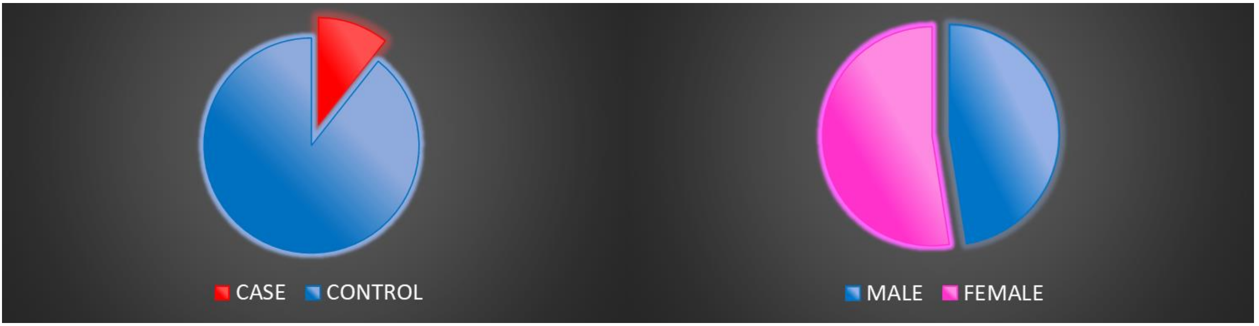

CASES: 405
CONTROLS: 3,400

MALES: 1,807
FEMALES: 1,998

Replication Mediterranean

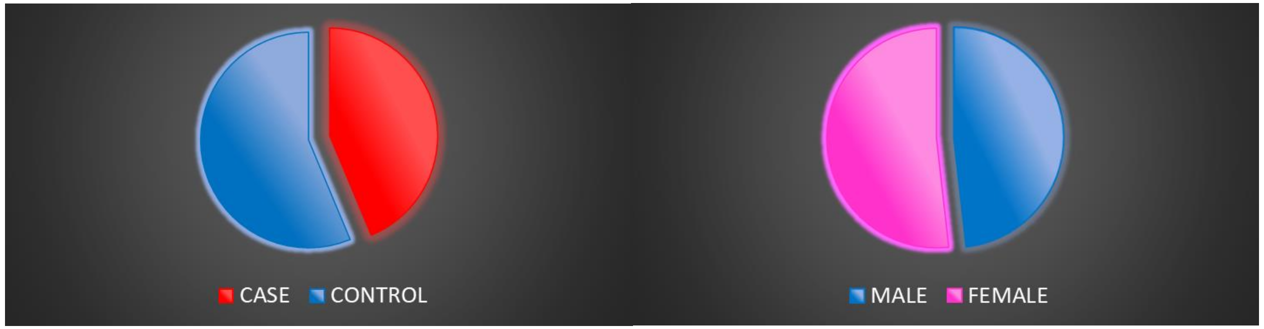

CASES: 524
CONTROLS: 675

MALES: 578
FEMALES: 621

Figure S6 – *features of the discovery (A) and replication (B) Central/Nordic and Mediterranean cohorts*

Figure S7

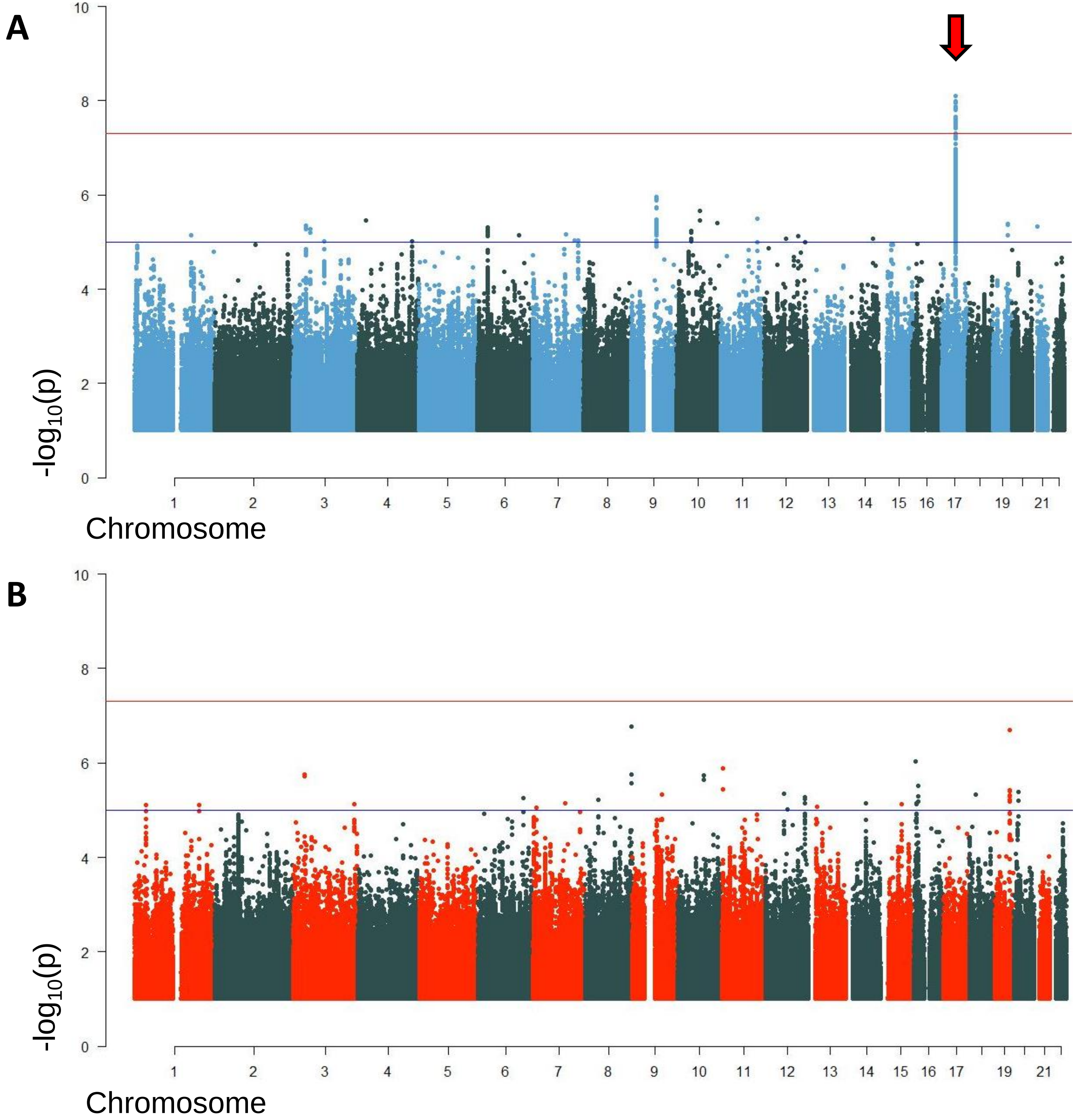

Figure S7 – Manhattan plot for discovery phase A. Central/Nordic and B. Mediterranean cohorts. Arrows indicate genome-wide significant tower (on chromosomes 17). The plot is cut at -log10(p) = 1.

Table S1

Collection Site	Institution	DISCOVERY			REPLICATION		
		Total	Sex		Total	Sex	
			Female	Male		Female	Male
Australia	University of Sydney - Neuroscience Research Australia	NA	NA	NA	93	59	34
Belgium	VIB-UAntwerp Center for Molecular Neurology	293	137	156	18	14	4
Canada	Vancouver General Hospital	80	44	36	10	6	4
	University of Toronto						
Denmark	Copenhagen University Hospital	6	5	1	NA	NA	NA
France	French Consortium	176	80	96	22	14	8
	Univeristy of Lille						
Germany	LMU Neurologische Klinik und Poliklinik	351	154	197	22	11	11
	Ludwig-Maximilians-University Munich						
	Technical University of Munich, School of Medicine						
	Universität des Saarlandes						
	University of Erlangen Nuremberg						
Italy	CNR Napoli	924	465	459	289	152	137
	IRCCS Istituto Centro San Giovanni di Dio Fatebenefratelli, Brescia						
	Istituto Neurologico C. Besta						
	Local Health Authority n.2 Marca Trevigiana, Treviso						
	Regional Neurogenetic Centre, ASPCZ, Lamezia Terme						
	University of Brescia						
	University of Florence						
	University of Milan						
	University of Salerno						
	University of Turin						
Netherlands	Erasmus Medical Center, Rotterdam	440	194	246	46	21	25
	VUMC, Amsterdam						
Norway	Institute of Clinical Medicine, University of Oslo	51	29	22	NA	NA	NA
	University Hospital of Trondheim						
Slovenia	Jožef Stefan Institute, Ljubljana	15	6	9	NA	NA	NA
Spain	Hospital Clínic of Barcelona	303	122	181	211	127	84
	Hospital de la Santa Creu i Sant Pau, Universitat Autònoma de Barcelona						
	Hospital Universitario Central de Asturias, Oviedo						
	Ace Alzheimer Center Barcelona						
	University Hospital Mutua de Terrassa, Terrassa, Barcelona						
Sweden	Karolinska Institutet	102	54	48	42	19	23
	Lund University						
	Skåne University Hospital, Malmö						
UK	Newcastle University	323	146	177	74	38	36
	Sheffield Institute for Translational Neuroscience (SITraN), University of Sheffield						
	University of Cambridge						
	University of Edinburgh						
	University of Manchester						
USA	Columbia University	692	325	367	102	48	54
	Mayo Clinic						
	UPENN						
	Northwestern, Chicago						
	UCSF						
	Washington University						
**Total**		**3756 (bvFTD: 2119; SD: 538; PNFA: 507; FTD-MND: 242; FTD-Unspecified: 350)**	**1761**	**1995**	**929 (bvFTD: 445; SD: 113; PNFA: 151; FTD-MND: 58; FTD-Unspecified: 162)**	**509**	**420**

Table S1 – Samples breakdown

Table S2 - *MAPT* and *APOE* loci significant markers

Table S3 - Association analysis using MAOS, A1 = minor allele, bold = risk allele.

Table S4 - Summary of suggestive markers taken forward for replication

Table S5

**#**	**ALPHA**	**=**	**0.05**								
**#**	**NUMBER_OF_TESTS**	**=**	**15258**								
**#**	**_SET1_**	**VARIABLE**	**=**	**GO_bp:go_negative_regulation_of_steroid_metabolic_process**	**(set)**						
**#**	**_SET1_**	**NGENES**	**=**	**13**							
**#**	**_SET1_**	**P-VALUE**	**=**	**9.19E-08**							
**_SET1_**	**GENE**	**CHR**	**START**	**STOP**	**NSNPS**	**NPARAM**	**N**	**ZSTAT**	**P**	**ZFITTED_BASE**	**ZRESID_BASE**
SET1	ENSG00000117707	1	214156524	214214595	1	1	14989	-0.26858	0.02975	-2.71E-13	-0.26858
SET1	ENSG00000125629	2	118846028	118868573	4	1	14989	-0.13193	0.019305	-2.71E-13	-0.13193
SET1	ENSG00000241635	2	234526291	234681956	63	6	14989	-0.23499	0.0025108	-2.71E-13	-0.23499
SET1	ENSG00000242366	2	234668894	234681945	4	2	14989	0.39278	0.0016643	-2.71E-13	0.39278
SET1	ENSG00000114650	3	47455203	47518616	2	1	14989	0.96345	0.00084899	-2.71E-13	0.96345
SET1	ENSG00000084093	4	57774075	57802010	11	5	14989	-0.040513	0.00083476	-2.71E-13	-0.040513
SET1	ENSG00000112175	6	55618443	55740362	6	3	14989	0.81191	0.00017715	-2.71E-13	0.81191
SET1	ENSG00000204740	10	19492779	20079330	182	15	14989	-0.47973	0.0018625	-2.71E-13	-0.47973
SET1	ENSG00000107566	10	101909851	101948091	3	2	14989	0.27814	0.0020591	-2.71E-13	0.27814
SET1	ENSG00000050165	11	11984653	12031316	18	4	14989	0.71168	0.0001573	-2.71E-13	0.71168
SET1	ENSG00000012504	12	100867486	100958191	8	4	14989	0.053432	0.0010974	-2.71E-13	0.053432
SET1	ENSG00000130203	19	45409011	45412650	4	2	14989	3.7285	1.35E-12	-2.70E-13	3.7285
SET1	ENSG00000124216	20	48599536	48605423	14	1	14989	0.30703	0.0075763	-2.71E-13	0.30703
**#**	**_SET2_**	**VARIABLE**	**=**	**GO_bp:go_negative_regulation_of_alcohol_biosynthetic_process**	**(set)**						
**#**	**_SET2_**	**NGENES**	**=**	**7**							
**#**	**_SET2_**	**P-VALUE**	**=**	**4.32E-08**							
**_SET2_**	**GENE**	**CHR**	**START**	**STOP**	**NSNPS**	**NPARAM**	**N**	**ZSTAT**	**P**	**ZFITTED_BASE**	**ZRESID_BASE**
SET2	ENSG00000115956	2	68592305	68624585	1	1	14989	-0.40249	0.04001	-2.71E-13	-0.40249
SET2	ENSG00000114650	3	47455203	47518616	2	1	14989	0.96345	0.00084899	-2.71E-13	0.96345
SET2	ENSG00000084093	4	57774075	57802010	11	5	14989	-0.040513	0.00083476	-2.71E-13	-0.040513
SET2	ENSG00000112175	6	55618443	55740362	6	3	14989	0.81191	0.00017715	-2.71E-13	0.81191
SET2	ENSG00000107566	10	101909851	101948091	3	2	14989	0.27814	0.0020591	-2.71E-13	0.27814
SET2	ENSG00000050165	11	11984653	12031316	18	4	14989	0.71168	0.0001573	-2.71E-13	0.71168
SET2	ENSG00000130203	19	45409011	45412650	4	2	14989	3.7285	1.35E-12	-2.70E-13	3.7285
**#**	**_SET3_**	**VARIABLE**	**=**	**GO_bp:go_negative_regulation_of_cholesterol_biosynthetic_process**	**(set)**						
**#**	**_SET3_**	**NGENES**	**=**	**3**							
**#**	**_SET3_**	**P-VALUE**	**=**	**9.81E-09**							
**_SET3_**	**GENE**	**CHR**	**START**	**STOP**	**NSNPS**	**NPARAM**	**N**	**ZSTAT**	**P**	**ZFITTED_BASE**	**ZRESID_BASE**
SET3	ENSG00000114650	3	47455203	47518616	2	1	14989	0.96345	0.00084899	-2.71E-13	0.96345
SET3	ENSG00000107566	10	101909851	101948091	3	2	14989	0.27814	0.0020591	-2.71E-13	0.27814
SET3	ENSG00000130203	19	45409011	45412650	4	2	14989	3.7285	1.35E-12	-2.70E-13	3.7285
**#**	**_SET4_**	**VARIABLE**	**=**	**GO_bp:go_regulation_of_fertilization**	**(set)**						
**#**	**_SET4_**	**NGENES**	**=**	**17**							
**#**	**_SET4_**	**P-VALUE**	**=**	**8.29E-07**							
**_SET4_**	**GENE**	**CHR**	**START**	**STOP**	**NSNPS**	**NPARAM**	**N**	**ZSTAT**	**P**	**ZFITTED_BASE**	**ZRESID_BASE**
SET4	ENSG00000085465	1	111956936	111970399	11	1	14989	-0.043923	0.015283	-2.71E-13	-0.043923
SET4	ENSG00000116996	1	238045705	238054094	15	2	14989	0.27718	0.0021094	-2.71E-13	0.27718
SET4	ENSG00000163803	2	28680012	28866654	7	4	14989	0.56205	0.00021321	-2.71E-13	0.56205
SET4	ENSG00000186792	3	50330262	50336899	1	1	14989	-0.16442	0.02283	-2.71E-13	-0.16442
SET4	ENSG00000145685	5	77781038	78065844	64	8	14989	-0.44883	0.0022813	-2.71E-13	-0.44883
SET4	ENSG00000214510	5	147647743	147665817	5	1	14989	1.15	0.00039321	-2.71E-13	1.15
SET4	ENSG00000145888	5	151202074	151304403	4	2	14989	0.24065	0.0022238	-2.71E-13	0.24065
SET4	ENSG00000124812	6	49801970	49844809	2	1	14989	0.54761	0.0030459	-2.71E-13	0.54761
SET4	ENSG00000188372	7	76026835	76071388	7	2	14989	-0.35493	0.015378	-2.71E-13	-0.35493
SET4	ENSG00000146707	7	76239303	76256578	6	2	14989	0.21662	0.0033286	-2.71E-13	0.21662
SET4	ENSG00000105792	7	89874488	89940377	15	1	14989	0.17805	0.012121	-2.71E-13	0.17805
SET4	ENSG00000214102	7	141408153	141431071	2	1	14989	0.84388	0.0011865	-2.71E-13	0.84388
SET4	ENSG00000086062	9	33104080	33167354	25	3	14989	-0.091904	0.0047878	-2.71E-13	-0.091904
SET4	ENSG00000182545	14	20973696	20979328	2	1	14989	1.3759	0.00023614	-2.71E-13	1.3759
SET4	ENSG00000255346	15	69222864	69355083	42	1	14989	0.19256	0.0129	-2.71E-13	0.19256
SET4	ENSG00000196557	16	1203241	1271771	3	2	14989	0.41432	0.0014887	-2.71E-13	0.41432
SET4	ENSG00000182621	20	8112824	8949003	191	18	14989	0.21758	7.23E-05	-2.71E-13	0.21758
**#**	**_SET5_**	**VARIABLE**	**=**	**GO_bp:go_mrna_pseudouridine_synthesis**	**(set)**						
**#**	**_SET5_**	**NGENES**	**=**	**2**							
**#**	**_SET5_**	**P-VALUE**	**=**	**1.77E-06**							
**_SET5_**	**GENE**	**CHR**	**START**	**STOP**	**NSNPS**	**NPARAM**	**N**	**ZSTAT**	**P**	**ZFITTED_BASE**	**ZRESID_BASE**
SET5	ENSG00000091127	7	105080108	105162714	1	1	14989	2.1102	1.12E-05	-2.70E-13	2.1102
SET5	ENSG00000110060	11	125763381	125773116	2	1	14989	0.42915	0.0064858	-2.71E-13	0.42915
**#**	**_SET6_**	**VARIABLE**	**=**	**GO_cc:go_intermediate_density_lipoprotein_particle**	**(set)**						
**#**	**_SET6_**	**NGENES**	**=**	**2**							
**#**	**_SET6_**	**P-VALUE**	**=**	**9.55E-07**							
**_SET6_**	**GENE**	**CHR**	**START**	**STOP**	**NSNPS**	**NPARAM**	**N**	**ZSTAT**	**P**	**ZFITTED_BASE**	**ZRESID_BASE**
SET6	ENSG00000084674	2	21224301	21266945	2	1	14989	0.39716	0.0071139	-2.71E-13	0.39716
SET6	ENSG00000130203	19	45409011	45412650	4	2	14989	3.7285	1.35E-12	-2.70E-13	3.7285
**#**	**_SET7_**	**VARIABLE**	**=**	**GO_mf:go_phosphatidylcholine_sterol_o_acyltransferase_activator_activity**	**(set)**						
**#**	**_SET7_**	**NGENES**	**=**	**3**							
**#**	**_SET7_**	**P-VALUE**	**=**	**1.82E-06**							
**_SET7_**	**GENE**	**CHR**	**START**	**STOP**	**NSNPS**	**NPARAM**	**N**	**ZSTAT**	**P**	**ZFITTED_BASE**	**ZRESID_BASE**
SET7	ENSG00000110244	11	116691419	116694022	1	1	14989	0.17051	0.01031	-2.71E-13	0.17051
SET7	ENSG00000130203	19	45409011	45412650	4	2	14989	3.7285	1.35E-12	-2.70E-13	3.7285
SET7	ENSG00000130208	19	45417504	45422606	2	1	14989	2.3572	4.46E-06	-2.70E-13	2.3572

Table S5 – Functional Analysis

Table S6

QTL	chr	Gene Symbol	SNP Id	P-Value	Tissue
eQTL	17	*KANSL1-AS1*	rs199443	1.30E-43	Brain - Cortex
		RP11-259G18.3	rs199443	2.80E-39	Brain - Cortex
		LRRC37A2	rs199443	4.20E-37	Brain - Cortex
		MAPK8IP1P1	rs199443	7.00E-29	Brain - Cortex
		ARL17A	rs199443	2.70E-25	Brain - Cortex
		RP11-259G18.1	rs199443	1.80E-16	Brain - Cortex
		LRRC37A	rs199443	1.40E-12	Brain - Cortex
		CRHR1	rs199443	1.80E-04	Brain - Cortex
		KANSL1-AS1	rs199443	4.10E-37	Brain - Frontal Cortex (BA9)
		LRRC37A2	rs199443	6.90E-33	Brain - Frontal Cortex (BA9)
		RP11-259G18.3	rs199443	2.80E-31	Brain - Frontal Cortex (BA9)
		MAPK8IP1P1	rs199443	4.00E-25	Brain - Frontal Cortex (BA9)
		ARL17A	rs199443	9.10E-24	Brain - Frontal Cortex (BA9)
		RP11-259G18.1	rs199443	2.40E-12	Brain - Frontal Cortex (BA9)
		LRRC37A	rs199443	7.30E-11	Brain - Frontal Cortex (BA9)
		SPPL2C	rs199443	1.80E-05	Brain - Frontal Cortex (BA9)
	3	*RPSA*	rs13081054	1.30E-05	Brain - Cerebellar Hemisphere
		RPSA	rs13081054	2.40E-06	Brain - Cerebellum
sQTL	17	*KANSL1*	rs199443	3.10E-50	Brain - Cortex
		CRHR1	rs199443	2.90E-17	Brain - Cortex
		MAPT	rs199443	2.00E-06	Brain - Cortex
		KANSL1	rs199443	5.20E-43	Brain - Frontal Cortex (BA9)
		CRHR1	rs199443	2.10E-16	Brain - Frontal Cortex (BA9)
		MAPT	rs199443	5.80E-09	Brain - Frontal Cortex (BA9)
	3	*RPSA*	rs13081054	2.70E-06	Brain - Cortex

Table S6 - eQTL/sQTL from gTEX

Table S7

	H2 (SE)	Lambda_GC	Ratio (SE)	Intercept	H2 (se)	Lambda_GC
	**Observed scale**					
FTD	0.067 (0.03)	1.03	0.42(0.18)	1.01	0.118(0.02)	1.03
FTD_noAPOE	0.069(0.02)	1.03	0.4 (0.17)	1.01	0.117(0.02)	1.03
	**Liability scale**					
FTD	0.028(0.013)	1.03	0.42(0.18)	1.01	0.049 (0.0085)	1.03
FTD_noAPOE	0.029 (0.012)	1.03	0.4(0.17)	1.01	0.049 (0.008)	1.03

	r_g (SE)	P	N shared SNPs
FTD vs AD	0.55 (0.23)	0.02	1,166,449
FTD vs ADRD	0.28 (0.15)	0.06	1,080,004
FTD vs LBD	0.91 (0.46)	0.05	997,061
FTD vs ALS	0.71 (0.3)	0.02	1,160,125
FTD vs PD	0.32 (0.14)	0.03	1,091,414

Table S7 - Heritability estimation with LDSC regression

Table S8

Pheno	Marker	chr	Gene	Original work (p-value; OR)	Ref	Current study (Discovery) (p-value; OR)	LD with top SNP in current study	D'; R2
FTLD-TDP	rs1990622	7	*TMEM106B*	$1.08\text{x}10^{-11}$; 0.61	PMID: 20154673	$2.3\text{x}10^{-1}$; 0.96		
FTD-GRN	rs36196656	8	*GFRA2*	$1.58\text{x}10^{-8}$; 1.49	PMID: 29724592	$7.76\text{x}10^{-1}$; 1.01		
FTD-Clinical	rs9268877	6	*HLA-DR*	$1.05\text{x}10^{-8}$; 1.2	PMID: 24943344	$9.6\text{x}10^{-7}$; 1.16		
	rs302668	11	*RAB38/CTSC*	2.44×10^{-7}; 0.81		$9.86\text{x}10^{-2}$; 0.95		
ALS	rs3849943	9	*C9orf72*	3.8×10^{-30}; 0.84	PMID: 29566793	$5.5\text{x}10^{-1}$; 1.02		
	rs12973192*	19	*UNC13A*	3.9×10^{-15}; 0.89		$1.07\text{x}10^{-3}$; 1.1		
	rs142321490**	12	*KIF5A*	6.1×10^{-10}; 1.37		NA; NA		
	rs631312	3	*MOBP*	5.2×10^{-11}; 1.1	PMID: 34873335	$1.6\text{x}10^{-4}$; 1.13	Complete - non perfect	1; 0.3
AD	rs4420638	19	*APOC1*	$1.28\text{x}10^{-253}$; 3.95	PMID: 21460841	$9.6\text{x}10^{-8}$; 1.2	Almost complete - non perfect	0.77; 0.5
	rs439401		*APOE*	$1.06\text{x}10^{-49}$; 1.5		$8.1\text{x}10^{-1}$; 1.01	No LD	0.58; 0.047
	rs7412			$5.9\text{x}10^{-12}$; 1.5	PMID: 34099642	$1.9\text{x}10^{-4}$; 1.2	Complete - non perfect	1; 0.02
	rs6656401	1	*CR1*	5.7×10^{-24}; 1.18	PMID: 24162737	$5.4\text{x}10^{-2}$; 1.07		
	rs679515			7.2×10^{-46}; 1.13	PMID: 35379992	$1.1\text{x}10^{-1}$; 1.06		
	rs6733839	2	*BIN1*	6.9×10^{-44}; 1.22	PMID: 24162737	$2.96\text{x}10^{-2}$; 1.07		
	rs9331896	8	*CLU*	2.8×10^{-25}; 0.86		$9.3\text{x}10^{-1}$; 0.997		
	rs11787077			1.7×10^{-44}; 0.91	PMID: 35379992	$8.3\text{x}10^{-1}$, 0.99		
	rs3851179	11	*PICALM*	3×10^{-48}; 0.9		$6.5\text{x}10^{-3}$, 0.92		
	rs6605556***	6	*HLA-DR*	7.1×10^{-20}; 0.91		$2.8\text{x}10^{-2}$, 0.91		
	rs199515****	17	*MAPT*	9.3×10^{-13}; 1.06		$1.1\text{x}10^{-9}$, 1.3	Complete - perfect	1; 0.97
	rs1009966$	3	*MOBP*	$3.4\text{x}10^{-1}$; 1.01	PMID: 31417202	(joint analysis p-value current manuscript) $2.41\text{x}10^{-8}$, 1.16		
				$7.5\text{x}10^{-1}$; 1	PMID: 35379992			
PD	rs17649553	17	*MAPT*	1.26×10^{-68}; 0.78	PMID: 28892059	$1.97\text{x}10^{-8}$; 0.82	Complete - perfect	1; 0.91
PSP	rs1411478	1	*STX6*	2.3×10^{-10}; 0.79	PMID: 21685912	$7.82\text{x}10^{-3}$; 1.08		
	rs7571971	2	*EIF2AK3*	3.2×10^{-13}; 0.75		$6.6\text{x}10^{-1}$; 1.01		
	rs1768208	3	*MOBP*	1.0×10^{-16}; 0.72		$5.13\text{x}10^{-4}$; 1.12	Complete - non perfect	1; 0.31
	rs8070723	17	*MAPT*	1.5×10^{-116}; 5.46		$9.21\text{x}10^{-9}$; 0.81	Complete - perfect	1; 0.94
	rs11568563	12	*SLCO1A2*	5.26E-10; 0.67	PMID: 29986742	$4.66\text{x}10^{-3}$; 1.19		
	rs6687758	1	*DUSP10*	1.14×10^{-8}; 0.80		$7.3\text{x}10^{-1}$; 1.01		
CBD	rs393152	17	*MAPT*	1.42×10^{-12}; 3.70	PMID: 26077951	$2.06\text{x}10^{-8}$; 0.82	Complete - perfect	1; 0.94
	rs643472	8	*lnc-KIF13B-1*	3.41×10^{-8}; 1.82		$8.5\text{x}10^{-1}$; 0.993		

* = PROXY rs12608932 (D'=1; R2=0.98)
** = PROXY not available
*** = PROXY rs9272461 (D'=1; R2=1)
**** = PROXY rs199528 (D'=1; R2=1)
$ = *MOBP* hit reported in the current manuscript checked in 2 AD-GWAS datasets

Table S8 - Assessment of significant markers from other neurodegenerative disease or studies vs. the current study

Note S1

Characterisation of *APOE Ɛ4* in the cohort

We first analysed the *APOE Ɛ4* alleles by extracting the allele frequencies for rs429358 (C:T) and rs7412 (T:C) in the original discovery and replication cohorts, and compared cases vs. controls (**Table**): allele frequency differences were statistically significant in both the discovery and replication cohorts ($p_{discovery}$=3.6x10^{-14}; $p_{replication}$=5.5x10^{-4}) suggesting that the cases in our cohorts are enriched in Ɛ4 alleles.

Population	CASES count	CASES freq	CONTROLS count	CONTROLS freq
**Discovery**	1179	0.157	2759	0.123
**Replication**	301	0.162	1068	0.131
**1000 Genomes EUR**			114	0.141
**Discovery Mediterranean**	417	0.149	500	0.087
**Replication Mediterranean**	159	0.152	130	0.096
**1000 Genomes IBR+TSI**			49	0.114
**New cohort Italians**			135	0.093
**Discovery Central/Nordic**	762	0.162	2259	0.135
**Replication Central/Nordic**	142	0.175	938	0.138
**1000 Genomes CEU+GBR**			65	0.171
**New cohort Europeans**			100	0.145

We then did the same for the two sub-cohorts (Central/Nordic cases vs. controls and Mediterranean cases vs. controls): all comparisons indicated that allele frequency differences were statistically significant ($p_{Central/Nordic}$ =3.7x10^{-6}, $p_{Mediterranean}$=2.2x10^{-16}), confirming a higher frequency of Ɛ4 alleles in FTD cases regardless from ancestry.

Finally, we compared the *APOE* Ɛ4 allele frequencies between our Central/Nordic and Mediterranean European controls: allele frequency differences were statistically significant ($p_{Discovery-Controls}$=2.2x10^{-16}), indicating that Central/Nordic European controls carried a remarkably higher frequency of Ɛ4 alleles compared to Mediterranean European controls. We confirmed this trend in further independent cohorts, *i.e.* Central/Nordic vs. Mediterranean Europeans for (1) the populations from 1000G (p_{1000G}=2.7x10^{-2}) and (2) an additional cohort of 731 Italian and 347 European controls ($p_{NewCohort}$=3.4x10^{-4}).

All this taken together suggests that: (1) FTD cases carry a slightly increased frequency of Ɛ4 alleles compared to controls, regardless of ancestry and (2) Central/Nordic Europeans (control population) carry a higher frequency of Ɛ4 alleles compared to Mediterranean Europeans (control population) regardless of disease status (**Figure**).

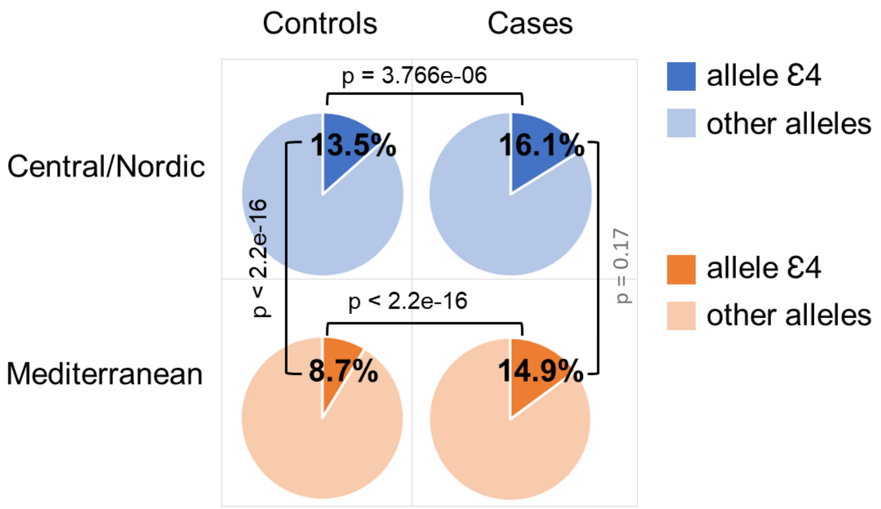

Figure– *APOE Ɛ4 allele frequencies in the Central/Nordic and Mediterranean discovery cohorts. Uncorrected p-values calculated via χ^2 are reported.*

Note S2 – list of PIs

Pis Name
Diego Albani
Giuliano Binetti and/or Roberta Ghidoni
Daniel Blackburn
Barbara Borroni
Carlos Cruchaga
Adrian Danek
Janine Diehl-Schmid
Carol Dobson-Stone
Oriol Dols-Icardo
Daniela Galimberti
Maurizio Gallucci
Caroline Graff
Jordan Grafman
Edward Huey
Isabelle Le Ber
Ian Mackenzie
Raffaele Maletta
Manuel Menendez-Gonzalez
Christopher Morris
Benedetta Nacmias
Jørgen Nielsen
Valeria Novelli
Suvankar Pal
Florence Pasquier
Pau Pastor
Robert Perneczky
Yolande Pijnenburg
Rosa Rademakers
Innocenzo Rainero
Matthias Riemenschneider
Ekaterina Rogaeva
Boris Rogelj
Giacomina Rossi
James Rowe
Agustín Ruiz
Raquel Sanchez-Valle
Sigrid Sando
Jennifer Saxon and/or Jennifer Thompson
Johannes Schlachetzki
Vivianna Van Deerlin
Julie van der Zee
John Van Swieten
Emilia Vitale
Maria Landqvist-Waldo
Jennifer Yokoyama

Additional acknowledgement and funding

JH – This work was supported by the UK Dementia Research Institute which receives its funding from UK DRI Ltd (UKDRI-1009) funded by the UK Medical Research Council, Alzheimer's Society and Alzheimer's Research UK. JH received funding from the Dolby Family Fund; VA – is supported by the Fondo de Investigaciones Sanitarias' Spanish government ICIII FIS-FEDER grants (ID grant: PI21/0467); OAA – Research Council of Norway (223273), Norwegian Health Association (#22731); RG & LB & GB – Italian Ministry of Health, Ricerca Corrente; DJB – Sheffield BRC; MB & MM & AR – The Genome Research @ Ace Alzheimer Center Barcelona project (GR@ACE) is supported by Grifols SA, Fundación bancaria 'La Caixa', Ace Alzheimer Center Barcelona and CIBERNED. Ace Alzheimer Center Barcelona is one of the participating centers of the Dementia Genetics Spanish Consortium (DEGESCO). Acknowledge the support of the Spanish Ministry of Science and Innovation, Proyectos de Generación de Conocimiento grants PID2021-122473OA-I00, PID2021-123462OB-I00 and PID2019-106625RB-I00. ISCIII, Acción Estratégica en Salud, integrated in the Spanish National R+D+I Plan and financed by ISCIII Subdirección General de Evaluación and the Fondo Europeo de Desarrollo Regional (FEDER "Una manera de hacer Europa") grants PI13/02434, PI16/01861, PI17/01474, PI19/00335, PI19/01240, PI19/01301, PI22/01403, PI22/00258 and the ISCIII national grant PMP22/00022, funded by the European Union (NextGenerationEU). The support of CIBERNED (ISCIII) under the grants CB06/05/2004 and CB18/05/00010. The support from the ADAPTED and MOPEAD projects, European Union/EFPIA Innovative Medicines Initiative Joint (grant numbers 115975 and 115985, respectively); from PREADAPT project, Joint Program for Neurodegenerative Diseases (JPND) grant Nº AC19/00097; from HARPONE project, Agency for Innovation and Entrepreneurship (VLAIO) grant Nº PR067/21 and Janssen. DESCARTES project is funded by German Research Foundation (DFG); BFB – P50 AG016574 and P30 AG062677; SB-E – recipient of the Research Contract FBBVA-Rodés-Baselga; BC – Alzheimer's Society for its support and funding (grant 447); CC – was supported by grants from the National Institutes of Health, R01AG044546 (CC), P01AG003991(CC, JCM), RF1AG053303 (CC), RF1AG058501 (CC), U01AG058922 (CC), and the Chuck Zuckerberg Initiative (CZI), and the Alzheimer's Association Zenith Fellows Award (ZEN-22-848604, awarded to CC). This work was supported by access to equipment made possible by the Hope Center for Neurological Disorders, the Neurogenomics and Informatics Center (NGI: https://neurogenomics.wustl.edu/)and the Departments of Neurology and Psychiatry at Washington University School of Medicine; DWD – P50NS072187, P50AG016574, State of Florida Alzheimer Disease Initiative, and CurePSP Inc; CD-S – is supported by National Health and Medical Research Council of Australia (NHMRC) Boosting Dementia Research Leadership Fellowship 1138223 and the University of Sydney; OD-I – is a recipient of a grant by The Association for Frontotemporal Degeneration (Clinical Research Postdoctoral Fellowship); CG – is supported by grants from Swedish FTD Initiative-The Schörling Foundation, JPND Prefrontals Research Council (VR) 529-2014-7504, Research Council (VR) 2015-02926, Research Council (VR) 2018-02754, Brain Foundation, Alzheimer Foundation, Region Stockholm (ALF-Project) and Demensfonden, Sweden; NRG-R – P50 AG016574 and P30 AG062677; KAJ – R01 AG037491; DSK – P50 AG016574 and P30 AG062677; MK – UCL Genomics core facility; ILB – French clinical and genetic research network on FTD/FTD-ALS; GL – supported by UK DRI; IRM – Research funding from CIHR and NIH; GKM – UCL Genomics core facility; MM-G – is supported by the Fondo de Investigaciones Sanitarias' Spanish government ICIII FIS-FEDER grants (ID grants: PI21/0467); BLM – P30AG062422, P01AG019724; CMM – Newcastle Brain Tissue Resource, which is funded in part by a grant from the UK Medical Research Council, by NIHR Biomedical Research Centre Newcastle awarded to the Newcastle upon Tyne NHS Foundation Trust and Newcastle University, and as part of the Brains for Dementia Research Programme jointly funded by Alzheimer's Research UK and Alzheimer's Society; HRM – PSP Association; BN – RICATENEO2023; MAN – supported in part by the Intramural Research Program of the NIH, National Institute on Aging (NIA), National Institutes of Health, Department of Health and Human Services; project number ZIAAG000534, as

well as the National Institute of Neurological Disorders and Stroke. This work utilized the computational resources of the NIH HPC Biowulf cluster (http://hpc.nih.gov); RP – is supported by Davos Alzheimer's Collaborative, the VERUM Foundation, the Robert-Vogel-Foundation, the German Center for Neurodegenerative Diseases (DZNE), the National Institute for Health and Care Research (NIHR) Sheffield Biomedical Research Centre (NIHR203321), the University of Cambridge – Ludwig-Maximilians-University Munich Strategic Partnership within the framework of the German Excellence Initiative and Excellence Strategy and the European Commission under the Innovative Health Initiative program (project 101132356); RCP – P50 AG016574 and P30 AG062677; OP – is supported by a National Health and Medical Research Council of Australia Leadership Fellowship (GNT2008020); RR – P50 AG016574, R01 NS080882, R01 NS065782, P50 NS72187, and the Consortium for Frontotemporal Dementia; IR and ER – Grant of the Italian Ministry of the University (MUR), project "Dipartimenti di Eccellenza 2023–2027" to the Department of Neuroscience "Rita Levi Montalcini", University of Torino, Italy. ER – the Canadian Consortium on Neurodegeneration in Aging; HR – AG032306 and K24 AG045333; JBR – was supported by the NIHR Cambridge Biomedical Research Centre (NIHR203312). The views expressed are those of the author(s) and not necessarily those of the NIHR or the Department of Health and Social Care' Wellcome Trust (220258; 103838). For the purpose of open access, the authors have applied a CC BY public copyright licence to any Author Accepted Manuscript version arising from this submission; NIHR Cambridge Biomedical Research Centre (NIHR203312); the views expressed are those of the author and not necessarily those of the NIHR or the Department of Health and Social Care. Cambridge Centre for Parkinson-plus; SWS – was supported in part by the intramural research program of the National Institute of Neurological Disorders and Stroke, National Institutes of Health (program #: ZIANS003154); WWS – NIH AG023501, AG019724, Consortium for Frontotemporal Dementia Research; MS – is supported by the Italian Ministry of Health, grant GR-2019-12369100; ABS – was supported in part by the Intramural Research Program of the NIH, National Institute on Aging (NIA), National Institutes of Health, Department of Health and Human Services; project number ZIAAG000534, as well as the National Institute of Neurological Disorders and Stroke. This work utilized the computational resources of the NIH HPC Biowulf cluster (http://hpc.nih.gov); SS – RICATENEO2023; PStG-H – the Canadian Consortium on Neurodegeneration in Aging; MMXT – is employed by Oslo University Hospital. She has received grant support from Parkinson's UK, the Michael J Fox Foundation, and South-Eastern Norway Regional Health Authority (Helse Sør-Øst); CVB – was in part funded by the MetLife Foundation Award for Medical Research, the Methusalem Excellence Program of the Flemish Government and the University of Antwerp Research Fund, Belgium; VMVD – P01 AG-066597, P30-AG-072979; JvdZ – was in part funded by the MetLife Foundation Award for Medical Research, the Methusalem Excellence Program of the Flemish Government and the University of Antwerp Research Fund, Belgium; KJ – NIA grant R01-AG037491; SJVdL – The Alzheimer Center Amsterdam is supported by Stichting Alzheimer Nederland and Stichting VUmc fonds. The clinical database structure was developed with funding from Stichting Dioraphte. Genotyping of the Dutch case-control samples was performed in the context of EADB (European Alzheimer DNA biobank) funded by the JPco-fuND FP-829-029 (ZonMW projectnumber 733051061); is recipient of funding from ZonMW (#733050512), Health~Holland, Topsector Life Sciences & Health (PPP-allowance; #LSHM20106). Stichting Dioraphte, the Edwin Bouw Fonds and Stichting VUmc fonds. SvdL is recipients of ABOARD, which is a public-private partnership receiving funding from ZonMW (#73305095007) and Health~Holland, Topsector Life Sciences & Health (PPP-allowance; #LSHM20106). The Alzheimer Center Amsterdam is supported by Stichting Alzheimer Nederland and Stichting VUmc fonds. The clinical database structure was developed with funding from Stichting Dioraphte. Genotyping of the Dutch case-control samples was performed in the context of EADB (European Alzheimer DNA biobank) funded by the JPco-fuND FP-829-029 (ZonMW projectnumber 733051061); S.L. is recipient of funding from ZonMW (#733050512), Health~Holland, Topsector Life Sciences & Health (PPP-allowance; #LSHM20106). Stichting Dioraphte, the Edwin Bouw Fonds and Stichting VUmc

fonds; WF, SvdL, HHolstege, CT and PhS are recipients of ABOARD, which is a public-private partnership receiving funding from ZonMW (#73305095007) and Health~Holland, Topsector Life Sciences & Health (PPP-allowance; #LSHM20106). More than 30 partners participate in ABOARD (www.aboard-project.nl). ABOARD also receives funding from de Hersenstichting, Edwin Bouw Fonds and Gieskes-Strijbisfonds; LMR – was funded by the NIH National Institute on Aging (NIA) R21 AG072390 and by the Memorabel fellowship (ZonMW projectnumber: 10510022110012); YALP – is a recipient of funding from NWO (KICH1.GZ02.20.004), Stichting ZABAWAS, Hersenstichting and Health~Holland. IdR is supported by a national grant from the Instituto de Salud Carlos III (ISCIII) FI20/00215.
