# Supplementary material for: Genome-wide analyses reveal a potential role for the MAPT, MOBP, and APOE loci in sporadic frontotemporal dementia
Source: Am J Hum Genet. 2024 Jun 17;111(7):1316–29. doi: 10.1016/j.ajhg.2024.05.017 (PMC11267522; doi:10.1016/j.ajhg.2024.05.017)
Supplement: Document S2. Article plus supplemental information [file mmc5.pdf]

Genome-wide analyses reveal a potential role for the *MAPT*, *MOBP*, and *APOE* loci in sporadic frontotemporal dementia

Authors

Claudia Manzoni, Demis A. Kia, Raffaele Ferrari, ..., Andrew B. Singleton, John Hardy, Valentina Escott-Price

Correspondence

c.manzoni@ucl.ac.uk (C.M.),
escottpricev@cardiff.ac.uk (V.E.-P.)

We identified associations at the *MAPT*, *APOE*, and *MOBP* loci with FTD, suggesting potential common genetic denominators across multiple neurological conditions (i.e., FTD, PSP, ALS, AD, PD, and CBD). These findings will aid in the further characterization of the common and disease-specific pathogenesis of, and interventions for, these disorders.

Manzoni et al., 2024, The American Journal of Human Genetics *111*, 1316–1329

July 11, 2024 © 2024 The Author(s).

https://doi.org/10.1016/j.ajhg.2024.05.017

ARTICLE

Genome-wide analyses reveal a potential role for the *MAPT*, *MOBP*, and *APOE* loci in sporadic frontotemporal dementia

Claudia Manzoni,[1,121,*] Demis A. Kia,[2,121] Raffaele Ferrari,[2,121] Ganna Leonenko,[3] Beatrice Costa,[2] Valentina Saba,[4] Edwin Jabbari,[2] Manuela MX. Tan,[2,5] Diego Albani,[6] Victoria Alvarez,[7,8] Ignacio Alvarez,[9,10] Ole A. Andreassen,[11,12] Antonella Angiolillo,[13] Andrea Arighi,[14] Matt Baker,[15] Luisa Benussi,[16,122] Valentina Bessi,[17] Giuliano Binetti,[18] Daniel J. Blackburn,[19] Merce Boada,[20,21] Bradley F. Boeve,[22] Sergi Borrego-Ecija,[23] Barbara Borroni,[24] Geir Bråthen,[25,26] William S. Brooks,[27] Amalia C. Bruni,[28] Paola Caroppo,[29] Sara Bandres-Ciga,[30] Jordi Clarimon,[31] Rosanna Colao,[28] Carlos Cruchaga,[32,33] Adrian Danek,[34] Sterre CM. de Boer,[35,36,37] Itziar de Rojas,[20,21]

(Author list continued on next page)

Summary

Frontotemporal dementia (FTD) is the second most common cause of early-onset dementia after Alzheimer disease (AD). Efforts in the field mainly focus on familial forms of disease (fFTDs), while studies of the genetic etiology of sporadic FTD (sFTD) have been less common. In the current work, we analyzed 4,685 sFTD cases and 15,308 controls looking for common genetic determinants for sFTD. We found a cluster of variants at the *MAPT* (rs199443; $p = 2.5 \times 10^{-12}$, OR = 1.27) and *APOE* (rs6857; $p = 1.31 \times 10^{-12}$, OR = 1.27) loci and a candidate locus on chromosome 3 (rs1009966; $p = 2.41 \times 10^{-8}$, OR = 1.16) in the intergenic region between *RPSA* and *MOBP*, contributing to increased risk for sFTD through effects on expression and/or splicing in brain cortex of functionally relevant in-*cis* genes at the *MAPT* and *RPSA-MOBP* loci. The association with the *MAPT* (H1c clade) and *RPSA-MOBP* loci may suggest common genetic pleiotropy across FTD and progressive supranuclear palsy (PSP) (*MAPT* and *RPSA-MOBP* loci) and across FTD, AD, Parkinson disease (PD), and cortico-basal degeneration (CBD) (*MAPT* locus). Our data also suggest population specificity of the risk signals, with *MAPT* and *APOE* loci associations mainly driven by Central/Nordic and Mediterranean Europeans, respectively. This study lays the foundations for future work aimed at further characterizing population-specific features of potential FTD-discriminant *APOE* haplotype(s) and the functional involvement and contribution of the *MAPT H1c* haplotype and *RPSA-MOBP* loci to pathogenesis of sporadic forms of FTD in brain cortex.

Introduction

The set of neuropathologies known as frontotemporal lobar degeneration (FTLD; MIM: 607485) make up the second most common cause of early-onset dementia after Alzheimer disease (AD; MIM: 104310).[1,2] The most common presentation is frontotemporal dementia (FTD; MIM: 600274), which itself comprises heterogeneous clinical, pathological, and genetic features.

Clinically, the two major syndromes are the behavioral (bvFTD) and the language variants (primary progressive aphasias [PPAs]).[3,4] The latter are further subdivided into

[1]UCL School of Pharmacy, London, UK; [2]Department of Clinical and Movement Neurosciences, UCL Queen Square Institute of Neurology, London, UK; [3]Division of Psychological Medicine and Clinical Neurosciences, UK Dementia Research Institute, School of Medicine, Cardiff University, Cardiff, UK; [4]Medical and Genomic Statistics Unit, Department of Brain and Behavioral Sciences, University of Pavia, Pavia, Italy; [5]Department of Neurology, Oslo University Hospital, Oslo, Norway; [6]Istituto di Ricerche Farmacologiche Mario Negri IRCCS, Milano, Italy; [7]Hospital Universitario Central de Asturias, Oviedo, Spain; [8]Instituto de Investigación Sanitaria del Principado de Asturias, Oviedo, Spain; [9]Memory Disorders Unit, Department of Neurology, Hospital Universitari Mutua de Terrassa, Terrassa, Barcelona, Spain; [10]Fundació Docència i Recerca MútuaTerrassa, Terrassa, Barcelona, Spain; [11]NORMENT Centre, Institute of Clinical Medicine, University of Oslo, Oslo, Norway; [12]Division of Mental Health and Addiction, Oslo University Hospital, Oslo, Norway; [13]Centre for Research and Training in Medicine of Aging, Department of Medicine and Health Science "V. Tiberio," University of Molise, Campobasso, Italy; [14]Fondazione IRCCS Ca' Granda, Ospedale Maggiore Policlinico, Milan, Italy; [15]Department of Neuroscience, Mayo Clinic Jacksonville, Jacksonville, FL, USA; [16]Molecular Markers Laboratory, IRCCS Istituto Centro San Giovanni di Dio Fatebenefratelli, Brescia, Italy; [17]Department of Neuroscience, Psychology, Drug Research and Child Health, University of Florence, Florence, Italy; [18]MAC-Memory Clinic and Molecular Markers Laboratory, IRCCS Istituto Centro San Giovanni di Dio Fatebenefratelli, Brescia, Italy; [19]University of Sheffield, Sheffield, UK; [20]Research Center and Memory Clinic. Ace Alzheimer Center Barcelona – Universitat Internacional de Catalunya, Barcelona, Spain; [21]CIBERNED, Network Center for Biomedical Research in Neurodegenerative Diseases, National Institute of Health Carlos III, Madrid, Spain; [22]Department of Neurology, Mayo Clinic Rochester, Rochester, MN, USA; [23]Alzheimer's Disease and Other Cognitive Disorders Unit, Service of Neurology. Hospital Clínic de Barcelona, Fundació Clínic Barcelona-IDIBAPS, Barcelona, Spain; [24]Department of Clinical and Experimental Sciences, University of Brescia, Brescia, Italy; [25]Department of Neurology and Clinical Neurophysiology, University Hospital of Trondheim, Trondheim, Norway; [26]Department of Neuromedicine and Movement Science, Faculty of Medicine and Health Sciences, Norwegian University of Science and Technology, Trondheim, Norway; [27]Neuroscience Research Australia, and Randwick Clinical Campus, UNSW Medicine and Health, University of New South Wales, Sydney, Australia; [28]Regional Neurogenetic Centre, ASPCZ, Lamezia Terme, Italy; [29]Unit of Neurology (V) and Neuropathology, Fondazione IRCCS Istituto Neurologico Carlo Besta, Milano, Italy; [30]Center for Alzheimer's and Related Dementias, National Institute on Aging and National Institute of Neurological Disorders and Stroke, National Institutes of Health, Bethesda, MD, USA; [31]Memory Unit, Neurology

(Affiliations continued on next page)

© 2024 The Author(s). This is an open access article under the CC BY license (http://creativecommons.org/licenses/by/4.0/).

Alfonso di Costanzo,[13] Dennis W. Dickson,[15] Janine Diehl-Schmid,[38,39] Carol Dobson-Stone,[37,40] Oriol Dols-Icardo,[31,21] Aldo Donizetti,[41] Elise Dopper,[42] Elisabetta Durante,[43] Camilla Ferrari,[17] Gianluigi Forloni,[6] Francesca Frangipane,[28] Laura Fratiglioni,[44,45] Milica G. Kramberger,[46,47] Daniela Galimberti,[48,14] Maurizio Gallucci,[49] Pablo García-González,[20,21] Roberta Ghidoni,[16] Giorgio Giaccone,[29] Caroline Graff,[44,50] Neill R. Graff-Radford,[51] Jordan Grafman,[52] Glenda M. Halliday,[37,40] Dena G. Hernandez,[53] Lena E. Hjermind,[54] John R. Hodges,[37] Guy Holloway,[55] Edward D. Huey,[56] Ignacio Illán-Gala,[31,21] Keith A. Josephs,[22] David S. Knopman,[22] Mark Kristiansen,[57,58,59] John B. Kwok,[37,40] Isabelle Leber,[60,61] Hampton L. Leonard,[30,62,63] Ilenia Libri,[24] Alberto Lleo,[31,21] Ian R. Mackenzie,[64,65] Gaganjit K. Madhan,[57,58,59] Raffaele Maletta,[28] Marta Marquié,[20,21] Ales Maver,[66] Manuel Menendez-Gonzalez,[7,8,67] Graziella Milan,[68,122] Bruce L. Miller,[69,70,71] Christopher M. Morris,[72] Huw R. Morris,[2] Benedetta Nacmias,[17,73] Judith Newton,[55] Jørgen E. Nielsen,[54] Christer Nilsson,[74] Valeria Novelli,[75] Alessandro Padovani,[24] Suvankar Pal,[55] Florence Pasquier,[76,77,78] Pau Pastor,[79,80] Robert Perneczky,[81,82,83,84,85] Borut Peterlin,[66] Ronald C. Petersen,[22] Olivier Piguet,[37,86] Yolande AL. Pijnenburg,[35,36] Annibale A. Puca,[87,88] Rosa Rademakers,[15,89,90] Innocenzo Rainero,[91,92] Lianne M. Reus,[93,35,36] Anna MT. Richardson,[94] Matthias Riemenschneider,[95] Ekaterina Rogaeva,[96] Boris Rogelj,[97,98] Sara Rollinson,[99] Howard Rosen,[100] Giacomina Rossi,[29] James B. Rowe,[101] Elisa Rubino,[91,92] Agustin Ruiz,[20,21] Erika Salvi,[102,103] Raquel Sanchez-Valle,[23] Sigrid Botne Sando,[25,26] Alexander F. Santillo,[104] Jennifer A. Saxon,[94] Johannes CM. Schlachetzki,[105] Sonja W. Scholz,[106,107] Harro Seelaar,[42] William W. Seeley,[100] Maria Serpente,[14] Sandro Sorbi,[17,73] Sabrina Sordon,[95] Peter St George-Hyslop,[96,108]

(Author list continued on next page)

Department and Sant Pau Biomedical Research Institute, Hospital de la Santa Creu i Sant Pau, Universitat Autònoma de Barcelona, Barcelona, Spain; [32]Department of Psychiatry, Washington University School of Medicine, St. Louis, MO, USA; [33]NeuroGenomics and Informatics Center, Washington University School of Medicine, St. Louis, MO, USA; [34]Neurologische Klinik, LMU Klinikum, Munich, Germany; [35]Alzheimer Center Amsterdam, Neurology, Vrije Universiteit Amsterdam, Amsterdam UMC location VUmc, Amsterdam, the Netherlands; [36]Amsterdam Neuroscience, Neurodegeneration, Vrije Universiteit Amsterdam, Amsterdam UMC location VUmc, Amsterdam, the Netherlands; [37]Brain and Mind Centre, University of Sydney, Sydney, NSW, Australia; [38]Department of Psychiatry and Psychotherapy, Klinikum rechts der Isar, Technical University of Munich, School of Medicine, Munich, Germany; [39]kbo-Inn-Salzach-Klinikum, Wasserburg, Germany; [40]School of Medical Sciences, University of Sydney, Sydney, NSW, Australia; [41]Department of Biology, University of Naples Federico II, Naples, Italy; [42]Department of Neurology & Alzheimer Center, Erasmus University Medical Center, Rotterdam, the Netherlands; [43]Immunohematology and Transfusional Medicine Service, Local Health Authority n.2 Marca Trevigiana, Treviso, Italy; [44]Karolinska Institutet, Department NVS, KI-Alzheimer Disease Research Center, Stockholm, Sweden; [45]Theme Inflammation and Aging, Karolinska Universtiy Hospital, Stockholm, Sweden; [46]Department of Neurology, University Medical Center, Medical faculty, Ljubljana University of Ljubljana, Ljubljana, Slovenia; [47]Karolinska Institutet, Department of Neurobiology, Care Sciences and Society (NVS), Division of Clinical Geriatrics, Huddinge, Sweden; [48]Department of Biomedical, Surgical and Dental Sciences, University of Milan, Milan, Italy; [49]Cognitive Impairment Center, Local Health Authority n.2 Marca Trevigiana, Treviso, Italy; [50]Unit for hereditary dementia, Karolinska Universtiy Hospital-Solna, Stockholm, Sweden; [51]Department of Neurology, Mayo Clinic Jacksonville, Jacksonville, FL, USA; [52]Shirley Ryan AbilityLab, Chicago, IL, USA; [53]Laboratory of Neurogenetics, National Institute on Aging, National Institutes of Health, Bethesda, MD, USA; [54]Neurogenetics Clinic & Research Lab, Danish Dementia Research Centre, Copenhagen University Hospital, Copenhagen, Denmark; [55]Anne Rowling Regenerative Neurology Clinic, University of Edinburgh, Edinburgh, UK; [56]Bio Med Psychiatry & Human Behavior, Brown University, Providence, RI, USA; [57]UCL Genomics, London, UK; [58]UCL Great Ormond Street Institute of Child Health, London, UK; [59]Zayed Centre for Research into Rare Disease in Children, London, UK; [60]Sorbonne Université, INSERM U1127, CNRS 7225, Institut du Cerveau - ICM, Paris, France; [61]AP-HP Sorbonne Université, Pitié-Salpêtrière Hospital, Department of Neurology, Institute of Memory and Alzheimer's Disease, Paris, France; [62]Data Tecnica International LLC, Washington, DC, USA; [63]DZNE Tübingen, Tübingen, Germany; [64]Department of Pathology, University of British Columbia, Vancouver, Canada; [65]Department of Pathology, Vancouver Coastal Health, Vancouver, Canada; [66]Clinical institute of Genomic Medicine, University Medical Center Ljubljana, Ljubljana, Slovenija; [67]Universidad de Oviedo, Medicine Department, Oviedo, Spain; [68]Geriatric Center "Frullone" ASL NA1, Naples, Italy; [69]Memory and Aging Center, Department of Neurology, Weill Institute for Neurosciences, University of California, San Francisco, San Francisco, CA, USA; [70]Global Brain Health Institute, University of California, San Francisco, San Francisco, CA, USA; [71]Trinity College Dublin, Dublin, Ireland; [72]Newcastle Brain Tissue Resource, Newcastle University, Edwardson Building, Nuns Moor Road, Newcastle upon Tyne, UK; [73]IRCCS Fondazione Don Carlo Gnocchi, Florence, Italy; [74]Department of Clinical Sciences, Neurology, Lund University, Lund/Malmö, Sweden; [75]Centro Cardiologico Monzino IRCCS, Milan, Italy; [76]University of Lille, Lille, France; [77]CHU Lille, Lille, France; [78]Inserm, Labex DISTALZ, LiCEND, Lille, France; [79]Unit of Neurodegenerative Diseases, Department of Neurology, University Hospital Germans Trias i Pujol, Badalona, Barcelona, Spain; [80]The Germans Trias i Pujol Research Institute (IGTP) Badalona, Barcelona, Spain; [81]Department of Psychiatry and Psychotherapy, LMU Hospital, Ludwig-Maximilians-Universität Munich, Munich, Germany; [82]German Center for Neurodegenerative Diseases (DZNE) Munich, Munich, Germany; [83]Munich Cluster for Systems Neurology (SyNergy), Munich, Germany; [84]Ageing Epidemiology (AGE) Research Unit, School of Public Health, Imperial College London, London, UK; [85]Sheffield Institute for Translational Neuroscience (SITraN), University of Sheffield, Sheffield, UK; [86]School of Psychology, University of Sydney, Sydney, NSW, Australia; [87]Department of Medicine, Surgery and Dentistry "Scuola Medica Salernitana," University of Salerno, Fisciano, Italy; [88]Cardiovascular Research Unit, IRCCS MultiMedica, Milan, Italy; [89]VIB Center for Molecular Neurology, VIB, Antwerp, Belgium; [90]Department of Biomedical Sciences, University of Antwerp, Antwerp, Belgium; [91]Department of Neuroscience, "Rita Levi Montalcini," University of Torino, Torino, Italy; [92]Center for Alzheimer's Disease and Related Dementias, Department of Neuroscience and Mental Health, A.O.UCittà della Salute e della Scienza di Torino, Torino, Italy; [93]Center for Neurobehavioral Genetics, Semel Institute for Neuroscience and Human Behavior, University of California, Los Angeles, Los Angeles, CA, USA; [94]Manchester Centre for Clinical Neurosciences, Northern Care Alliance NHS Trust, Manchester Academic Health Sciences Unit, University of Manchester, Manchester, UK; [95]Department of Psychiatry, Saarland University, Homburg, Germany; [96]Tanz Centre for Research in Neurodegenerative Diseases and Department of Medicine, University of Toronto, Toronto, ON, Canada; [97]Department of Biotechnology, Jožef Stefan Institute, Ljubljana, Slovenia; [98]Faculty of Chemistry and Chemical Technology, University of Ljubljana, Ljubljana, Slovenia; [99]Division of Neuroscience and Experimental Psychology, School of Biological Sciences, University of Manchester, Manchester, UK; [100]Department of Neurology, University of California, San Francisco, San Francisco, CA, USA; [101]University of Cambridge Department of Clinical Neurosciences and Cambridge University Hospitals NHS Trust, Cambridge, UK; [102]Unit of Neuroalgologia (III), Fondazione IRCCS Istituto Neurologico Carlo Besta, Milano, Italy; [103]Data science center, Fondazione IRCCS Istituto Neurologico Carlo Besta, Milan, Italy;

(Affiliations continued on next page)

Jennifer C. Thompson,[94,99] Christine Van Broeckhoven,[109,90] Vivianna M. Van Deerlin,[110] Sven J. Van der Lee,[35,36,111] John Van Swieten,[42] Fabrizio Tagliavini,[29] Julie van der Zee,[109,90] Arianna Veronesi,[43] Emilia Vitale,[112,113] Maria Landqvist Waldo,[114] Jennifer S. Yokoyama,[69,115,70,71] Mike A. Nalls,[30,62] Parastoo Momeni,[116] Andrew B. Singleton,[30,53] John Hardy,[117,118,119,120] and Valentina Escott-Price[3,*]

semantic dementia (SD or semantic variant PPA [svPPA]) and progressive non-fluent aphasia (PNFA or nonfluent/agrammatic variant PPA [nfvPPA]).[3,5] FTD can also overlap with motor-neuron disease (FTD-MND)[6] and share clinical features with progressive supranuclear palsy (PSP; MIM: 601104) and the corticobasal syndrome (CBS; no MIM).[7] Pathologically, Tau and TDP-43 are the most frequent protein aggregates that define the pathological subtypes of FTLD-tau and FTLD-TDP (≤45% and ≤50% of all affected individuals, respectively).[8] Genetically, familial FTD (fFTD; ~30% of all FTD-affected individuals) is predominantly linked to mutations in *MAPT* (MIM: 157140), *GRN* (MIM: 138945), and *C9orf72* (MIM: 614260)[9,10]; of note, *GFRA2* (MIM: 601956) and *TMEM106B* (MIM: 613413) (previously reported in a cohort with TDP-43 pathology[11]) were found to be associated with increased risk in a *GRN* mutation FTD cohort.[12] Sporadic FTD (sFTD; ~70% of all affected individuals) has been associated with genetic risk markers at the *TMEM106B*, *DPP6* (MIM: 126141), *UNC13A* (MIM: 609894) and *HLA-DQA2* (MIM: 613503) loci in cohorts with TDP-43 pathology[11,13] and *HLA-DRs* (MIM: 142860) reported in an sFTD cohort encompassing all clinical subtypes.[14]

Efforts in the field have mainly focused on fFTDs, while fewer studies sought to determine the genetic etiology of apparently sFTDs (discussed in Eichler et al., 2010 and Ferrari et al., 2019[15,16]). In the current work, we analyzed 4,685 sFTD cases and 15,308 controls to identify common genetic determinants contributing to increased risk of sFTD and assess their potential biological impact.

Subjects and methods

Study population

Individuals included in the study were clinically diagnosed with a variant of FTD, including the subtypes bvFTD, SD/svPPA, PNFA/nfvPPA, FTD-MND, and FTD-unspecified (i.e., if individuals were diagnosed with FTD but could not be assigned to a specific subtype; see introduction section for acronym definitions). Diagnoses were made according to international consensus criteria: Neary et al. (for FTD, until 2011), Rascovsky et al. (for bvFTD), Gorno-Tempini et al. (for PPA, svPPA and nfvPPA), and Strong et al. (for FTD-MND).[3–5,17] Individuals with logopenic variant PPA were excluded because of its major association with AD.

A previous FTD cohort, divided into discovery (cohort I) and replication (cohort II) sets,[14] was further elaborated by (1) accruing updated metadata leading to the exclusion of individuals that did not meet the diagnostic criteria detailed above, or for which updates were not provided, and (2) inclusion of additional sets of controls. A new cohort of samples (cohort III) was progressively collected between 2016 and 2019 by clinicians and research groups based in Europe (Belgium, France, Germany, Italy, the Netherlands, Norway, Slovenia, Spain, Sweden, and UK) and North America (USA and Canada). Each contributing site obtained written informed consent for the samples to be part of this genetic study (IRB approval #9811/001). All samples (cohorts I, II, and III) were progressively sent as extracted DNA from tissues (blood and/or brain) and stored at −80°C upon receipt (see also Ferrari et al., 2014[14]). The bulk of the control samples used in this study (from France, Germany, Italy, the Netherlands, Spain, Sweden, UK, and USA) was available through a previous study,[14] and additional control data were obtained from collaborators (at National Institutes of Health, USA and University College London, UK) and genotyped during the cohort III genotyping iterations (also including additional controls from Italy, Norway, and Slovenia). Overall, the control population at hand consisted of 16,821 samples (from France, Germany, Italy, the Netherlands, Spain, Sweden, UK, USA, Norway, and Slovenia), free of neurological illness at the time of sampling, and matched to cases based on population ancestry (see also QC—Samples below).

Cohorts

Before quality control checks (QCs), cohort I included 2,026 cases and 8,387 controls, cohort II 1,121 cases and 5,091 controls, and cohort III 2,504 cases and 3,343 controls. The three

[104]Department of Clinical Sciences, Clinical Memory Research Unit, Faculty of Medicine, Lund University, Lund/Malmö, Sweden; [105]Department of Cellular and Molecular Medicine, University of California, San Diego, La Jolla, CA, USA; [106]Neurodegenerative Diseases Research Unit, National Institute of Neurological Disorders and Stroke, Bethesda, MD, USA; [107]Department of Neurology, Johns Hopkins University Medical Center, Baltimore, MD, USA; [108]Department of Neurology, Columbia University, New York, NY, USA; [109]Neurodegenerative Brain Diseases, VIB Center for Molecular Neurology, VIB, Antwerp, Belgium; [110]Perelman School of Medicine at the University of Pennsylvania, Department of Pathology and Laboratory Medicine, Center for Neurodegenerative Disease Research, Philadelphia, PA, USA; [111]Section Genomics of Neurodegenerative Diseases and Aging, Department of Clinical Genetics, Vrije Universiteit Amsterdam, Amsterdam UMC, Amsterdam, the Netherlands; [112]Institute of Biochemistry and Cell Biology, National Research Council (CNR), Naples, Italy; [113]School of Integrative Science and Technology Department of Biology Kean University, Union, NJ, USA; [114]Clinical Sciences Helsingborg, Department of Clinical Sciences, Lund University, Lund, Sweden; [115]Department of Radiology and Biomedical Imaging, University of California, San Francisco, San Francisco, CA, USA; [116]Rona Holdings, Cupertino, CA, USA; [117]UK Dementia Research Institute at UCL and Department of Neurodegenerative Disease, UCL Queen Square Institute of Neurology, London, UK; [118]Reta Lila Weston Institute, UCL Queen Square Institute of Neurology, London, UK; [119]NIHR University College London Hospitals Biomedical Research Centre, London, UK; [120]Institute for Advanced Study, The Hong Kong University of Science and Technology, Hong Kong SAR, China
[121]These authors contributed equally
[122]Deceased
*Correspondence: c.manzoni@ucl.ac.uk (C.M.), escottpricev@cardiff.ac.uk (V.E.-P.)
https://doi.org/10.1016/j.ajhg.2024.05.017.

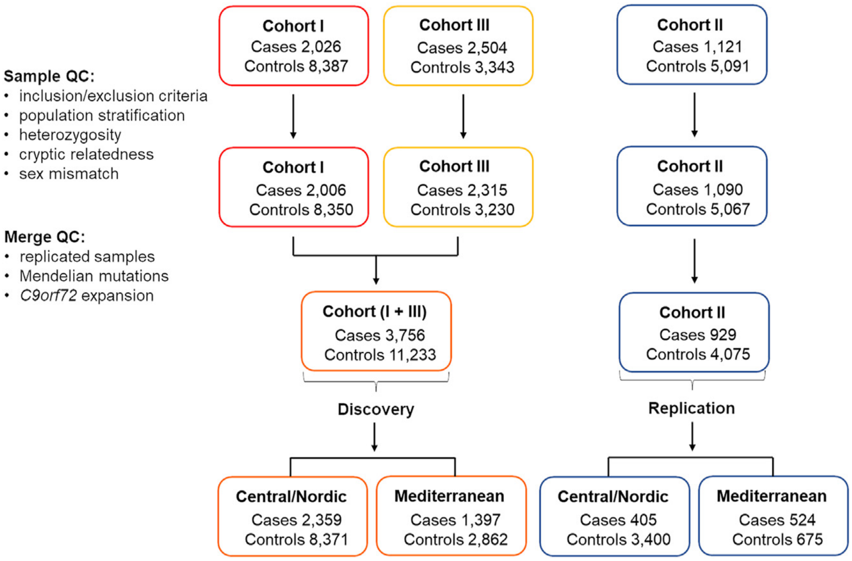

Figure 1. QC pipeline for the three cohorts contributing to discovery and replication
The discovery and replication sets were further subdivided into Central/Nordic and Mediterranean Europeans (PCA-based genetically estimated ancestry) to assess potential population-specific disease risk loci.

cohorts were independently QCed (see below), leaving 2,006 cases and 8,350 controls for cohort I, 1,090 cases and 5,067 controls for cohort II, and 2,315 cases and 3,230 controls for cohort III.

Cohorts I and III (run on genotyping chips) were combined into a new discovery cohort. Cohort II (run on NeuroX array[14,18]) was used as replication set. Duplicated samples and samples known to carry Mendelian mutations in neurodegenerative genes (*APP* [MIM: 104760], *CHCHD10* [MIM: 615903], *CHMP2B* [MIM: 609512], *FUS* [MIM: 137070], *GRN*, *HNRNPA1* [MIM: 164017], *LRRK2* [MIM: 609007], *MAPT*, *NOTCH3* [MIM: 600276], *PSEN1* [MIM: 104311], *PSEN2* [MIM: 600759], *SERPINI1* [MIM: 602445], *SORL1* [MIM: 602005], *SQSTM1* [MIM: 601530], *TMEM106B*, *TBK1* [MIM: 604834], *VCP* [MIM: 601023]), or a *C9orf72* pathogenic expansion, were removed from the analyses, leading to the following final study cohorts (before sample QC): 3,756 cases and 11,233 controls for the discovery phase (cohorts I + III) and 929 cases and 4,075 controls for the replication phase (cohort II). A breakdown of samples (and FTD subtypes) is shown in Table S1. The three cohorts, the QC procedures, and the discovery and replication sets are shown in Figure 1. Of note, given the small number of samples for some of the FTD subtypes (SD, PNFA, and FTD-MND), subtype analysis was not informative, and therefore it is not reported.

Sample genotyping

Cohort I cases had been genotyped on either 660K or OmniExpress Illumina array chips.[14] Cohort II cases had been genotyped on the NeuroX array.[14,18] Cohort III cases were genotyped on NeuroChip.[19] Samples were genotyped at the Laboratory of Neurogenetics of the National Institute on Aging, NIH or at the core facility at the Institute of Child Health, UCL (UCL Genomics). All arrays were run on the Illumina Infinium platform as per the manufacturer's instructions. Control samples had been genotyped using a variety of array chips, including 330K, 550K, 660K, OmniExpress, NeuroX, and NeuroChip, and cohorts I and II were previously genotyped as per Ferrari et al., 2014;[14] cohort III samples were genotyped within the current study (IRB approval #9811/001). Data were QCed following standard procedure using samples with genotyping call rate ≥95% and markers with GenTrain score ≥0.7.

QC—Samples

Sample-level QC was performed before carrying out separate imputation for each of the three cohorts. Genotypes were used to inform on population substructure via principal-component analysis (PCA). Linkage disequilibrium (LD)-pruned markers with a 95% genotyping rate (less than 5% missing), Hardy-Weinberg equilibrium exact test (HWE) p value $\geq 1 \times 10^{-10}$ midp,[20] and minor allele frequency (MAF) ≥0.01 were used to assess ancestry via PCA against HapMap Phase3 (hapmap3_r3_b36_fwd.consensus.qc.poly). This analysis allowed us to address population substructure and led to the exclusion of population outliers; briefly, PCA on the three different cohorts was performed, and overlap with European samples from HapMap was used to identify outliers (see details in Figure S1).

We also assessed samples' potential contamination by evaluating individuals' heterozygosity (removing samples with inbreeding coefficient estimates outside 4 standard deviations from the mean distribution) and cryptic relatedness (removing sample pairs with estimated identity by descent = PI_HAT greater than 0.125) using a set of LD-pruned high-quality SNPs (markers with a 95% genotyping rate, HWE $\geq 1 \times 10^{-10}$ midp, and MAF ≥0.01). Finally, we excluded samples with a possible mismatch for sex by assessing the X chromosome heterozygosity (for cohorts II and III only, as for cohort I genotyping for the X chromosome was not available).

QC—Markers

Variant-level QC cleaning was performed before imputation for each of the three cohorts. Palindromic markers and markers with missing call rates exceeding 5% were removed. Finally, we assessed differences between case and control genotype data via non-random missingness, excluding markers with Bonferroni's corrected p values <0.1 (indicating significant differential missingness between cases and controls).

Imputation

The three cohorts were imputed separately. Cohort I was converted from GRCh36/hg18 to GRCh37/hg19 using the LIFTOVER tool prior to imputation. We imputed markers through the Michigan server (https://imputationserver.sph.umich.edu/) using the following specifications and thresholds: Minimac4; HRC reference panel (GRCh37/hg19); imputation filter rsq 0.3; Eagle v.2.4 phasing; European (EUR) population. Multiallelic and palindromic variants were removed following imputation. Post-imputation dataset sizes were as follows: cohort I, ~22 M markers; cohort II, ~5 M markers; cohort III, ~21 M markers. Imputation for cohort II resulted in a smaller number of imputed markers due to this cohort being genotyped on the NeuroX array, an exome chip not originally designed for imputation.[14,18]

Discovery cohort merge

Relatedness across the three cohorts was evaluated using genotyped-only communal markers (cohorts I ∩ II = 7,480 genotyped communal markers, II ∩ III = 10,328, I ∩ III = 94,773). A set of LD-pruned high-quality SNPs (markers with a 95% genotyping rate, HWE $\geq 1 \times 10^{-10}$ midp, and MAF ≥ 0.01) was used to evaluate cryptic relatedness (removing sample pairs with estimated identity by descent = PI_HAT greater than 0.125). Cohort I and III post-imputation were then combined using the overlapping markers as discovery cohort (3,756 cases and 11,233 controls; ~18 M markers genotyped and imputed), while cohort II was used as replication cohort (929 cases and 4,075 controls; ~5 M markers genotyped and imputed) (Figure S2).

Association analyses

We performed association analysis using markers with MAF >1% and HWE $>10^{-4}$ midp through the PLINK case-control logistic regression association analysis with 20 principal components (PCs), sex, and study cohort (I or III) as covariates. The top markers were confirmed by running a similar association analysis in MAOS (https://dlin.web.unc.edu/software/maos/) using 20 PCs, sex, study cohort (I or III), and FTD subtypes as covariates.[21] The same analytical pipeline was applied to the replication cohort. Markers of interest were meta-analyzed (discovery + replication) using METAL (run with STDERR scheme).[22]

Genome-wide significant markers were annotated using the Ensembl Variant Effect Predictor (VEP). Frequencies for 1000 Genomes (European samples) were used to further control for marker frequencies in the general population: a variation of >15% between our controls and the European general population as per 1000 Genomes was used as threshold to exclude variants from the current study. When single populations from 1000 Genomes were used, they were selected as follows: GBR = British in England and Scotland; CEU = Northern Europeans from Utah; IBR = Iberian populations in Spain; TSI = Tuscans from Italy.

The Bonferroni threshold for genome-wide significance was $p \leq 5 \times 10^{-8}$.[23] Variants with p values between 1×10^{-5} and 5×10^{-8} were reported as suggestive (only loci containing ≥20 markers in those p value ranges).

Heritability and genetic correlation analyses

We used LD score regression (LDSC)[24] to derive an SNP-based heritability estimate (h^2) for the discovery FTD summary statistics (3,756 cases and 11,233 controls). The analysis was performed using the pre-computed European SNP LD scores (https://data.broadinstitute.org/alkesgroup/LDSCORE/eur_w_ld_chr.tar.bz2).

Then, LDSC was employed for estimation of genetic correlation (rg) between FTD and publicly available genome-wide association study (GWAS) summary statistics of five neurodegenerative disorders: (1) clinical AD GWAS of 63,926 samples (AD)[25]; (2) AD clinical/proxy GWAS and related dementias (ADRD) of 487,511 samples[26]; (3) Parkinson Disease GWAS (PD; MIM: 168600) of 1,474,097 samples[27]; (4) amyotrophic lateral sclerosis GWAS (ALS; MIM: 612069) of 138,086 samples[28]; and (5) Lewy body dementia GWAS (LBD; MIM: 127750) of 6,618 samples.[29]

BUHMBOX (https://software.broadinstitute.org/mpg/buhmbox/) was run according to Han et al., 2016.[30] SNPs associated with AD[25] were extracted in the *APOE* (MIM: 107741) region and filtered to remove FTD SNPs with $p < 0.05$. The remaining SNPs were clumped (with $r^2 = 0.1$ in 10,000-kb window), resulting in 64 SNPs for the analysis.

Locus analysis

Risk loci at chromosome 17 (*MAPT* region) and chromosome 19 (*APOE* region) were further characterized by extracting the genotypes of the following markers: rs17650901 (A:G) and rs242557 (G:A) on chromosome 17, where the A alleles tag the H1 and H1c clade, respectively,[31,32] and rs429358 (C:T) and rs7412 (T:C) on chromosome 19 to assess the *APOE* alleles (C/C, C/C = ε4/ε4; T/T, T/T = ε2/ε2; and T/T, C/C = ε3/ε3). Additional controls for the *APOE* markers were downloaded from the 1000 Genomes Project using the Ensembl Genome Browser and obtained from an independent cohort of controls (731 Italian controls and 347 Central/Nordic European controls). Differences in ε allele counts were assessed using Pearson's χ^2 test with Yates' continuity correction.

Functional analysis

We used the full distribution of SNP p values and the top markers (post joint analysis) to further characterize biological and functional effects, including potential effects on expression and splicing, using the GTEx (https://gtexportal.org/) and Functional Mapping and Annotation (FUMA) (https://fuma.ctglab.nl/)[33] platforms.

Software

All analyses were performed using R (R v.3.5.2), R studio (R v.3.6.2, studio v.1.2.1335), PLINK v.1.9, MAOS (http://dlin.web.unc.edu/files/2011/08/maos-1.2-linux.tar_.gz), and METAL (version for Windows, released March 25, 2011). LD score regression was run using LDSC v.1.0.1 (https://github.com/bulik/ldsc).

Results

Discovery association analyses

We performed GWAS analysis for the discovery cohort (3,756 cases and 11,233 controls). The genomic inflation factor (λ) was 1.035 ($\lambda_{1000} = 1.006$) (Figure S3). 1,886 markers, mapping to the *MAPT* (1,880 markers; chr17:43,572,419–44,862,347 [GRCh37/hg19]) and *APOE* (6 markers; chr19:45,387,596–45,396,144 [GRCh37/hg19]) loci, were genome-wide significant.

The SNPs with the lowest p values were rs199443 (*MAPT* locus; $p = 1.03 \times 10^{-9}$; $\beta = 0.229$ [C = risk allele, major allele]; SE = 0.037) and rs6857 (*APOE* locus; $p = 6.2 \times 10^{-10}$; $\beta = 0.239$ [T = risk allele, minor allele]; SE = 0.039) (Figure 2; Table 1; Table S2). After conditioning the regional analyses on the index SNPs, each association disappeared, suggesting there is a unique signal per locus (Figures S4A and S4B). To further support our discovery findings, we used another statistical method (MAOS; see subjects and methods), which allowed us to run five different association analyses, assessing each subtype, accounting for the same control sets, and meta-analyzing the outcomes. The genome-wide-significant hits shown above were confirmed and displayed improved statistics (Table S3).

We identified six suggestive signals comprising at least four markers with $p < 1 \times 10^{-5}$ and sought to take two of them (comprising at least 20 markers with $p < 1 \times 10^{-5}$)

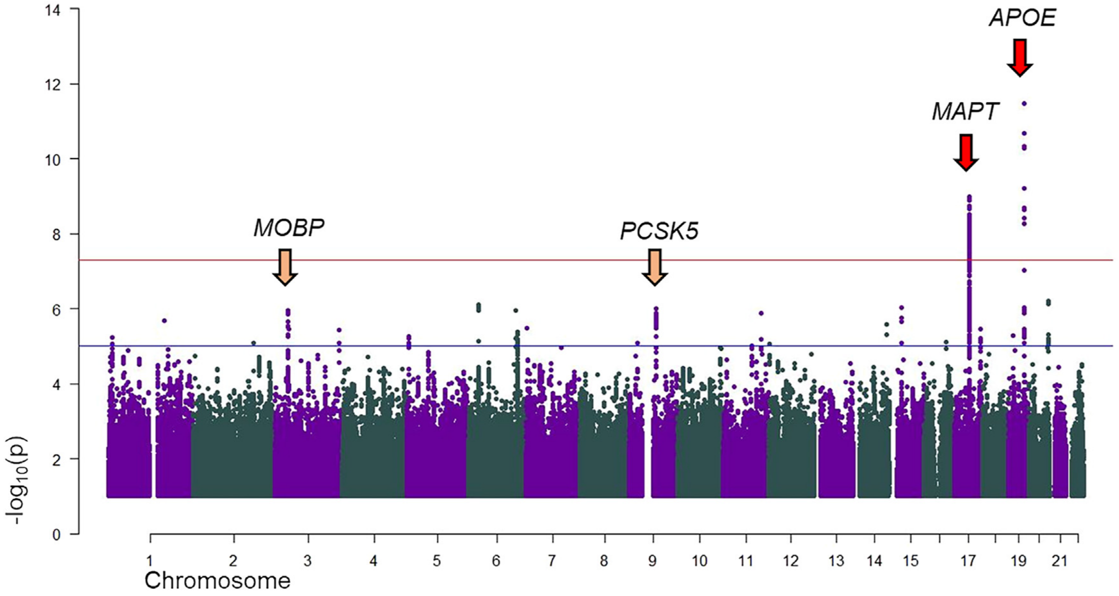

Figure 2. Discovery phase: Manhattan plot
Red arrows identify genome-wide-significant signals (chromosomes 17 and 19); yellow arrows identify suggestive towers ($10^{-8} < p > 10^{-5}$) including at least 20 markers (chromosomes 3 and 9). The plot is cut at $-\log_{10}(p) = 1$. The gene symbols represent the locus and do not necessarily imply functional/biological relevance.

forward for replication and joint analysis to screen for their potential relevance (Table S4): chromosome 3 (top SNP rs13081054; myelin-associated oligodendrocyte basic protein [*MOBP*; MIM: 600948] locus) and chromosome 9 (top SNP rs76573513; *PCSK5* [MIM: 600488] locus).

Replication and joint analyses

The following top markers in discovery analysis ($n = 6$ for the *APOE* locus; $n = 1{,}880$ for the *MAPT* locus; $n = 29$ for the *MOBP* locus) were present in the replication set (see also Tables S2 and S4).

In the replication set, the chromosome 17 and chromosome 19 top SNPs (rs199443 and rs6857, respectively) reached p value $= 5.3 \times 10^{-4}$ ($\beta = 0.267$; SE $= 0.077$) and p value $= 5 \times 10^{-4}$ ($\beta = 0.249$; SE $= 0.072$), respectively. Following joint analysis, each marker was genome-wide significant ($p = 2.5 \times 10^{-12}$; $\beta = 0.236$; SE $= 0.034$ for rs199443 [*MAPT* locus] and $p = 1.31 \times 10^{-12}$; $\beta = 0.241$; SE $= 0.034$ for rs6857 [*APOE* locus]), with the effect being in the same direction (Table 1; Table S2).

For the suggestive signals, only markers on chromosome 3 were available in the replication set (due to lower imputation coverage for the NeuroX exome-chip; see subjects and methods): rs1009966 was replicated and reached lowest p value (genome-wide significant) after joint analysis (p value $= 2.36 \times 10^{-8}$; $\beta = 0.147$; SE $= 0.026$), with the effect being in the same direction (Table S4).

MAPT and *APOE* loci

We further characterized the *MAPT* locus in the discovery cohort using rs17650901 (A:G) and rs242557 (G:A), where the A alleles tag the H1 and H1c clade, respectively. The frequencies of the A allele and the homozygous A/A genotype were significantly increased in cases compared to controls for rs17650901 ($p = 1.9 \times 10^{-9}$ and $p = 7.7 \times 10^{-9}$, respectively) and rs242557 ($p = 5 \times 10^{-3}$ and $p = 8.8 \times 10^{-3}$, respectively), suggesting association of the H1/H1 haplotype and H1c clade with sFTD (Table 2).

The significant markers at chromosome 19 revealed a (genetically estimated) ancestry-related difference in allele frequencies (Table 3): whereas individuals with FTD showed similar frequencies for the chromosome 19 markers regardless of ancestry, Mediterranean European controls showed remarkably decreased frequencies compared to Central/Nordic European controls (Table 3). This trend was further confirmed in an independent cohort of 731 Italian and 347 Continental European controls (Table 3; additional characterization of the *APOE* locus [*APOE* $\mathcal{E}4$ alleles] is included in the supplemental information [Note S1]).

Central/Nordic and Mediterranean European independent analyses

To better understand the population-specific contribution to the discovery analysis results, we split the discovery cohort into Central/Nordic (2,359 cases and 8,371 controls) and Mediterranean Europeans (1,397 cases and 2,862 controls), based on genetically estimated ancestry (PCA based), and performed association analysis for these two sub-cohorts separately (Figures S5 and S6). The genomic inflation factors were $\lambda = 1.0135$ and $\lambda = 1.0356$, respectively.

Association analysis for the Central/Nordic European cohort revealed one genome-wide-significant signal on chromosome 17 at the *MAPT* locus (rs199443; $p = 1.08 \times 10^{-8}$; $\beta = 0.256$ [C = risk allele, major allele]; SE $= 0.045$). In the replication set (also subdivided in

Table 1. Top marker summary statistics at the significant loci

					Discovery								Replication							Joint Analysis					
						Minor allele frequency			PLINK					Minor allele frequency		PLINK				METAL					
rs number	Chr	Base pair	Locus	A1/A2	ImpScore (rsq)[1,2]	Controls	Cases	1000G EUR-	Beta	OR	SE	p value	ImpScore (rsq)	Controls	Cases	Beta	OR	SE	p value	Beta	OR	SE	p value	I^2	HetPVal
rs199443	17	44,819,565	*MAPT*	T/C*	0.96, 0.96	0.224	0.187	0.223	0.229	1.257	0.037	1.03E-09	genotyped	0.220	0.210	0.267	1.306	0.077	5.34E-04	0.236	1.266	0.034	2.50E-12	0	6.53E-01
rs6857	19	45,392,254	*APOE*	T*/C	0.96, 0.98	0.151	0.179	0.165	0.239	1.270	0.039	6.22E-10	0.99	0.161	0.181	0.249	1.283	0.072	5.01E-04	0.241	1.273	0.034	1.31E-12	0	9.00E-01
rs1009966	3	39,473,591	*MOBP*	G/A*	0.93, 0.97	0.475	0.433	0.460	0.144	1.155	0.030	1.12E-06	0.77	0.469	0.413	0.157	1.17	0.057	6.18E-03	0.147	1.158	0.026	2.36E-08	0	8.47E-01

Discovery, replication, and joint analysis statistics are shown for the top markers (smallest p value) at the significant loci. A1, minor allele; *, risk allele; ImpScore (rsq),[1,2] imputation scores in phases I and III combined in discovery; 1000G, 1000 Genomes.

Central/Nordic [405 cases and 3,400 controls] and Mediterranean Europeans [524 cases and 675 controls]), the chromosome 17 top SNP reached p value $= 2.19 \times 10^{-2}$ ($\beta = 0.249$; SE $= 0.109$), and joint analysis confirmed genome-wide-significant statistics ($p = 8.02 \times 10^{-10}$; $\beta = 0.256$; SE $= 0.042$), with the effect being in the same direction (Figure 3; Table 4). The *APOE* locus signal on chromosome 19 (rs6857) was not genome-wide significant (p value $= 7.9 \times 10^{-5}$) in the discovery Central/Nordic European cohort; it reached p value $= 2.55 \times 10^{-2}$ in the replication set and remained not significant ($p = 6.8 \times 10^{-6}$; $\beta = 0.183$; SE $= 0.041$) after joint analysis (Table 4).

Association analysis for the Mediterranean cohort did not yield significant results (probably because of power issues due to the small Mediterranean cohort size). However, it is worth noting that, although the signal on chromosome 17 showed $p_{\text{joint}} = 6.80 \times 10^{-3}$, the signal on chromosome 19 was suggestive ($p_{\text{joint}} = 7.67 \times 10^{-7}$; $\beta = 0.338$; SE $= 0.068$) (Figure S7; Table 4).

To further shed light on the *APOE* locus signal on chromosome 19 (rs6857) in the two cohorts, we performed χ^2 test (cases vs. controls) for rs6857 in the Central/Nordic European and Mediterranean European cohorts (Figure 4): the unadjusted p value for the former group was 1×10^{-3}, while for the latter it was 1.2×10^{-14} (and driven by the control frequencies).

In summary, taken together, these data suggest that, in our extended cohort, the signal at the *MAPT* locus appeared to be mainly driven by the Central/Nordic European cohort and that at the *APOE* locus by the Mediterranean European cohort.

Functional analysis

We found no meaningful impacted biological pathways using the full distribution of SNP p values (Table S5). The assessment of potential effects on expression and splicing (e/sQTL) exerted by the risk markers highlighted in the current study revealed the following (Table S6): (1) there was no significant effect on expression or splicing of in-*cis* genes for the *APOE* locus marker (rs6857); (2) the *MAPT* locus marker (rs199443) revealed effects on both expression and splicing, in *cis*, in brain cortex, affecting genes involved in transcription regulation (e.g., KANSL1 antisense RNA 1 [*KANSL1-AS1*; MIM: 612452]), protein trafficking (e.g., ADP ribosylation factor-like GTPase 17A [*ARL17A*]), and signal transduction (e.g., corticotropin-releasing hormone receptor 1 [*CRHR1*; MIM: 122561]), as well as *MAPT* itself; and (3) the top marker on chromosome 3 (rs1009966) revealed effects on expression in the cerebellum and splicing in brain cortex of the ribosomal protein SA gene (*RPSA*; MIM: 150370); the RPSA protein is involved in stabilizing ribosomal subunits and in the signal transduction as a cell surface receptor for laminin.[34]

Heritability and genetic correlation analyses

Heritability estimation with LDSC regression for FTD summary statistics returned $h^2 = 0.118$ (se $= 0.02$) (Table S7A).

Table 2. Haplotype analysis at the *MAPT* locus

Marker	Genotype	Haplotype	Cases Count	Cases Freq	Controls Count	Controls Freq	χ^2 A	χ^2 AA
chr17:44039691A>G (rs17650901)	G/G	H2/H2	163	0.04	644	0.06	1.9×10^{-9}	7.7×10^{-9}
	G/A	H1/H2	1,221	0.33	4,098	0.36		
	A/A	H1/H1	2,372	0.63	6,491	0.58		
chr17:44019712G>A (rs242557)	A/A	H1c/H1c	509	0.14	1,338	0.12	5×10^{-3}	8.8×10^{-3}
	A/G	H1/H1c	1,733	0.46	5,148	0.46		
	G/G	H1/H1	1,514	0.40	4,747	0.42		

The H1 and H2 haplotype distribution is shown for cases and controls. Count, number of subjects; freq, frequency of the haplotype.

Genetic correlation of FTD with five other traits related to neurodegenerative diseases revealed positive significant correlation with all of them ($p < 0.05$), with the exception of Alzheimer disease-related dementia (ADRD) GWAS[26] (see Table S7B). The largest genetic correlations were with LBD (rg = 0.91), ALS (rg = 0.71), and AD (rg = 0.55), and the overall results of this analysis indicated substantial shared genetic liability or potential misdiagnoses of FTD with all of the following conditions: LBD, ALS, AD, and PD. BUHMBOX was run to compare the *APOE* region (chr19:44.4–46.5 Mb) between AD and FTD, resulting in $p = 0.006$ and Mendelian randomization (MR) $p = 5.8 \times 10^{-6}$.

Cross-check of markers across different neurodegenerative diseases

We sought to verify the statistics of key markers previously identified in other neurodegenerative conditions (etiologically close to FTD) in our current dataset (Table S8). The *TMEM106B* and *GFRA2* markers[11,12] showed negligible association in our dataset (likely because of the current study design, i.e., clinical cohort excluding individuals with *GRN* mutations). Some of the significant ALS markers as shown in van Rheenen et al., 2021 and Nicolas et al., 2018[28,35] reached $p = 1.07 \times 10^{-3}$ and $p = 1.6 \times 10^{-4}$ for the *UNC13A* (MIM: 609894) and *MOBP* loci markers (rs12973192 [proxy] and rs631312, respectively) with similar effects sizes (OR = 1.1), while the *C9orf72* marker (rs3849943, highly significant in ALS) showed negligible association in our dataset (probably because of the current study design, i.e., individuals with *C9orf72* expansion were excluded from the study). Most historically established AD markers—including rs6656401 and rs679515 (*CR1*; MIM: 120620), rs6733839 (*BIN1*; MIM: 601248), rs9331896 and rs11787077 (*CLU*; MIM: 185430), and rs6605556 (*HLA*)[26,36,37]—showed negligible association. *PICALM* (rs3851179; MIM: 603025) showed $p = 6.5 \times 10^{-3}$ and, interestingly, *MAPT* (rs199515) $p = 1.1 \times 10^{-9}$.[26,36]

In addition, for the *APOE* locus, we assessed several markers that were extensively reported as being genome-wide significant in AD (rs4420638, rs439401, and rs7412)[37,38]: one of these markers showed significant *p* value levels, though displaying smaller effect size in the current cohort (rs4420638; $p = 9.6 \times 10^{-8}$ with OR = 3.95 in AD and 1.2 in FTD), while the other two did not (rs439401; $p = 8.1 \times 10^{-1}$ and rs7412; $p = 1.9 \times 10^{-4}$). Conversely, we also sought to verify the *MOBP* hit reported as a novel potential FTD locus in the current work (rs1009966) in AD datasets: it showed negligible *p* values in two recent AD GWASs, i.e., $p = 3.4 \times 10^{-1}$ and 7.5×10^{-1}.[25],[26] A previously reported hit at the *HLA* locus[14] reached suggestive significance (rs9268877; $p = 9.57 \times 10^{-7}$). Furthermore, whereas several previously reported PSP and CBD risk variants[39–41] showed negligible association, some PSP risk markers reached *p* values in the range of 10^{-3} (rs1411478 [*STX6*; MIM: 603944] and rs11568563 [*SLCO1A2*; MIM: 602883]) and 10^{-4} (rs1768208 [*MOBP*]) in our dataset. Finally, the *MAPT* locus risk variants previously reported in PD, AD, PSP, and CBD[26,39,40,42] all resulted genome-wide significant in our dataset.

Discussion

In the current work, we analyzed 4,685 sFTD cases and 15,308 controls, looking for common genetic determinants contributing to increased risk of sFTD. Compared to the previous work,[14] we here increased sample size and improved the cohort (updated diagnoses) and provided insights highlighting a cluster of variants at the *MAPT* (rs199443; chromosome 17) and *APOE* (rs6857; chromosome 19) loci and a candidate locus on chromosome 3 (rs1009966 in the intergenic region between *RPSA* and *MOBP*) contributing to increased risk for sFTD by potentially mediating effects on expression and splicing of functionally relevant in-*cis* genes at the *MAPT*[43] and *RPSA-MOBP* loci.

Further analysis of the *MAPT* locus suggested an increase of the H1/H1 haplotype and of the H1c clade in individuals with sFTD. This signature was previously reported in two small FTD cohorts from France and the UK,[44,45] and it also appears to be consistent across different neurodegenerative conditions, as the H1 haplotype was shown to be associated with AD,[46,47] CBD, PSP,[48–50] and PD.[51] This, and the fact that the *MAPT* locus risk variants

Table 3. Ancestry-related difference at the *APOE* locus

						Minor allele frequency								
						Discovery		1000G	Discovery Nordic/Central European		Independent Nordic/ Central European cohort	Discovery Mediterranean		Independent Italian cohort
Chr	rs number	Base pair	*p* value	OR	Allele	Cases	Controls	EUR	Cases	Controls	Controls	Cases	Controls	Controls
19	rs6857	45,392,254	6.22E-10	1.27	T	0.1793	0.1508	0.165	0.1855	0.1651	0.1686	0.1689	0.109	0.1204
19	rs12972970	45,387,596	2.10E-09	1.28	A	0.1531	0.1283	0.1352	0.1598	0.1422	0.147	0.1417	0.0877	0.1026
19	rs34342646	45,388,130	2.35E-09	1.276	A	0.1571	0.1321	0.1392	0.1636	0.1455	0.147	0.146	0.09294	0.1026
19	rs71352238	45,394,336	3.87E-09	1.273	C	0.155	0.1304	0.1332	0.1624	0.1444	0.1527	0.1424	0.08945	0.1033
19	rs2075650	45,395,619	5.29E-09	1.272	G	0.1523	0.128	0.1312	0.1598	0.1427	0.1455	0.1396	0.08491	0.1033
19	rs11556505	45,396,144	5.50E-09	1.272	T	0.1519	0.1278	0.1312	0.1594	0.1426	0.1455	0.1392	0.08456	0.1033

The allele frequencies of the significant markers at the APOE locus are shown in different cohorts (the entire discovery cohort; the 1000 Genomes European cohort, the discovery cohort divided into Central European/Nordic and Mediterranean European populations, and independent samples of Central European/Nordic and Mediterranean European ancestry). 1000G, 1000 Genomes.

previously reported in PD, AD, PSP, and CBD[26,39,40,42] all resulted genome-wide significant in our dataset (and in complete and perfect LD with each other), supports the notion that the *MAPT* locus may be a common denominator at the crossroad of multiple etiologically close conditions such as FTD, AD,[26] PSP, CBD,[50] and PD.[52]

The association with the *RPSA-MOBP* locus appears of particular interest considering its involvement in PSP[39] and ALS.[28] Our reported marker with the lowest p value after joint analysis (rs1009966; $p = 2.36 \times 10^{-8}$, OR $= 1.16$) is in complete ($D' = 1$) although not perfect ($R^2 = 0.3$) LD in the European population (LDlink [https://ldlink.nih.gov/?tab=home] CEU 1000 Genomes, GRCh37) with the PSP (rs1768208) and ALS (rs631312) genome-wide-significant markers, suggesting potential pleiotropy at this locus across a subset of individuals with FTD, PSP, and ALS.

The signal at the *APOE* locus appeared to be population specific and driven by the control frequencies. More specifically, although the frequencies of the markers at the *APOE* locus were relatively similar in the Central/Nordic European (18.5%) and Mediterranean European (16.9%) cases making up the study cohort, they were significantly less frequent in Mediterranean (10.9%) compared to Central/Nordic European (16.5%) controls. The frequency patterns observed in our Central/Nordic vs. Mediterranean European controls were further supported by the 1000 Genomes cohort (IBR + TSI vs. CEU + GBR general populations), an independent cohort of controls (Italian and Central/Nordic Europeans), and a population-specific study analyzing *APOE* frequencies in the Treviso longevity (TRELONG) longitudinal study.[53] All this taken together may suggest there being a genuine variation in the genetic architecture of these two (close yet different) European population groups at this locus.[54,55] The link to the *APOE* locus could lead to different explanations/interpretations: (1) it may reflect a presence of individuals in our cohort with behavioral variant AD that mimics FTD[56,57]; (2) it may underpin comorbidities at play, given that some individuals with FTD coming to autopsy can show concurrent AD or vascular changes in addition to changes attributed directly to FTD[58–60]; (3) it may represent a genuine association pertaining to a population-specific subset of individuals with FTD. In relation to this latter point, it is worth noting that, per study design, we excluded all samples diagnosed with the logopenic variant in the current work (thus reducing potential diagnosis bias), and we verified that many of the historical AD risk loci (e.g., *CR1*, *BIN1*, *CLU*) reached negligible p values in our cohort, while the *MOBP* locus hit reported for FTD in the current work shows negligible p values in two recent AD GWASs. Moreover, a literature survey supports the notion of the involvement of the *APOE* locus in some forms of FTD.[52,61–64]

Previous work[14] reported association with the *HLA* locus; in the current analyses, although genome-wide-significant levels are not reached, we show rs9268863 as the top SNP at the *HLA* locus ($p = 7.86 \times 10^{-7}$, OR $= 1.2$; A $=$ risk allele) in the discovery analysis, with rs9268877 and rs9268863

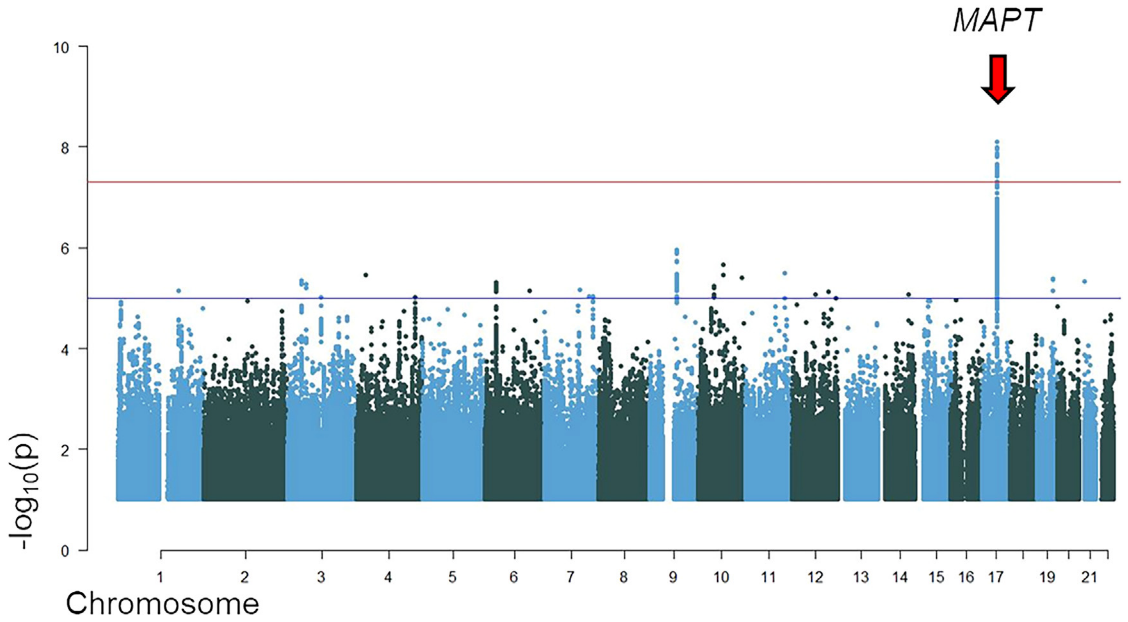

Figure 3. Manhattan plot for discovery-phase Central/Nordic European cohort
Red arrow indicates the genome-wide significant signal (chromosome 17). The plot is cut at $-\log_{10}(p) = 1$. The gene symbols represent the locus and do not necessarily imply functional/biological relevance.

being in complete (D′ = 1) and perfect ($R^2 = 1$) LD in the European population (LDlink [https://ldlink.nih.gov/?tab=home] CEU 1000 Genomes, GRCh37). Clearly, despite other work supporting the notion of an involvement of immune system processes in the pathogenesis of sFTD,[65] additional studies are needed to shed light on the level and type of involvement of the *HLA* locus in sFTD.

In summary, we report that the *MAPT* (pointing to the H1c clade), *APOE*, and *RPSA-MOBP* loci contribute to increased genetic risk of sFTD pathogenesis. Notably, the involvement of the *MAPT* locus in sFTD, and its association also with AD, PSP, PD, and CBD, strongly suggests potential common genetic pleiotropy for these neurological conditions at this locus. Moreover, our results pointing at the *RPSA-MOBP* locus shed light on an additional potential genetic overlap between FTD and PSP and between FTD and ALS. It is worth noting that the diagnosis of neurodegenerative diseases is challenging due to subtle overlap of clinical presentations[62]; therefore, future studies will need to be powered enough to allow for further assessment of pleiotropy vs. potential misdiagnosis (e.g., through structural equation modeling[66]). Moreover, an interesting way forward will be to not only continue addressing the genetic etiology of familial vs. sporadic FTD separately, but also broaden approaches by carefully designing studies to test for modifiers and the polygenic nature of Mendelian cases, provided adequate power.

Our study, however, gives grounds to the inference that FTD may be part of a wider spectrum of genetically mediated neurodegenerative conditions[67] spanning from ALS to AD phenotypes and encompassing PSP, PD, and CBD. The current study also lays the foundations for future work aimed at further characterizing population-specific features of potential FTD-discriminant *APOE* haplotype(s) and the functional involvement and contribution of the *MAPT* H1c haplotype and *RPSA-MOBP* loci to pathogenesis in brain cortex of sporadic forms of FTD.

Data and code availability

The accession number for the summary statistics (available for download from UCL Research Data Repository) is https://doi.org/10.5522/04/25600692.v1. Underlying participant-level data are available to potential collaborators, where individual-study data-access consent and pre-approved ethics are obtained to permit such data sharing: directly contact the site PIs (Note S2) to obtain both permission and consent to access their samples' data.

Supplemental information

Supplemental information can be found online at https://doi.org/10.1016/j.ajhg.2024.05.017.

Acknowledgments

This work was funded by the Alzheimer's Society (grant number 284 to R.F.). Additional funding info and acknowledgments can be found in the supplemental information.

Author contributions

C.M.—whole-data analyses, data generation, writing of the paper; D.A.K.—whole-data analyses, data generation, revision of the paper; R.F.—funding, project design, genotyping, analyses, data generation, initial drafting of the paper; G.L.—analysis support; B.C.—curation of metadata, revision of the paper; V.S.—curation

Table 4. Top marker summary statistics for the Central European/Nordic and Mediterranean European cohorts

						Discovery										Replication						Joint Analysis			
						Minor allele frequency						PLINK				Minor allele frequency		PLINK				METAL			
Cohort	rs number	Chr	Base pair	Locus	A1/A2	Controls	Cases	1000G IBR	1000G TSI	1000G GBR	1000G CEU	OR	Beta	SE	*p* value	Controls	Cases	OR	Beta	SE	*p* value	Beta	OR	SE	*p* value
Central European/Nordic	rs199443	17	44,819,565	*MAPT*	T/C	0.215	0.175	-	-	0.231	0.197	1.293	0.256	0.045	1.08E-08	0.211	0.179	1.283	0.249	0.109	2.19E-02	0.256	1.291	0.042	8.018E-10
	rs6857	19	45,392,254	*APOE*	T/C	0.165	0.186	-	-	0.170	0.197	1.193	0.176	0.045	7.90E-05	0.168	0.203	1.235	0.211	0.095	2.55E-02	0.183	1.201	0.041	6.799E-06
Mediterranean European	rs199443	17	44,819,565	*MAPT*	T/C	0.250	0.207	0.262	0.308	-	-	1.184	0.170	0.070	1.52E-02	0.265	0.234	1.142	0.133	0.108	2.19E-01	0.159	1.172	0.059	6.80E-03
	rs6857	19	45,392,254	*APOE*	T/C	0.109	0.169	0.164	0.117	-	-	1.457	0.376	0.082	4.00E-06	0.124	0.165	1.281	0.248	0.126	4.88E-02	0.338	1.402	0.068	7.67E-07

Discovery, replication, and joint analysis statistics are shown for the top markers (smallest *p* value) at the significant loci. 1000G, 1000 Genomes MAF; GBR, British in England and Scotland; CEU, Northern Europeans from Utah; IBR, Iberian populations in Spain; TSI, Tuscans from Italy.

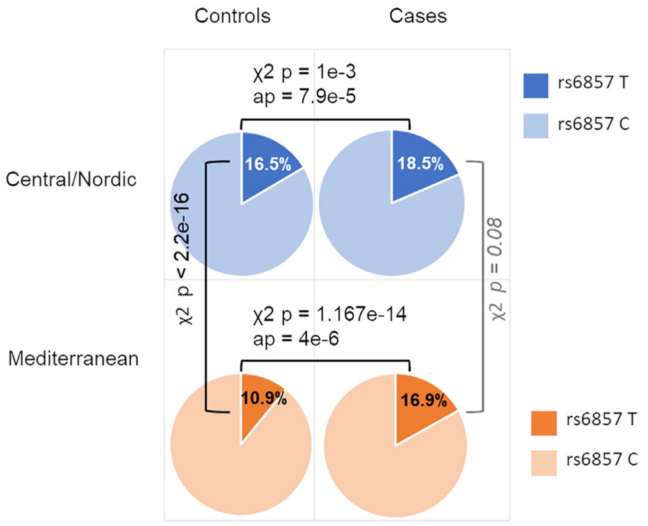

Figure 4. Risk allele frequencies of top marker (rs6857) at the chromosome 19 locus in the Central/Nordic and Mediterranean European discovery cohorts

Uncorrected p values calculated via χ^2 are reported; association p values (ap) are reported for the case-control comparisons.

of metadata, revision of the paper; E.J.—genotyping, revision of the paper; M.M.X.T.—genotyping, provision of control samples, revision of the paper; M.A.N.—previous analyses, data generation, project design, revision of the paper; P.M.—funding, project design, provision of control samples, revision of the paper; A.B.S.—funding, project design, revision of the paper; J.H.—funding, project design, revision of the paper; V.E.-P.—project design, supervision, writing of the paper; D.A., G.F., E.S., S.B.-C., S.W.S.—control collection and characterization, revision of the paper; M.K., G.K.M.—genotyping, revision of the paper; all other authors— sample collection and characterization, revision of the paper.

Declaration of interests

O.A.A. has received speakers' honoraria from Janssen, Lundbeck, and Sunovion and is a consultant to Cortechs.ai. C.C. received research support from GSK and EISAI. The funders of the study had no role in the collection, analysis, or interpretation of data; in the writing of the report; or in the decision to submit the paper for publication. C.C. is a member of the advisory board of Vivid Genomics and Circular Genomics. M.A.N. and H.L.L. hold part of a competitive contract awarded to Data Tecnica International LLC by the National Institutes of Health to support open science research. M.A.N. currently serves on the scientific advisory board for Character Bio Inc. and Neuron23 Inc. I.R.M. receives license royalties for patent related to PGRN therapy and is a member of the scientific advisory committee for Prevail Therapeutics. H.R.M. is employed by UCL. In the last 12 months he reports paid consultancy from Roche, Aprinoia, AI Therapeutics, and Amylyx; lecture fees/honoraria from BMJ, Kyowa Kirin, and Movement Disorders Society; and research grants from Parkinson's UK, Cure Parkinson's Trust, PSP Association, Medical Research Council, and the Michael J. Fox Foundation. H.R.M. is a co-applicant on a patent application related to C9ORF72—Method for diagnosing a neurodegenerative disease (PCT/GB2012/052140). R.P. has received honoraria for advisory boards

and speaker engagements from Roche, EISAI, Eli Lilly, Biogen, Janssen-Cilag, Astra Zeneca, Schwabe, Grifols, Novo Nordisk, and Tabuk. R.S.-V. served in advisory board meetings for Wave Life Sciences, Ionis, and Novo Nordisk; has received personal fees for participating in educational activities from Janssen, Roche Diagnostics, and Neuraxpharm; and has received funding to her institution for research projects from Biogen and Sage Pharmaceuticals. S.W.S. received research support from Cerevel Therapeutics and is a member of the scientific advisory board of the Lewy Body Dementia Association and the Multiple System Atrophy Coalition. J.S.Y. serves on the scientific advisory board for the Epstein Family Alzheimer's Research Collaboration. J.H. does consulting and gives talks for Eli-Lilly, Roche, and Eisai and is on the Ceracuity advisory board.

Received: January 2, 2024
Accepted: May 17, 2024
Published: June 17, 2024

References

1. Rabinovici, G.D., and Miller, B.L. (2010). Frontotemporal lobar degeneration: epidemiology, pathophysiology, diagnosis and management. CNS Drugs *24*, 375–398.
2. Ratnavalli, E., Brayne, C., Dawson, K., and Hodges, J.R. (2002). The prevalence of frontotemporal dementia. Neurology *58*, 1615–1621.
3. Neary, D., Snowden, J.S., Gustafson, L., Passant, U., Stuss, D., Black, S., Freedman, M., Kertesz, A., Robert, P.H., Albert, M., et al. (1998). Frontotemporal lobar degeneration: a consensus on clinical diagnostic criteria. Neurology *51*, 1546–1554.
4. Rascovsky, K., Hodges, J.R., Knopman, D., Mendez, M.F., Kramer, J.H., Neuhaus, J., van Swieten, J.C., Seelaar, H., Dopper, E.G.P., Onyike, C.U., et al. (2011). Sensitivity of revised diagnostic criteria for the behavioural variant of frontotemporal dementia. Brain *134*, 2456–2477.
5. Gorno-Tempini, M.L., Hillis, A.E., Weintraub, S., Kertesz, A., Mendez, M., Cappa, S.F., Ogar, J.M., Rohrer, J.D., Black, S., Boeve, B.F., et al. (2011). Classification of primary progressive aphasia and its variants. Neurology *76*, 1006–1014.
6. Rohrer, J.D., and Warren, J.D. (2011). Phenotypic signatures of genetic frontotemporal dementia. Curr. Opin. Neurol. *24*, 542–549.
7. Murley, A.G., Coyle-Gilchrist, I., Rouse, M.A., Jones, P.S., Li, W., Wiggins, J., Lansdall, C., Rodríguez, P.V., Wilcox, A., Tsvetanov, K.A., et al. (2020). Redefining the multidimensional clinical phenotypes of frontotemporal lobar degeneration syndromes. Brain *143*, 1555–1571.
8. Halliday, G., Bigio, E.H., Cairns, N.J., Neumann, M., Mackenzie, I.R.A., and Mann, D.M.A. (2012). Mechanisms of disease in frontotemporal lobar degeneration: gain of function versus loss of function effects. Acta Neuropathol. *124*, 373–382.
9. DeJesus-Hernandez, M., Mackenzie, I.R., Boeve, B.F., Boxer, A.L., Baker, M., Rutherford, N.J., Nicholson, A.M., Finch, N.A., Flynn, H., Adamson, J., et al. (2011). Expanded GGGGCC hexanucleotide repeat in noncoding region of C9ORF72 causes chromosome 9p-linked FTD and ALS. Neuron *72*, 245–256.
10. van der Zee, J., Gijselinck, I., Dillen, L., Van Langenhove, T., Theuns, J., Engelborghs, S., Philtjens, S., Vandenbulcke, M., Sleegers, K., Sieben, A., et al. (2013). A pan-European study of the C9orf72 repeat associated with FTLD: geographic prevalence, genomic instability, and intermediate repeats. Hum. Mutat. *34*, 363–373.
11. Van Deerlin, V.M., Sleiman, P.M.A., Martinez-Lage, M., Chen-Plotkin, A., Wang, L.S., Graff-Radford, N.R., Dickson, D.W., Rademakers, R., Boeve, B.F., Grossman, M., et al. (2010). Common variants at 7p21 are associated with frontotemporal lobar degeneration with TDP-43 inclusions. Nat. Genet. *42*, 234–239.
12. Pottier, C., Zhou, X., Perkerson, R.B., 3rd, Baker, M., Jenkins, G.D., Serie, D.J., Ghidoni, R., Benussi, L., Binetti, G., López de Munain, A., et al. (2018). Potential genetic modifiers of disease risk and age at onset in patients with frontotemporal lobar degeneration and GRN mutations: a genome-wide association study. Lancet Neurol. *17*, 548–558.
13. Pottier, C., Ren, Y., Perkerson, R.B., 3rd, Baker, M., Jenkins, G.D., van Blitterswijk, M., DeJesus-Hernandez, M., van Rooij, J.G.J., Murray, M.E., Christopher, E., et al. (2019). Genome-wide analyses as part of the international FTLD-TDP whole-genome sequencing consortium reveals novel disease risk factors and increases support for immune dysfunction in FTLD. Acta Neuropathol. *137*, 879–899.
14. Ferrari, R., Hernandez, D.G., Nalls, M.A., Rohrer, J.D., Ramasamy, A., Kwok, J.B.J., Dobson-Stone, C., Brooks, W.S., Schofield, P.R., Halliday, G.M., et al. (2014). Frontotemporal dementia and its subtypes: a genome-wide association study. Lancet Neurol. *13*, 686–699.
15. Eichler, E.E., Flint, J., Gibson, G., Kong, A., Leal, S.M., Moore, J.H., and Nadeau, J.H. (2010). Missing heritability and strategies for finding the underlying causes of complex disease. Nat. Rev. Genet. *11*, 446–450.
16. Ferrari, R., Manzoni, C., and Hardy, J. (2019). Genetics and molecular mechanisms of frontotemporal lobar degeneration: an update and future avenues. Neurobiol. Aging *78*, 98–110.
17. Strong, M.J., Abrahams, S., Goldstein, L.H., Woolley, S., McLaughlin, P., Snowden, J., Mioshi, E., Roberts-South, A., Benatar, M., HortobáGyi, T., et al. (2017). Amyotrophic lateral sclerosis - frontotemporal spectrum disorder (ALS-FTSD): Revised diagnostic criteria. Amyotroph. Lateral Scler. Frontotemporal Degener. *18*, 153–174.
18. Nalls, M.A., Bras, J., Hernandez, D.G., Keller, M.F., Majounie, E., Renton, A.E., Saad, M., Jansen, I., Guerreiro, R., Lubbe, S., et al. (2015). NeuroX, a fast and efficient genotyping platform for investigation of neurodegenerative diseases. Neurobiol. Aging *36*. 1605 e7-e1605.e1.605E12.
19. Blauwendraat, C., Faghri, F., Pihlstrom, L., Geiger, J.T., Elbaz, A., Lesage, S., Corvol, J.C., May, P., Nicolas, A., Abramzon, Y., et al. (2017). NeuroChip, an updated version of the NeuroX genotyping platform to rapidly screen for variants associated with neurological diseases. Neurobiol. Aging *57*, 247.e9–247.e13.
20. Graffelman, J., and Moreno, V. (2013). The mid p-value in exact tests for Hardy-Weinberg equilibrium. Stat. Appl. Genet. Mol. Biol. *12*, 433–448.
21. Lin, D.Y., and Sullivan, P.F. (2009). Meta-analysis of genome-wide association studies with overlapping subjects. Am. J. Hum. Genet. *85*, 862–872.
22. Willer, C.J., Li, Y., and Abecasis, G.R. (2010). METAL: fast and efficient meta-analysis of genomewide association scans. Bioinformatics *26*, 2190–2191.
23. Manolio, T.A., Collins, F.S., Cox, N.J., Goldstein, D.B., Hindorff, L.A., Hunter, D.J., McCarthy, M.I., Ramos, E.M., Cardon, L.R., Chakravarti, A., et al. (2009). Finding the missing heritability of complex diseases. Nature *461*, 747–753.

24. Bulik-Sullivan, B., Finucane, H.K., Anttila, V., Gusev, A., Day, F.R., Loh, P.R., ReproGen Consortium; Psychiatric Genomics Consortium; and Genetic Consortium for Anorexia Nervosa of the Wellcome Trust Case Control Consortium 3, and Duncan, L., et al. (2015). An atlas of genetic correlations across human diseases and traits. Nat. Genet. *47*, 1236–1241.
25. Kunkle, B.W., Grenier-Boley, B., Sims, R., Bis, J.C., Damotte, V., Naj, A.C., Boland, A., Vronskaya, M., van der Lee, S.J., Amlie-Wolf, A., et al. (2019). Author Correction: Genetic meta-analysis of diagnosed Alzheimer's disease identifies new risk loci and implicates Aβ, tau, immunity and lipid processing. Nat. Genet. *51*, 1423–1424.
26. Bellenguez, C., Küçükali, F., Jansen, I.E., Kleineidam, L., Moreno-Grau, S., Amin, N., Naj, A.C., Campos-Martin, R., Grenier-Boley, B., Andrade, V., et al. (2022). New insights into the genetic etiology of Alzheimer's disease and related dementias. Nat. Genet. *54*, 412–436.
27. Nalls, M.A., Blauwendraat, C., Vallerga, C.L., Heilbron, K., Bandres-Ciga, S., Chang, D., Tan, M., Kia, D.A., Noyce, A.J., Xue, A., et al. (2019). Identification of novel risk loci, causal insights, and heritable risk for Parkinson's disease: a meta-analysis of genome-wide association studies. Lancet Neurol. *18*, 1091–1102.
28. van Rheenen, W., van der Spek, R.A.A., Bakker, M.K., van Vugt, J.J.F.A., Hop, P.J., Zwamborn, R.A.J., de Klein, N., Westra, H.J., Bakker, O.B., Deelen, P., et al. (2021). Common and rare variant association analyses in amyotrophic lateral sclerosis identify 15 risk loci with distinct genetic architectures and neuron-specific biology. Nat. Genet. *53*, 1636–1648.
29. Chia, R., Sabir, M.S., Bandres-Ciga, S., Saez-Atienzar, S., Reynolds, R.H., Gustavsson, E., Walton, R.L., Ahmed, S., Viollet, C., Ding, J., et al. (2021). Genome sequencing analysis identifies new loci associated with Lewy body dementia and provides insights into its genetic architecture. Nat. Genet. *53*, 294–303.
30. Han, B., Pouget, J.G., Slowikowski, K., Stahl, E., Lee, C.H., Diogo, D., Hu, X., Park, Y.R., Kim, E., Gregersen, P.K., et al. (2016). A method to decipher pleiotropy by detecting underlying heterogeneity driven by hidden subgroups applied to autoimmune and neuropsychiatric diseases. Nat. Genet. *48*, 803–810.
31. Caffrey, T.M., Joachim, C., Paracchini, S., Esiri, M.M., and Wade-Martins, R. (2006). Haplotype-specific expression of exon 10 at the human MAPT locus. Hum. Mol. Genet. *15*, 3529–3537.
32. Valenca, G.T., Srivastava, G.P., Oliveira-Filho, J., White, C.C., Yu, L., Schneider, J.A., Buchman, A.S., Shulman, J.M., Bennett, D.A., and De Jager, P.L. (2016). The Role of MAPT Haplotype H2 and Isoform 1N/4R in Parkinsonism of Older Adults. PLoS One *11*, e0157452.
33. Watanabe, K., Taskesen, E., van Bochoven, A., and Posthuma, D. (2017). Functional mapping and annotation of genetic associations with FUMA. Nat. Commun. *8*, 1826.
34. DiGiacomo, V., and Meruelo, D. (2016). Looking into laminin receptor: critical discussion regarding the non-integrin 37/67-kDa laminin receptor/RPSA protein. Biol. Rev. Camb. Philos. Soc. *91*, 288–310.
35. Nicolas, A., Kenna, K.P., Renton, A.E., Ticozzi, N., Faghri, F., Chia, R., Dominov, J.A., Kenna, B.J., Nalls, M.A., Keagle, P., et al. (2018). Genome-wide Analyses Identify KIF5A as a Novel ALS Gene. Neuron *97*, 1267–1288.
36. Lambert, J.C., Ibrahim-Verbaas, C.A., Harold, D., Naj, A.C., Sims, R., Bellenguez, C., DeStafano, A.L., Bis, J.C., Beecham, G.W., Grenier-Boley, B., et al. (2013). Meta-analysis of 74,046 individuals identifies 11 new susceptibility loci for Alzheimer's disease. Nat. Genet. *45*, 1452–1458.
37. Naj, A.C., Jun, G., Beecham, G.W., Wang, L.S., Vardarajan, B.N., Buros, J., Gallins, P.J., Buxbaum, J.D., Jarvik, G.P., Crane, P.K., et al. (2011). Common variants at MS4A4/MS4A6E, CD2AP, CD33 and EPHA1 are associated with late-onset Alzheimer's disease. Nat. Genet. *43*, 436–441.
38. de Rojas, I., Moreno-Grau, S., Tesi, N., Grenier-Boley, B., Andrade, V., Jansen, I.E., Pedersen, N.L., Stringa, N., Zettergren, A., Hernández, I., et al. (2021). Common variants in Alzheimer's disease and risk stratification by polygenic risk scores. Nat. Commun. *12*, 3417.
39. Höglinger, G.U., Melhem, N.M., Dickson, D.W., Sleiman, P.M., Wang, L.S., Klei, L., Rademakers, R., de Silva, R., Litvan, I., Riley, D.E., et al. (2011). Identification of common variants influencing risk of the tauopathy progressive supranuclear palsy. Nat. Genet. *43*, 699–705.
40. Kouri, N., Ross, O.A., Dombroski, B., Younkin, C.S., Serie, D.J., Soto-Ortolaza, A., Baker, M., Finch, N.C.A., Yoon, H., Kim, J., et al. (2015). Genome-wide association study of corticobasal degeneration identifies risk variants shared with progressive supranuclear palsy. Nat. Commun. *6*, 7247.
41. Sanchez-Contreras, M.Y., Kouri, N., Cook, C.N., Serie, D.J., Heckman, M.G., Finch, N.A., Caselli, R.J., Uitti, R.J., Wszolek, Z.K., Graff-Radford, N., et al. (2018). Replication of progressive supranuclear palsy genome-wide association study identifies SLCO1A2 and DUSP10 as new susceptibility loci. Mol. Neurodegener. *13*, 37.
42. Chang, D., Nalls, M.A., Hallgrímsdóttir, I.B., Hunkapiller, J., van der Brug, M., Cai, F., International Parkinson's Disease Genomics Consortium; and 23andMe Research Team, Kerchner, G.A., Ayalon, G., et al. (2017). A meta-analysis of genome-wide association studies identifies 17 new Parkinson's disease risk loci. Nat. Genet. *49*, 1511–1516.
43. Reus, L.M., Pasaniuc, B., Posthuma, D., Boltz, T., International FTD-Genomics Consortium, Pijnenburg, Y.A.L., and Ophoff, R.A. (2021). Gene Expression Imputation Across Multiple Tissue Types Provides Insight Into the Genetic Architecture of Frontotemporal Dementia and Its Clinical Subtypes. Biol. Psychiatry *89*, 825–835.
44. Hughes, A., Mann, D., and Pickering-Brown, S. (2003). Tau haplotype frequency in frontotemporal lobar degeneration and amyotrophic lateral sclerosis. Exp. Neurol. *181*, 12–16.
45. Verpillat, P., Camuzat, A., Hannequin, D., Thomas-Anterion, C., Puel, M., Belliard, S., Dubois, B., Didic, M., Michel, B.F., Lacomblez, L., et al. (2002). Association between the extended tau haplotype and frontotemporal dementia. Arch. Neurol. *59*, 935–939.
46. Pastor, P., Moreno, F., Clarimón, J., Ruiz, A., Combarros, O., Calero, M., López de Munain, A., Bullido, M.J., de Pancorbo, M.M., Carro, E., et al. (2016). MAPT H1 Haplotype is Associated with Late-Onset Alzheimer's Disease Risk in APOEϵ4 Noncarriers: Results from the Dementia Genetics Spanish Consortium. J. Alzheimers Dis. *49*, 343–352.
47. Sánchez-Juan, P., Moreno, S., de Rojas, I., Hernández, I., Valero, S., Alegret, M., Montrreal, L., García González, P., Lage, C., López-García, S., et al. (2019). The MAPT H1 Haplotype Is a Risk Factor for Alzheimer's Disease in APOE epsilon4 Non-carriers. Front. Aging Neurosci. *11*, 327.
48. Houlden, H., Baker, M., Morris, H.R., MacDonald, N., Pickering–Brown, S., Adamson, J., Lees, A., Rossor, M., Quinn, N.,

Kertesz, A., et al. (2001). Corticobasal degeneration and progressive supranuclear palsy share a common tau haplotype. Neurology *56*, 1702–1706.
49. Pittman, A.M., Myers, A.J., Abou-Sleiman, P., Fung, H.C., Kaleem, M., Marlowe, L., Duckworth, J., Leung, D., Williams, D., Kilford, L., et al. (2005). Linkage disequilibrium fine mapping and haplotype association analysis of the tau gene in progressive supranuclear palsy and corticobasal degeneration. J. Med. Genet. *42*, 837–846.
50. Yokoyama, J.S., Karch, C.M., Fan, C.C., Bonham, L.W., Kouri, N., Ross, O.A., Rademakers, R., Kim, J., Wang, Y., Höglinger, G.U., et al. (2017). Shared genetic risk between corticobasal degeneration, progressive supranuclear palsy, and frontotemporal dementia. Acta Neuropathol. *133*, 825–837.
51. Healy, D.G., Abou-Sleiman, P.M., Lees, A.J., Casas, J.P., Quinn, N., Bhatia, K., Hingorani, A.D., and Wood, N.W. (2004). Tau gene and Parkinson's disease: a case-control study and meta-analysis. J. Neurol. Neurosurg. Psychiatry *75*, 962–965.
52. Ferrari, R., Wang, Y., Vandrovcova, J., Guelfi, S., Witeolar, A., Karch, C.M., Schork, A.J., Fan, C.C., Brewer, J.B.; and International FTD-Genomics Consortium IFGC (2017). Genetic architecture of sporadic frontotemporal dementia and overlap with Alzheimer's and Parkinson's diseases. J. Neurol. Neurosurg. Psychiatry *88*, 152–164.
53. Gustafson, D.R., Mazzuco, S., Ongaro, F., Antuono, P., Forloni, G., Albani, D., Gajo, G.B., Durante, E., Caberlotto, L., Zanardo, A., et al. (2012). Body mass index, cognition, disability, APOE genotype, and mortality: the "Treviso Longeva" Study. Am. J. Geriatr. Psychiatry *20*, 594–602.
54. Corbo, R.M., and Scacchi, R. (1999). Apolipoprotein E (APOE) allele distribution in the world. Is APOE*4 a 'thrifty' allele? Ann. Hum. Genet. *63*, 301–310.
55. Moreno-Grau, S., de Rojas, I., Hernández, I., Quintela, I., Montrreal, L., Alegret, M., Hernández-Olasagarre, B., Madrid, L., González-Perez, A., Maroñas, O., et al. (2019). Genome-wide association analysis of dementia and its clinical endophenotypes reveal novel loci associated with Alzheimer's disease and three causality networks: The GR@ACE project. Alzheimers Dement. *15*, 1333–1347.
56. Ossenkoppele, R., Pijnenburg, Y.A.L., Perry, D.C., Cohn-Sheehy, B.I., Scheltens, N.M.E., Vogel, J.W., Kramer, J.H., van der Vlies, A.E., La Joie, R., Rosen, H.J., et al. (2015). The behavioural/dysexecutive variant of Alzheimer's disease: clinical, neuroimaging and pathological features. Brain *138*, 2732–2749.
57. Ossenkoppele, R., Singleton, E.H., Groot, C., Dijkstra, A.A., Eikelboom, W.S., Seeley, W.W., Miller, B., Laforce, R.J., Scheltens, P., Papma, J.M., et al. (2022). Research Criteria for the Behavioral Variant of Alzheimer Disease: A Systematic Review and Meta-analysis. JAMA Neurol. *79*, 48–60.
58. Joshua Stevenson-Hoare, A.-K.S., Sandor, C., Hardy, J., and Escott-Price, V. (2023). New cases of dementia are rising in elderly populations in Wales, UK. J. Neurol. Sci.
59. Naasan, G., Rabinovici, G.D., Ghosh, P., Elofson, J.D., Miller, B.L., Coppola, G., Karydas, A., Fong, J., Perry, D., Lee, S.E., et al. (2016). Amyloid in dementia associated with familial FTLD: not an innocent bystander. Neurocase *22*, 76–83.
60. Rabinovici, G.D., Carrillo, M.C., Forman, M., DeSanti, S., Miller, D.S., Kozauer, N., Petersen, R.C., Randolph, C., Knopman, D.S., Smith, E.E., et al. (2017). Multiple comorbid neuropathologies in the setting of Alzheimer's disease neuropathology and implications for drug development. Alzheimers Dement. *3*, 83–91.
61. Ferrari, R., Grassi, M., Salvi, E., Borroni, B., Palluzzi, F., Pepe, D., D'Avila, F., Padovani, A., Archetti, S., Rainero, I., et al. (2015). A genome-wide screening and SNPs-to-genes approach to identify novel genetic risk factors associated with frontotemporal dementia. Neurobiol. Aging *36*. 2904 e13-e2904.e2.904E26.
62. Ingannato, A., Bagnoli, S., Bessi, V., Ferrari, C., Mazzeo, S., Sorbi, S., and Nacmias, B. (2022). Intermediate alleles of HTT: A new pathway in longevity. J. Neurol. Sci. *438*, 120274.
63. Mishra, A., Ferrari, R., Heutink, P., Hardy, J., Pijnenburg, Y., Posthuma, D.; and International FTD-Genomics Consortium (2017). Gene-based association studies report genetic links for clinical subtypes of frontotemporal dementia. Brain *140*, 1437–1446.
64. Rubino, E., Vacca, A., Govone, F., De Martino, P., Pinessi, L., and Rainero, I. (2013). Apolipoprotein E polymorphisms in frontotemporal lobar degeneration: a meta-analysis. Alzheimers Dement. *9*, 706–713.
65. Malpetti, M., Cope, T.E., Street, D., Jones, P.S., Hezemans, F.H., Mak, E., Tsvetanov, K.A., Rittman, T., Bevan-Jones, W.R., Patterson, K., et al. (2023). Microglial activation in the frontal cortex predicts cognitive decline in frontotemporal dementia. Brain.
66. Grotzinger, A.D., Rhemtulla, M., de Vlaming, R., Ritchie, S.J., Mallard, T.T., Hill, W.D., Ip, H.F., Marioni, R.E., McIntosh, A.M., Deary, I.J., et al. (2019). Genomic structural equation modelling provides insights into the multivariate genetic architecture of complex traits. Nat. Hum. Behav. *3*, 513–525.
67. Koretsky, M.J., Alvarado, C., Makarious, M.B., Vitale, D., Levine, K., Bandres-Ciga, S., Dadu, A., Scholz, S.W., Sargent, L., Faghri, F., et al. (2023). Genetic risk factor clustering within and across neurodegenerative diseases. Brain.

The American Journal of Human Genetics, Volume *111*

Supplemental information

Genome-wide analyses reveal a potential role for the *MAPT*, *MOBP*, and *APOE* loci in sporadic frontotemporal dementia

Claudia Manzoni, Demis A. Kia, Raffaele Ferrari, Ganna Leonenko, Beatrice Costa, Valentina Saba, Edwin Jabbari, Manuela MX. Tan, Diego Albani, Victoria Alvarez, Ignacio Alvarez, Ole A. Andreassen, Antonella Angiolillo, Andrea Arighi, Matt Baker, Luisa Benussi, Valentina Bessi, Giuliano Binetti, Daniel J. Blackburn, Merce Boada, Bradley F. Boeve, Sergi Borrego-Ecija, Barbara Borroni, Geir Bråthen, William S. Brooks, Amalia C. Bruni, Paola Caroppo, Sara Bandres-Ciga, Jordi Clarimon, Rosanna Colao, Carlos Cruchaga, Adrian Danek, Sterre CM. de Boer, Itziar de Rojas, Alfonso di Costanzo, Dennis W. Dickson, Janine Diehl-Schmid, Carol Dobson-Stone, Oriol Dols-Icardo, Aldo Donizetti, Elise Dopper, Elisabetta Durante, Camilla Ferrari, Gianluigi Forloni, Francesca Frangipane, Laura Fratiglioni, Milica G. Kramberger, Daniela Galimberti, Maurizio Gallucci, Pablo García-González, Roberta Ghidoni, Giorgio Giaccone, Caroline Graff, Neill R. Graff-Radford, Jordan Grafman, Glenda M. Halliday, Dena G. Hernandez, Lena E. Hjermind, John R. Hodges, Guy Holloway, Edward D. Huey, Ignacio Illán-Gala, Keith A. Josephs, David S. Knopman, Mark Kristiansen, John B. Kwok, Isabelle Leber, Hampton L. Leonard, Ilenia Libri, Alberto Lleo, Ian R. Mackenzie, Gaganjit K. Madhan, Raffaele Maletta, Marta Marquié, Ales Maver, Manuel Menendez-Gonzalez, Graziella Milan, Bruce L. Miller, Christopher M. Morris, Huw R. Morris, Benedetta Nacmias, Judith Newton, Jørgen E. Nielsen, Christer Nilsson, Valeria Novelli, Alessandro Padovani, Suvankar Pal, Florence Pasquier, Pau Pastor, Robert Perneczky, Borut Peterlin, Ronald C. Petersen, Olivier Piguet, Yolande AL. Pijnenburg, Annibale A. Puca, Rosa Rademakers, Innocenzo Rainero, Lianne M. Reus, Anna MT. Richardson, Matthias Riemenschneider, Ekaterina Rogaeva, Boris Rogelj, Sara Rollinson, Howard Rosen, Giacomina Rossi, James B. Rowe, Elisa Rubino, Agustin Ruiz, Erika Salvi, Raquel Sanchez-Valle, Sigrid Botne Sando, Alexander F. Santillo, Jennifer A. Saxon, Johannes CM. Schlachetzki, Sonja W. Scholz, Harro Seelaar, William W. Seeley, Maria Serpente, Sandro Sorbi, Sabrina Sordon, Peter St George-Hyslop, Jennifer C. Thompson, Christine Van Broeckhoven, Vivianna M. Van Deerlin, Sven J. Van der Lee, John Van Swieten, Fabrizio Tagliavini, Julie van der Zee, Arianna Veronesi, Emilia Vitale, Maria Landqvist Waldo, Jennifer S. Yokoyama, Mike A. Nalls, Parastoo Momeni, Andrew B. Singleton, John Hardy, and Valentina Escott-Price

Figure S1

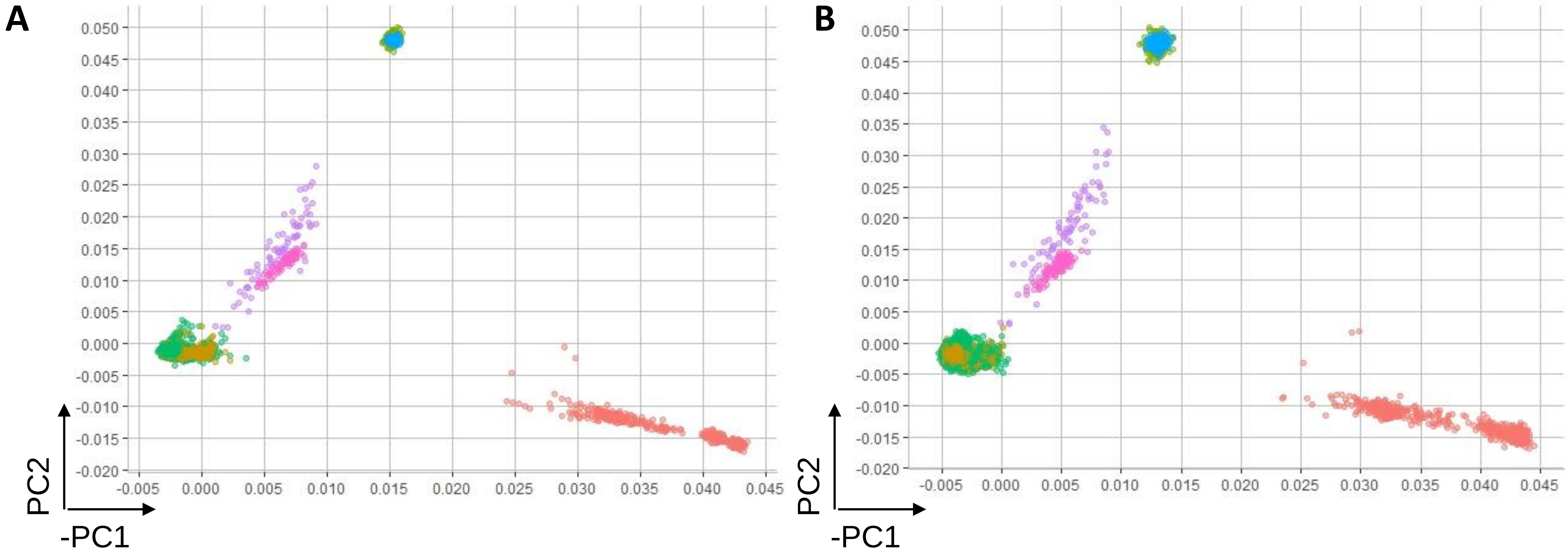

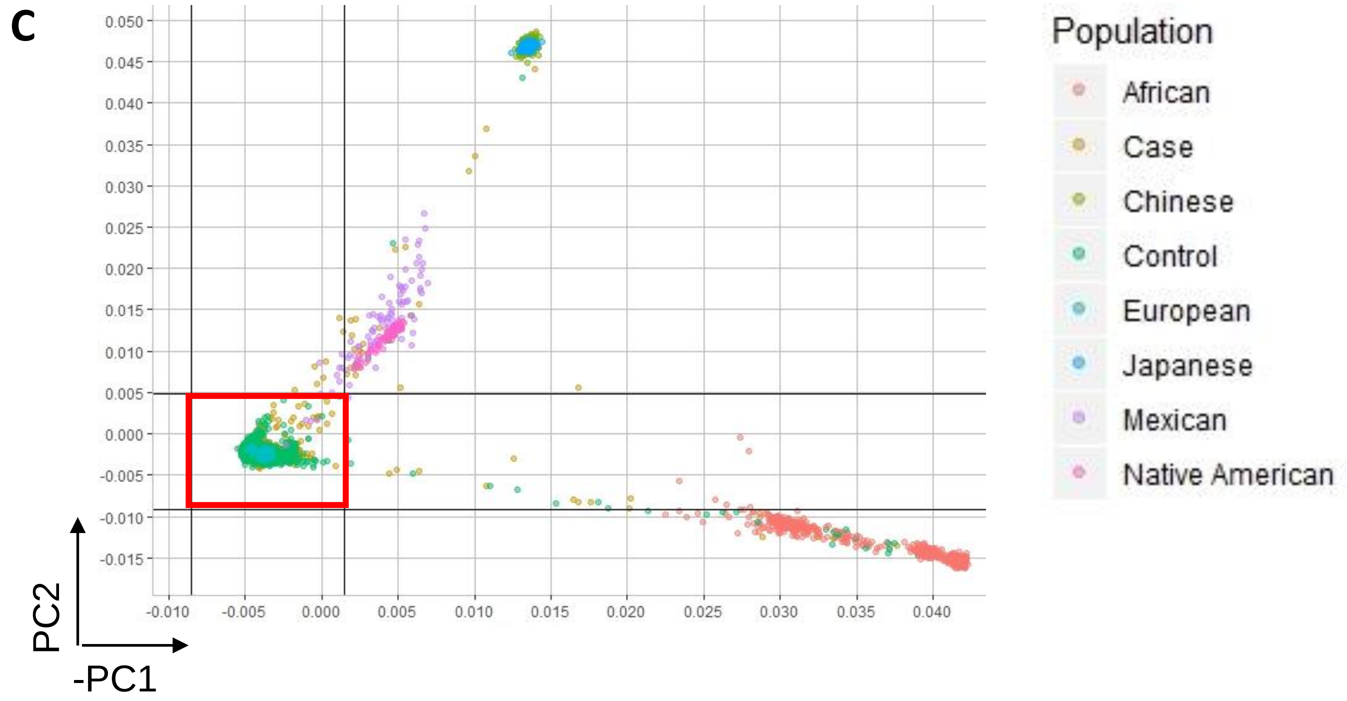

Figure S1 – *Principal component analysis on the 3 different cohorts. While cohorts I (A) and II (B) did not present outliers for population stratification with both cases and controls presenting with a relatively good overlap with European samples from HapMap, cohort-III (C) presented with a number of outliers that were removed by keeping only the samples (cases and controls) contained in the red square. We labelled outliers of the population with European ancestry individuals with > (or <) 1.5 standard deviation in component vector 1 and > 2.5 (or <) standard deviation for component vector 2*

Figure S2

A

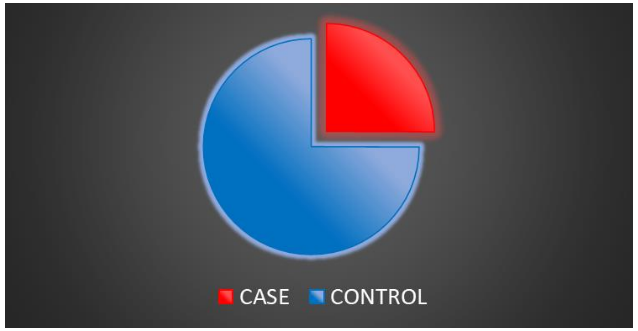

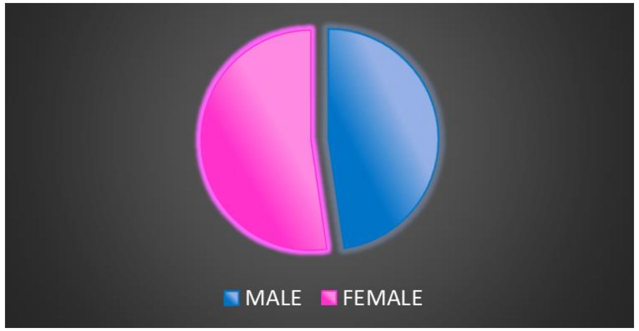

CASES: 3,756
CONTROLS: 11,233

MALES: 7,147
FEMALES: 7,842

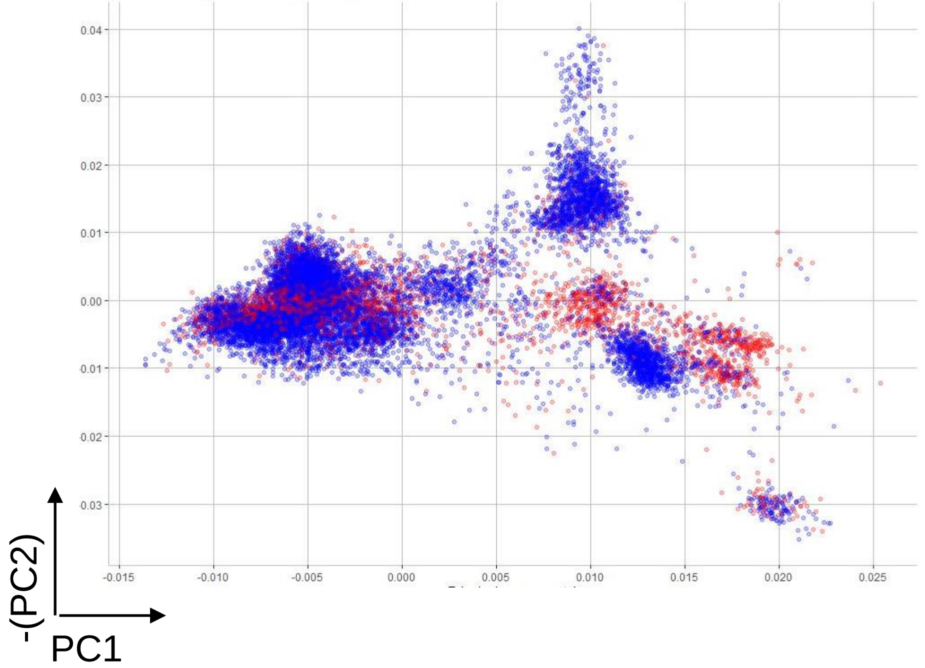

B

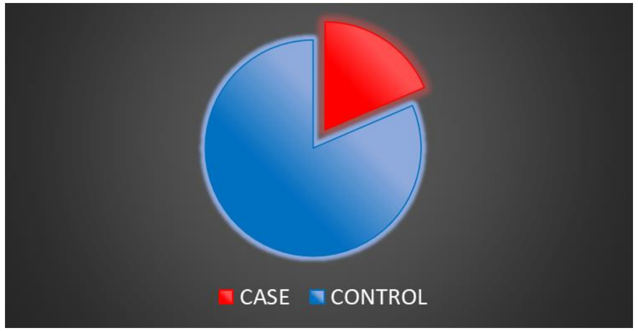

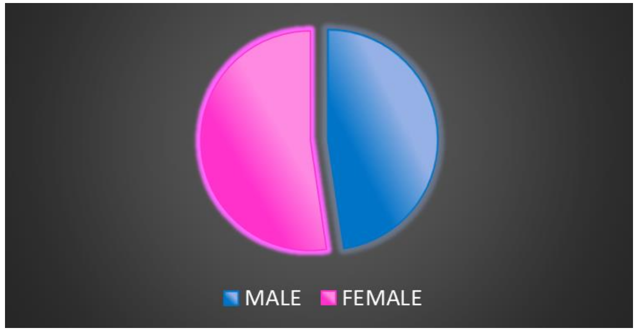

CASES: 929
CONTROLS: 4,075

MALES: 2,385
FEMALES: 2,619

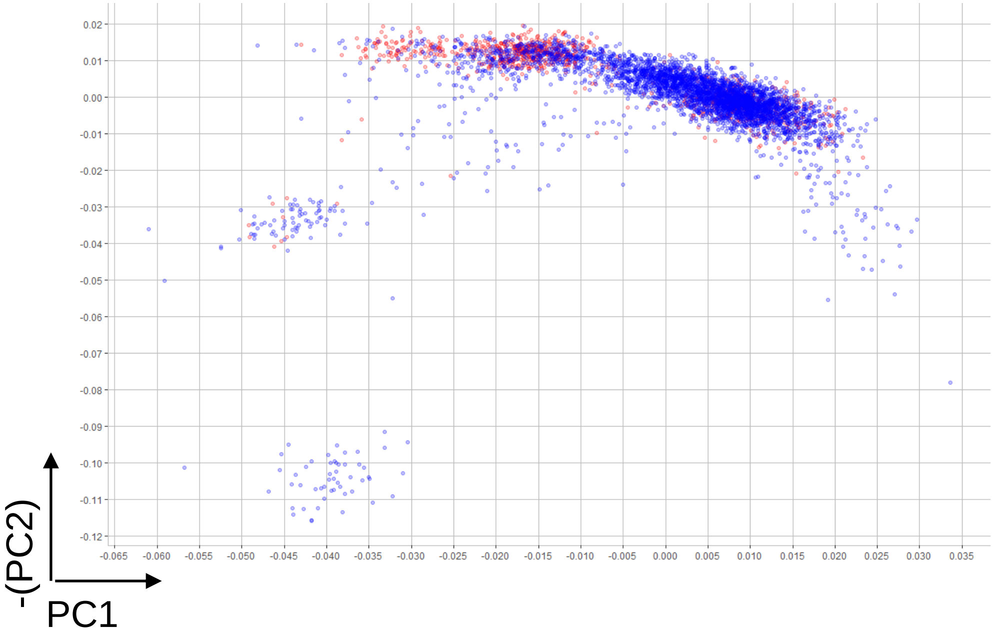

Figure S2 – *features of the discovery (A) and replication (B) cohorts. Division case/control and male/female; PC1 and PC2 were plotted and samples color-coded based on case/control status to verify similar population stratifications in cases in controls*

Figure S3

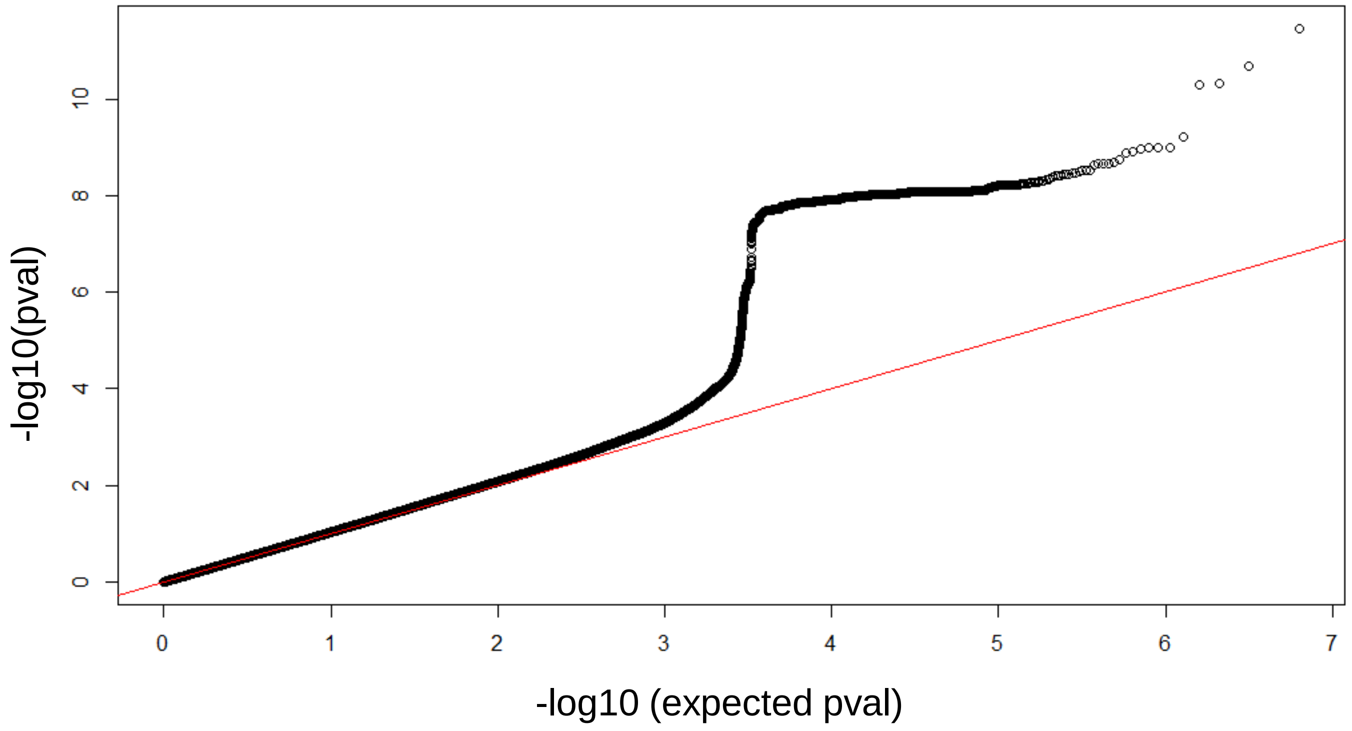

Figure S3 – *QQ plot of discovery phase*

Figure S4

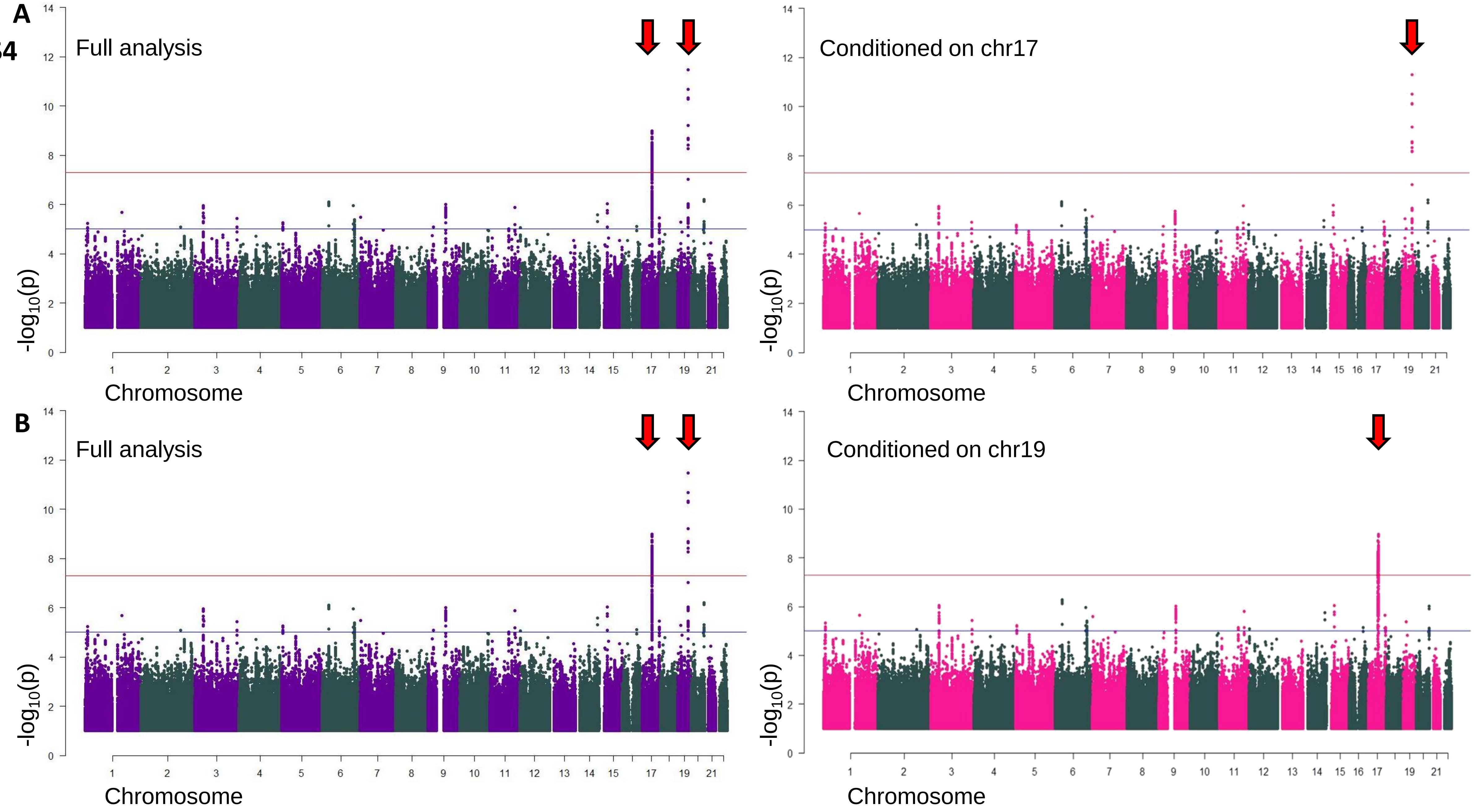

***Figure S4 –** Manhattan plot for discovery phase. Arrows indicate genome-wide significant towers (on chromosomes 17 and 19) A. Full analysis followed by analysis after conditioning for the top SNP on chromosome 17; as highlighted, the signal on chromosome 17 disappears. B. Full analysis followed by analysis after conditioning for the top SNP on chromosome 19; as highlighted, the signal on chromosome 19 disappears. The plot is cut at -$\log_{10}(p) = 1$.*

Figure S5

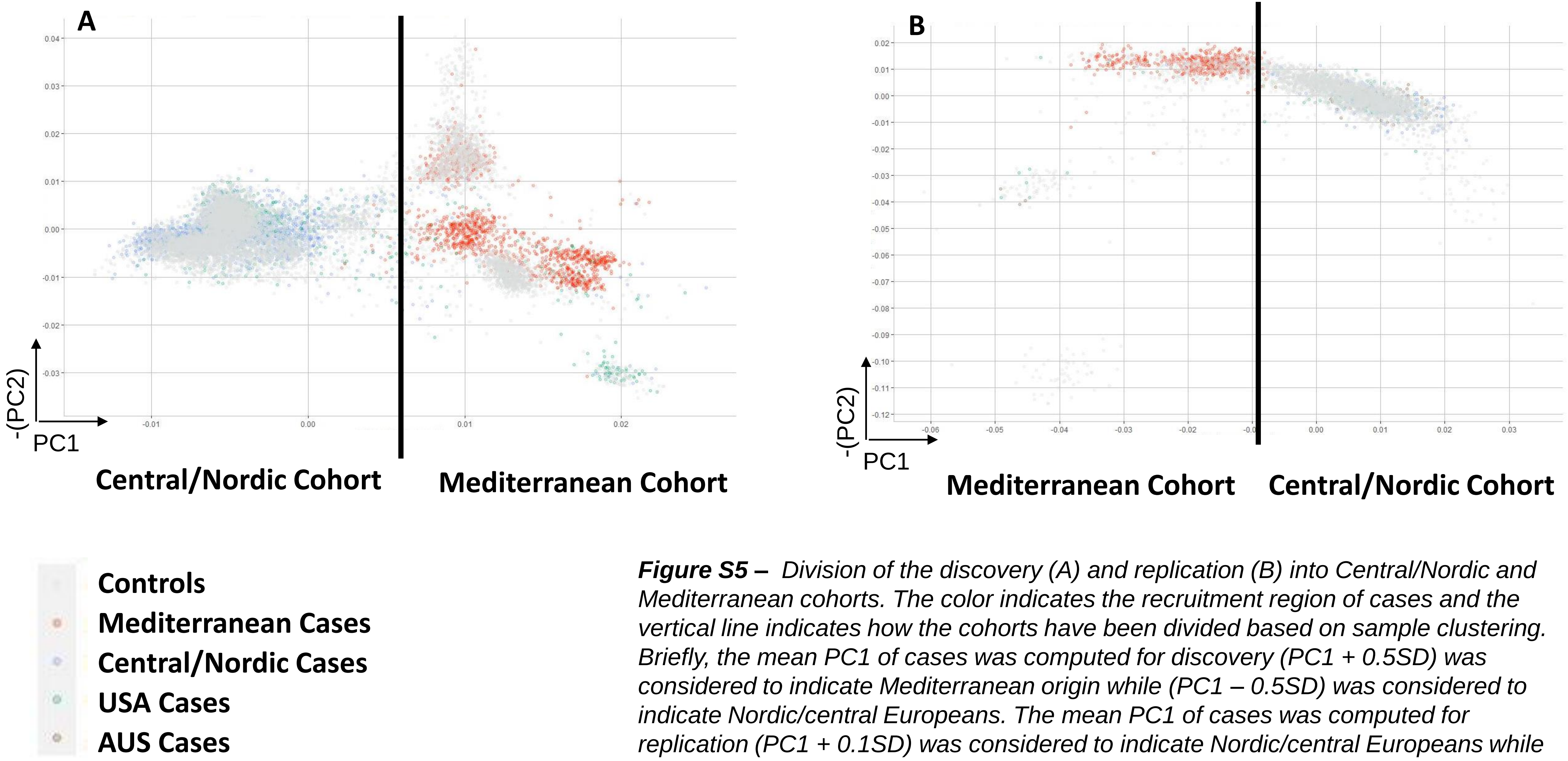

Figure S5 – *Division of the discovery (A) and replication (B) into Central/Nordic and Mediterranean cohorts. The color indicates the recruitment region of cases and the vertical line indicates how the cohorts have been divided based on sample clustering. Briefly, the mean PC1 of cases was computed for discovery (PC1 + 0.5SD) was considered to indicate Mediterranean origin while (PC1 – 0.5SD) was considered to indicate Nordic/central Europeans. The mean PC1 of cases was computed for replication (PC1 + 0.1SD) was considered to indicate Nordic/central Europeans while (PC1 – 0.1SD) was considered to indicate Mediterranean origin.*

Figure S6

A

Discovery Central/Nordic Discovery Mediterranean

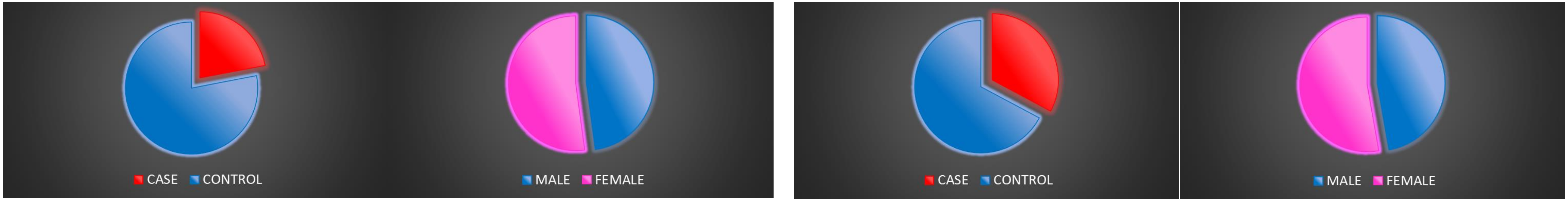

CASES: 2,359
CONTROLS: 8,371

MALES: 5,136
FEMALES: 5,594

CASES: 1,397
CONTROLS: 2,862

MALES: 2,011
FEMALES: 2,248

B

Replication Central/Nordic Replication Mediterranean

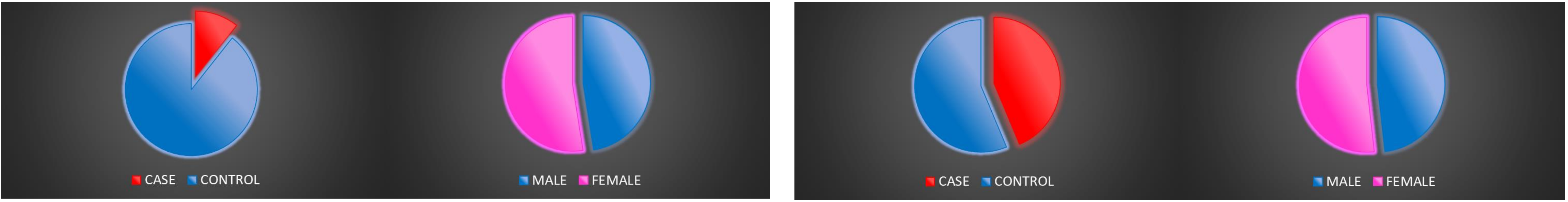

CASES: 405
CONTROLS: 3,400

MALES: 1,807
FEMALES: 1,998

CASES: 524
CONTROLS: 675

MALES: 578
FEMALES: 621

***Figure S6 –** features of the discovery (A) and replication (B) Central/Nordic and Mediterranean cohorts*

Figure S7

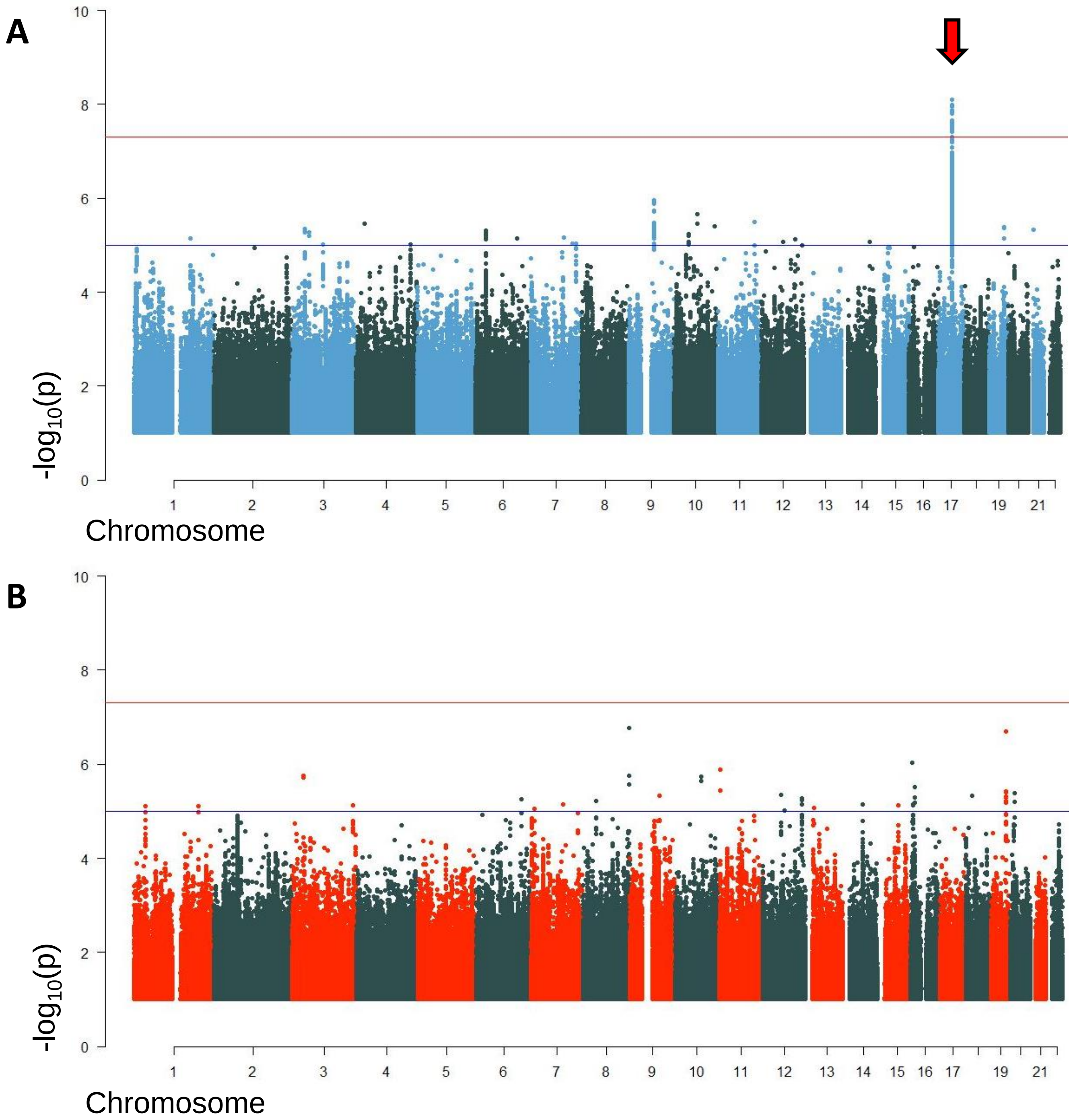

Figure S7 – Manhattan plot for discovery phase A. Central/Nordic and B. Mediterranean cohorts. Arrows indicate genome-wide significant tower (on chromosomes 17). The plot is cut at -log10(p) = 1.

Table S1

Collection Site	Institution	DISCOVERY			REPLICATION		
		Total	Sex		Total	Sex	
			Female	Male		Female	Male
Australia	University of Sydney - Neuroscience Research Australia	NA	NA	NA	93	59	34
Belgium	VIB-UAntwerp Center for Molecular Neurology	293	137	156	18	14	4
Canada	Vancouver General Hospital	80	44	36	10	6	4
	University of Toronto						
Denmark	Copenhagen University Hospital	6	5	1	NA	NA	NA
France	French Consortium	176	80	96	22	14	8
	Univeristy of Lille						
Germany	LMU Neurologische Klinik und Poliklinik	351	154	197	22	11	11
	Ludwig-Maximilians-University Munich						
	Technical University of Munich, School of Medicine						
	Universität des Saarlandes						
	University of Erlangen Nuremberg						
Italy	CNR Napoli	924	465	459	289	152	137
	IRCCS Istituto Centro San Giovanni di Dio Fatebenefratelli, Brescia						
	Istituto Neurologico C. Besta						
	Local Health Authority n.2 Marca Trevigiana, Treviso						
	Regional Neurogenetic Centre, ASPCZ, Lamezia Terme						
	University of Brescia						
	University of Florence						
	University of Milan						
	University of Salerno						
	University of Turin						
Netherlands	Erasmus Medical Center, Rotterdam	440	194	246	46	21	25
	VUMC, Amsterdam						
Norway	Institute of Clinical Medicine, University of Oslo	51	29	22	NA	NA	NA
	University Hospital of Trondheim						
Slovenia	Jožef Stefan Institute, Ljubljana	15	6	9	NA	NA	NA
Spain	Hospital Clínic of Barcelona	303	122	181	211	127	84
	Hospital de la Santa Creu i Sant Pau, Universitat Autònoma de Barcelona						
	Hospital Universitario Central de Asturias, Oviedo						
	Ace Alzheimer Center Barcelona						
	University Hospital Mutua de Terrassa, Terrassa, Barcelona						
Sweden	Karolinska Institutet	102	54	48	42	19	23
	Lund University						
	Skåne University Hospital, Malmö						
UK	Newcastle University	323	146	177	74	38	36
	Sheffield Institute for Translational Neuroscience (SITraN), University of Sheffield						
	University of Cambridge						
	University of Edinburgh						
	University of Manchester						
USA	Columbia University	692	325	367	102	48	54
	Mayo Clinic						
	UPENN						
	Northwestern, Chicago						
	UCSF						
	Washington University						
**Total**		**3756** (bvFTD: 2119; SD: 538; PNFA: 507; FTD-MND: 242; FTD-Unspecified: 350)	**1761**	**1995**	**929** (bvFTD: 445; SD: 113; PNFA: 151; FTD-MND: 58; FTD-Unspecified: 162)	**509**	**420**

Table S1 – Samples breakdown

Table S2 - *MAPT* and *APOE* loci significant markers

Table S3 - Association analysis using MAOS, A1 = minor allele, bold = risk allele.

Table S4 - Summary of suggestive markers taken forward for replication

**#**	**ALPHA**	**=**	**0.05**								
**#**	**NUMBER_OF_TESTS**	**=**	**15258**								
**#**	**_SET1_**	**VARIABLE**	**=**	**GO_bp:go_negative_regulation_of_steroid_metabolic_process**	**(set)**						
**#**	**_SET1_**	**NGENES**	**=**	**13**							
**#**	**_SET1_**	**P-VALUE**	**=**	**9.19E-08**							
**_SET1_**	**GENE**	**CHR**	**START**	**STOP**	**NSNPS**	**NPARAM**	**N**	**ZSTAT**	**P**	**ZFITTED_BASE**	**ZRESID_BASE**
SET1	ENSG00000117707	1	214156524	214214595	1	1	14989	-0.26858	0.02975	-2.71E-13	-0.26858
SET1	ENSG00000125629	2	118846028	118868573	4	1	14989	-0.13193	0.019305	-2.71E-13	-0.13193
SET1	ENSG00000241635	2	234526291	234681956	63	6	14989	-0.23499	0.0025108	-2.71E-13	-0.23499
SET1	ENSG00000242366	2	234668894	234681945	4	2	14989	0.39278	0.0016643	-2.71E-13	0.39278
SET1	ENSG00000114650	3	47455203	47518616	2	1	14989	0.96345	0.00084899	-2.71E-13	0.96345
SET1	ENSG00000084093	4	57774075	57802010	11	5	14989	-0.040513	0.00083476	-2.71E-13	-0.040513
SET1	ENSG00000112175	6	55618443	55740362	6	3	14989	0.81191	0.00017715	-2.71E-13	0.81191
SET1	ENSG00000204740	10	19492779	20079330	182	15	14989	-0.47973	0.0018625	-2.71E-13	-0.47973
SET1	ENSG00000107566	10	101909851	101948091	3	2	14989	0.27814	0.0020591	-2.71E-13	0.27814
SET1	ENSG00000050165	11	11984653	12031316	18	4	14989	0.71168	0.0001573	-2.71E-13	0.71168
SET1	ENSG00000012504	12	100867486	100958191	8	4	14989	0.053432	0.0010974	-2.71E-13	0.053432
SET1	ENSG00000130203	19	45409011	45412650	4	2	14989	3.7285	1.35E-12	-2.70E-13	3.7285
SET1	ENSG00000124216	20	48599536	48605423	14	1	14989	0.30703	0.0075763	-2.71E-13	0.30703
**#**	**_SET2_**	**VARIABLE**	**=**	**GO_bp:go_negative_regulation_of_alcohol_biosynthetic_process**	**(set)**						
**#**	**_SET2_**	**NGENES**	**=**	**7**							
**#**	**_SET2_**	**P-VALUE**	**=**	**4.32E-08**							
**_SET2_**	**GENE**	**CHR**	**START**	**STOP**	**NSNPS**	**NPARAM**	**N**	**ZSTAT**	**P**	**ZFITTED_BASE**	**ZRESID_BASE**
SET2	ENSG00000115956	2	68592305	68624585	1	1	14989	-0.40249	0.04001	-2.71E-13	-0.40249
SET2	ENSG00000114650	3	47455203	47518616	2	1	14989	0.96345	0.00084899	-2.71E-13	0.96345
SET2	ENSG00000084093	4	57774075	57802010	11	5	14989	-0.040513	0.00083476	-2.71E-13	-0.040513
SET2	ENSG00000112175	6	55618443	55740362	6	3	14989	0.81191	0.00017715	-2.71E-13	0.81191
SET2	ENSG00000107566	10	101909851	101948091	3	2	14989	0.27814	0.0020591	-2.71E-13	0.27814
SET2	ENSG00000050165	11	11984653	12031316	18	4	14989	0.71168	0.0001573	-2.71E-13	0.71168
SET2	ENSG00000130203	19	45409011	45412650	4	2	14989	3.7285	1.35E-12	-2.70E-13	3.7285
**#**	**_SET3_**	**VARIABLE**	**=**	**GO_bp:go_negative_regulation_of_cholesterol_biosynthetic_process**	**(set)**						
**#**	**_SET3_**	**NGENES**	**=**	**3**							
**#**	**_SET3_**	**P-VALUE**	**=**	**9.81E-09**							
**_SET3_**	**GENE**	**CHR**	**START**	**STOP**	**NSNPS**	**NPARAM**	**N**	**ZSTAT**	**P**	**ZFITTED_BASE**	**ZRESID_BASE**
SET3	ENSG00000114650	3	47455203	47518616	2	1	14989	0.96345	0.00084899	-2.71E-13	0.96345
SET3	ENSG00000107566	10	101909851	101948091	3	2	14989	0.27814	0.0020591	-2.71E-13	0.27814
SET3	ENSG00000130203	19	45409011	45412650	4	2	14989	3.7285	1.35E-12	-2.70E-13	3.7285
**#**	**_SET4_**	**VARIABLE**	**=**	**GO_bp:go_regulation_of_fertilization**	**(set)**						
**#**	**_SET4_**	**NGENES**	**=**	**17**							
**#**	**_SET4_**	**P-VALUE**	**=**	**8.29E-07**							
**_SET4_**	**GENE**	**CHR**	**START**	**STOP**	**NSNPS**	**NPARAM**	**N**	**ZSTAT**	**P**	**ZFITTED_BASE**	**ZRESID_BASE**
SET4	ENSG00000085465	1	111956936	111970399	11	1	14989	-0.043923	0.015283	-2.71E-13	-0.043923
SET4	ENSG00000116996	1	238045705	238054094	15	2	14989	0.27718	0.0021094	-2.71E-13	0.27718
SET4	ENSG00000163803	2	28680012	28866654	7	4	14989	0.56205	0.00021321	-2.71E-13	0.56205
SET4	ENSG00000186792	3	50330262	50336899	1	1	14989	-0.16442	0.02283	-2.71E-13	-0.16442
SET4	ENSG00000145685	5	77781038	78065844	64	8	14989	-0.44883	0.0022813	-2.71E-13	-0.44883
SET4	ENSG00000214510	5	147647743	147665817	5	1	14989	1.15	0.00039321	-2.71E-13	1.15
SET4	ENSG00000145888	5	151202074	151304403	4	2	14989	0.24065	0.0022238	-2.71E-13	0.24065
SET4	ENSG00000124812	6	49801970	49844809	2	1	14989	0.54761	0.0030459	-2.71E-13	0.54761
SET4	ENSG00000188372	7	76026835	76071388	7	2	14989	-0.35493	0.015378	-2.71E-13	-0.35493
SET4	ENSG00000146707	7	76239303	76256578	6	2	14989	0.21662	0.0033286	-2.71E-13	0.21662
SET4	ENSG00000105792	7	89874488	89940377	15	1	14989	0.17805	0.012121	-2.71E-13	0.17805
SET4	ENSG00000214102	7	141408153	141431071	2	1	14989	0.84388	0.0011865	-2.71E-13	0.84388
SET4	ENSG00000086062	9	33104080	33167354	25	3	14989	-0.091904	0.0047878	-2.71E-13	-0.091904
SET4	ENSG00000182545	14	20973696	20979328	2	1	14989	1.3759	0.00023614	-2.71E-13	1.3759
SET4	ENSG00000255346	15	69222864	69355083	42	1	14989	0.19256	0.0129	-2.71E-13	0.19256
SET4	ENSG00000196557	16	1203241	1271771	3	2	14989	0.41432	0.0014887	-2.71E-13	0.41432
SET4	ENSG00000182621	20	8112824	8949003	191	18	14989	0.21758	7.23E-05	-2.71E-13	0.21758
**#**	**_SET5_**	**VARIABLE**	**=**	**GO_bp:go_mrna_pseudouridine_synthesis**	**(set)**						
**#**	**_SET5_**	**NGENES**	**=**	**2**							
**#**	**_SET5_**	**P-VALUE**	**=**	**1.77E-06**							
**_SET5_**	**GENE**	**CHR**	**START**	**STOP**	**NSNPS**	**NPARAM**	**N**	**ZSTAT**	**P**	**ZFITTED_BASE**	**ZRESID_BASE**
SET5	ENSG00000091127	7	105080108	105162714	1	1	14989	2.1102	1.12E-05	-2.70E-13	2.1102
SET5	ENSG00000110060	11	125763381	125773116	2	1	14989	0.42915	0.0064858	-2.71E-13	0.42915
**#**	**_SET6_**	**VARIABLE**	**=**	**GO_cc:go_intermediate_density_lipoprotein_particle**	**(set)**						
**#**	**_SET6_**	**NGENES**	**=**	**2**							
**#**	**_SET6_**	**P-VALUE**	**=**	**9.55E-07**							
**_SET6_**	**GENE**	**CHR**	**START**	**STOP**	**NSNPS**	**NPARAM**	**N**	**ZSTAT**	**P**	**ZFITTED_BASE**	**ZRESID_BASE**
SET6	ENSG00000084674	2	21224301	21266945	2	1	14989	0.39716	0.0071139	-2.71E-13	0.39716
SET6	ENSG00000130203	19	45409011	45412650	4	2	14989	3.7285	1.35E-12	-2.70E-13	3.7285
**#**	**_SET7_**	**VARIABLE**	**=**	**GO_mf:go_phosphatidylcholine_sterol_o_acyltransferase_activator_activity**	**(set)**						
**#**	**_SET7_**	**NGENES**	**=**	**3**							
**#**	**_SET7_**	**P-VALUE**	**=**	**1.82E-06**							
**_SET7_**	**GENE**	**CHR**	**START**	**STOP**	**NSNPS**	**NPARAM**	**N**	**ZSTAT**	**P**	**ZFITTED_BASE**	**ZRESID_BASE**
SET7	ENSG00000110244	11	116691419	116694022	1	1	14989	0.17051	0.01031	-2.71E-13	0.17051
SET7	ENSG00000130203	19	45409011	45412650	4	2	14989	3.7285	1.35E-12	-2.70E-13	3.7285
SET7	ENSG00000130208	19	45417504	45422606	2	1	14989	2.3572	4.46E-06	-2.70E-13	2.3572

Table S5 – Functional Analysis

Table S6

QTL	chr	Gene Symbol	SNP Id	P-Value	Tissue
eQTL	17	*KANSL1-AS1*	rs199443	1.30E-43	Brain - Cortex
		RP11-259G18.3	rs199443	2.80E-39	Brain - Cortex
		LRRC37A2	rs199443	4.20E-37	Brain - Cortex
		MAPK8IP1P1	rs199443	7.00E-29	Brain - Cortex
		ARL17A	rs199443	2.70E-25	Brain - Cortex
		RP11-259G18.1	rs199443	1.80E-16	Brain - Cortex
		LRRC37A	rs199443	1.40E-12	Brain - Cortex
		CRHR1	rs199443	1.80E-04	Brain - Cortex
		KANSL1-AS1	rs199443	4.10E-37	Brain - Frontal Cortex (BA9)
		LRRC37A2	rs199443	6.90E-33	Brain - Frontal Cortex (BA9)
		RP11-259G18.3	rs199443	2.80E-31	Brain - Frontal Cortex (BA9)
		MAPK8IP1P1	rs199443	4.00E-25	Brain - Frontal Cortex (BA9)
		ARL17A	rs199443	9.10E-24	Brain - Frontal Cortex (BA9)
		RP11-259G18.1	rs199443	2.40E-12	Brain - Frontal Cortex (BA9)
		LRRC37A	rs199443	7.30E-11	Brain - Frontal Cortex (BA9)
		SPPL2C	rs199443	1.80E-05	Brain - Frontal Cortex (BA9)
	3	*RPSA*	rs13081054	1.30E-05	Brain - Cerebellar Hemisphere
		RPSA	rs13081054	2.40E-06	Brain - Cerebellum
sQTL	17	*KANSL1*	rs199443	3.10E-50	Brain - Cortex
		CRHR1	rs199443	2.90E-17	Brain - Cortex
		MAPT	rs199443	2.00E-06	Brain - Cortex
		KANSL1	rs199443	5.20E-43	Brain - Frontal Cortex (BA9)
		CRHR1	rs199443	2.10E-16	Brain - Frontal Cortex (BA9)
		MAPT	rs199443	5.80E-09	Brain - Frontal Cortex (BA9)
	3	*RPSA*	rs13081054	2.70E-06	Brain - Cortex

Table S6 - eQTL/sQTL from gTEX

Table S7

	H2 (SE)	Lambda_GC	Ratio (SE)	Intercept	H2 (se)	Lambda_GC
	**Observed scale**					
FTD	0.067 (0.03)	1.03	0.42(0.18)	1.01	0.118(0.02)	1.03
FTD_noAPOE	0.069(0.02)	1.03	0.4 (0.17)	1.01	0.117(0.02)	1.03
	**Liability scale**					
FTD	0.028(0.013)	1.03	0.42(0.18)	1.01	0.049 (0.0085)	1.03
FTD_noAPOE	0.029 (0.012)	1.03	0.4(0.17)	1.01	0.049 (0.008)	1.03

	r_g (SE)	P	N shared SNPs
FTD vs AD	0.55 (0.23)	0.02	1,166,449
FTD vs ADRD	0.28 (0.15)	0.06	1,080,004
FTD vs LBD	0.91 (0.46)	0.05	997,061
FTD vs ALS	0.71 (0.3)	0.02	1,160,125
FTD vs PD	0.32 (0.14)	0.03	1,091,414

Table S7 - Heritability estimation with LDSC regression

Table S8

Pheno	Marker	chr	Gene	Original work (p-value; OR)	Ref	Current study (Discovery) (p-value; OR)	LD with top SNP in current study	D'; R2
FTLD-TDP	rs1990622	7	*TMEM106B*	$1.08x10^{-11}$; 0.61	PMID: 20154673	$2.3x10^{-1}$; 0.96		
FTD-GRN	rs36196656	8	*GFRA2*	$1.58x10^{-8}$; 1.49	PMID: 29724592	$7.76x10^{-1}$; 1.01		
FTD-Clinical	rs9268877	6	*HLA-DR*	$1.05x10^{-8}$; 1.2	PMID: 24943344	$9.6x10^{-7}$; 1.16		
	rs302668	11	*RAB38/CTSC*	2.44×10^{-7}; 0.81		$9.86x10^{-2}$; 0.95		
ALS	rs3849943	9	*C9orf72*	3.8×10^{-30}; 0.84	PMID: 29566793	$5.5x10^{-1}$; 1.02		
	rs12973192*	19	*UNC13A*	3.9×10^{-15}; 0.89		$1.07x10^{-3}$; 1.1		
	rs142321490**	12	*KIF5A*	6.1×10^{-10}; 1.37		NA; NA		
	rs631312	3	*MOBP*	5.2×10^{-11}; 1.1	PMID: 34873335	$1.6x10^{-4}$; 1.13	Complete - non perfect	1; 0.3
AD	rs4420638	19	*APOC1*	$1.28x10^{-253}$; 3.95	PMID: 21460841	$9.6x10^{-8}$; 1.2	Almost complete - non perfect	0.77; 0.5
	rs439401		*APOE*	$1.06x10^{-49}$; 1.5		$8.1x10^{-1}$; 1.01	No LD	0.58; 0.047
	rs7412			$5.9x10^{-12}$; 1.5	PMID: 34099642	$1.9x10^{-4}$; 1.2	Complete - non perfect	1; 0.02
	rs6656401	1	*CR1*	5.7×10^{-24}; 1.18	PMID: 24162737	$5.4x10^{-2}$; 1.07		
	rs679515			7.2×10^{-46}; 1.13	PMID: 35379992	$1.1x10^{-1}$; 1.06		
	rs6733839	2	*BIN1*	6.9×10^{-44}; 1.22	PMID: 24162737	$2.96x10^{-2}$; 1.07		
	rs9331896	8	*CLU*	2.8×10^{-25}; 0.86		$9.3x10^{-1}$; 0.997		
	rs11787077			1.7×10^{-44}; 0.91	PMID: 35379992	$8.3x10^{-1}$, 0.99		
	rs3851179	11	*PICALM*	3×10^{-48}; 0.9		$6.5x10^{-3}$, 0.92		
	rs6605556***	6	*HLA-DR*	7.1×10^{-20}; 0.91		$2.8x10^{-2}$, 0.91		
	rs199515****	17	*MAPT*	9.3×10^{-13}; 1.06		$1.1x10^{-9}$, 1.3	Complete - perfect	1; 0.97
	rs1009966$	3	*MOBP*	$3.4x10^{-1}$; 1.01	PMID: 31417202	(joint analysis p-value current manuscript)		
				$7.5x10^{-1}$; 1	PMID: 35379992	$2.41x10^{-8}$, 1.16		
PD	rs17649553	17	*MAPT*	1.26×10^{-68}; 0.78	PMID: 28892059	$1.97x10^{-8}$; 0.82	Complete - perfect	1; 0.91
PSP	rs1411478	1	*STX6*	2.3×10^{-10}; 0.79	PMID: 21685912	$7.82x10^{-3}$; 1.08		
	rs7571971	2	*EIF2AK3*	3.2×10^{-13}; 0.75		$6.6x10^{-1}$; 1.01		
	rs1768208	3	*MOBP*	1.0×10^{-16}; 0.72		$5.13x10^{-4}$; 1.12	Complete - non perfect	1; 0.31
	rs8070723	17	*MAPT*	1.5×10^{-116}; 5.46		$9.21x10^{-9}$; 0.81	Complete - perfect	1; 0.94
	rs11568563	12	*SLCO1A2*	5.26E-10; 0.67	PMID: 29986742	$4.66x10^{-3}$; 1.19		
	rs6687758	1	*DUSP10*	1.14×10^{-8}; 0.80		$7.3x10^{-1}$; 1.01		
CBD	rs393152	17	*MAPT*	1.42×10^{-12}; 3.70	PMID: 26077951	$2.06x10^{-8}$; 0.82	Complete - perfect	1; 0.94
	rs643472	8	*lnc-KIF13B-1*	3.41×10^{-8}; 1.82		$8.5x10^{-1}$; 0.993		

* = PROXY rs12608932 (D'=1; R2=0.98)
** = PROXY not available
*** = PROXY rs9272461 (D'=1; R2=1)
**** = PROXY rs199528 (D'=1; R2=1)
$ = *MOBP* hit reported in the current manuscript checked in 2 AD-GWAS datasets

Table S8 - Assessment of significant markers from other neurodegenerative disease or studies vs. the current study

Note S1

Characterisation of *APOE Ɛ4* in the cohort

We first analysed the *APOE Ɛ4* alleles by extracting the allele frequencies for rs429358 (C:T) and rs7412 (T:C) in the original discovery and replication cohorts, and compared cases vs. controls (**Table**): allele frequency differences were statistically significant in both the discovery and replication cohorts ($p_{discovery}$=3.6x10^{-14}; $p_{replication}$=5.5x10^{-4}) suggesting that the cases in our cohorts are enriched in Ɛ4 alleles.

Population	CASES count	CASES freq	CONTROLS count	CONTROLS freq
**Discovery**	1179	0.157	2759	0.123
**Replication**	301	0.162	1068	0.131
**1000 Genomes EUR**			114	0.141
**Discovery Mediterranean**	417	0.149	500	0.087
**Replication Mediterranean**	159	0.152	130	0.096
**1000 Genomes IBR+TSI**			49	0.114
**New cohort Italians**			135	0.093
**Discovery Central/Nordic**	762	0.162	2259	0.135
**Replication Central/Nordic**	142	0.175	938	0.138
**1000 Genomes CEU+GBR**			65	0.171
**New cohort Europeans**			100	0.145

We then did the same for the two sub-cohorts (Central/Nordic cases vs. controls and Mediterranean cases vs. controls): all comparisons indicated that allele frequency differences were statistically significant ($p_{Central/Nordic}$ =3.7x10^{-6}, $p_{Mediterranean}$=2.2x10^{-16}), confirming a higher frequency of Ɛ4 alleles in FTD cases regardless from ancestry.

Finally, we compared the *APOE Ɛ*4 allele frequencies between our Central/Nordic and Mediterranean European controls: allele frequency differences were statistically significant ($p_{Discovery-Controls}$=2.2x10^{-16}), indicating that Central/Nordic European controls carried a remarkably higher frequency of Ɛ4 alleles compared to Mediterranean European controls. We confirmed this trend in further independent cohorts, *i.e.* Central/Nordic vs. Mediterranean Europeans for (1) the populations from 1000G (p_{1000G}=2.7x10^{-2}) and (2) an additional cohort of 731 Italian and 347 European controls ($p_{NewCohort}$=3.4x10^{-4}).

All this taken together suggests that: (1) FTD cases carry a slightly increased frequency of Ɛ4 alleles compared to controls, regardless of ancestry and (2) Central/Nordic Europeans (control population) carry a higher frequency of Ɛ4 alleles compared to Mediterranean Europeans (control population) regardless of disease status (**Figure**).

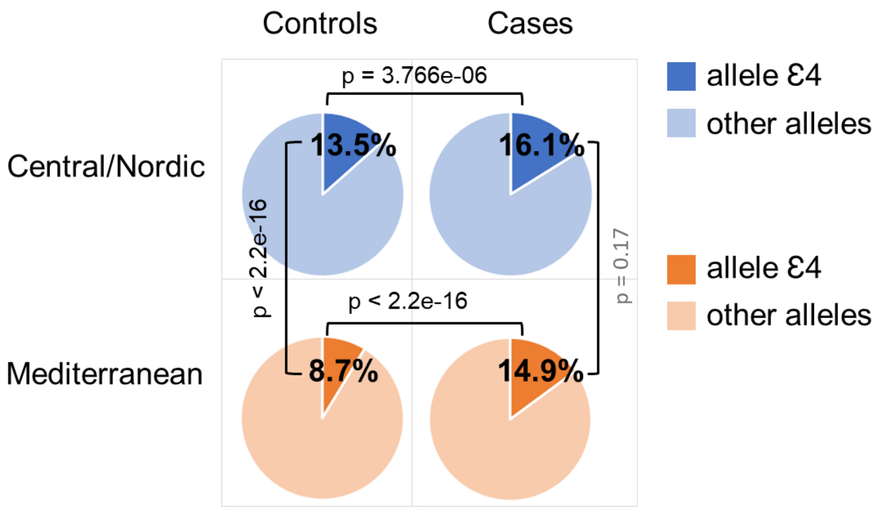

Figure– *APOE Ɛ4 allele frequencies in the Central/Nordic and Mediterranean discovery cohorts. Uncorrected p-values calculated via* χ^2 *are reported.*

Note S2 – list of PIs

Pis Name
Diego Albani
Giuliano Binetti and/or Roberta Ghidoni
Daniel Blackburn
Barbara Borroni
Carlos Cruchaga
Adrian Danek
Janine Diehl-Schmid
Carol Dobson-Stone
Oriol Dols-Icardo
Daniela Galimberti
Maurizio Gallucci
Caroline Graff
Jordan Grafman
Edward Huey
Isabelle Le Ber
Ian Mackenzie
Raffaele Maletta
Manuel Menendez-Gonzalez
Christopher Morris
Benedetta Nacmias
Jørgen Nielsen
Valeria Novelli
Suvankar Pal
Florence Pasquier
Pau Pastor
Robert Perneczky
Yolande Pijnenburg
Rosa Rademakers
Innocenzo Rainero
Matthias Riemenschneider
Ekaterina Rogaeva
Boris Rogelj
Giacomina Rossi
James Rowe
Agustín Ruiz
Raquel Sanchez-Valle
Sigrid Sando
Jennifer Saxon and/or Jennifer Thompson
Johannes Schlachetzki
Vivianna Van Deerlin
Julie van der Zee
John Van Swieten
Emilia Vitale
Maria Landqvist-Waldo
Jennifer Yokoyama

Additional acknowledgement and funding

JH – This work was supported by the UK Dementia Research Institute which receives its funding from UK DRI Ltd (UKDRI-1009) funded by the UK Medical Research Council, Alzheimer's Society and Alzheimer's Research UK. JH received funding from the Dolby Family Fund; VA – is supported by the Fondo de Investigaciones Sanitarias' Spanish government ICIII FIS-FEDER grants (ID grant: PI21/0467); OAA – Research Council of Norway (223273), Norwegian Health Association (#22731); RG & LB & GB – Italian Ministry of Health, Ricerca Corrente; DJB – Sheffield BRC; MB & MM & AR – The Genome Research @ Ace Alzheimer Center Barcelona project (GR@ACE) is supported by Grifols SA, Fundación bancaria 'La Caixa', Ace Alzheimer Center Barcelona and CIBERNED. Ace Alzheimer Center Barcelona is one of the participating centers of the Dementia Genetics Spanish Consortium (DEGESCO). Acknowledge the support of the Spanish Ministry of Science and Innovation, Proyectos de Generación de Conocimiento grants PID2021-122473OA-I00, PID2021-123462OB-I00 and PID2019-106625RB-I00. ISCIII, Acción Estratégica en Salud, integrated in the Spanish National R+D+I Plan and financed by ISCIII Subdirección General de Evaluación and the Fondo Europeo de Desarrollo Regional (FEDER "Una manera de hacer Europa") grants PI13/02434, PI16/01861, PI17/01474, PI19/00335, PI19/01240, PI19/01301, PI22/01403, PI22/00258 and the ISCIII national grant PMP22/00022, funded by the European Union (NextGenerationEU). The support of CIBERNED (ISCIII) under the grants CB06/05/2004 and CB18/05/00010. The support from the ADAPTED and MOPEAD projects, European Union/EFPIA Innovative Medicines Initiative Joint (grant numbers 115975 and 115985, respectively); from PREADAPT project, Joint Program for Neurodegenerative Diseases (JPND) grant Nº AC19/00097; from HARPONE project, Agency for Innovation and Entrepreneurship (VLAIO) grant Nº PR067/21 and Janssen. DESCARTES project is funded by German Research Foundation (DFG); BFB – P50 AG016574 and P30 AG062677; SB-E – recipient of the Research Contract FBBVA-Rodés-Baselga; BC – Alzheimer's Society for its support and funding (grant 447); CC – was supported by grants from the National Institutes of Health, R01AG044546 (CC), P01AG003991(CC, JCM), RF1AG053303 (CC), RF1AG058501 (CC), U01AG058922 (CC), and the Chuck Zuckerberg Initiative (CZI), and the Alzheimer's Association Zenith Fellows Award (ZEN-22-848604, awarded to CC). This work was supported by access to equipment made possible by the Hope Center for Neurological Disorders, the Neurogenomics and Informatics Center (NGI: https://neurogenomics.wustl.edu/)and the Departments of Neurology and Psychiatry at Washington University School of Medicine; DWD – P50NS072187, P50AG016574, State of Florida Alzheimer Disease Initiative, and CurePSP Inc; CD-S – is supported by National Health and Medical Research Council of Australia (NHMRC) Boosting Dementia Research Leadership Fellowship 1138223 and the University of Sydney; OD-I – is a recipient of a grant by The Association for Frontotemporal Degeneration (Clinical Research Postdoctoral Fellowship); CG – is supported by grants from Swedish FTD Initiative-The Schörling Foundation, JPND Prefrontals Research Council (VR) 529-2014-7504, Research Council (VR) 2015-02926, Research Council (VR) 2018-02754, Brain Foundation, Alzheimer Foundation, Region Stockholm (ALF-Project) and Demensfonden, Sweden; NRG-R – P50 AG016574 and P30 AG062677; KAJ – R01 AG037491; DSK – P50 AG016574 and P30 AG062677; MK – UCL Genomics core facility; ILB – French clinical and genetic research network on FTD/FTD-ALS; GL – supported by UK DRI; IRM – Research funding from CIHR and NIH; GKM – UCL Genomics core facility; MM-G – is supported by the Fondo de Investigaciones Sanitarias' Spanish government ICIII FIS-FEDER grants (ID grants: PI21/0467); BLM – P30AG062422, P01AG019724; CMM – Newcastle Brain Tissue Resource, which is funded in part by a grant from the UK Medical Research Council, by NIHR Biomedical Research Centre Newcastle awarded to the Newcastle upon Tyne NHS Foundation Trust and Newcastle University, and as part of the Brains for Dementia Research Programme jointly funded by Alzheimer's Research UK and Alzheimer's Society; HRM – PSP Association; BN – RICATENEO2023; MAN – supported in part by the Intramural Research Program of the NIH, National Institute on Aging (NIA), National Institutes of Health, Department of Health and Human Services; project number ZIAAG000534, as

well as the National Institute of Neurological Disorders and Stroke. This work utilized the computational resources of the NIH HPC Biowulf cluster (http://hpc.nih.gov); RP – is supported by Davos Alzheimer's Collaborative, the VERUM Foundation, the Robert-Vogel-Foundation, the German Center for Neurodegenerative Diseases (DZNE), the National Institute for Health and Care Research (NIHR) Sheffield Biomedical Research Centre (NIHR203321), the University of Cambridge – Ludwig-Maximilians-University Munich Strategic Partnership within the framework of the German Excellence Initiative and Excellence Strategy and the European Commission under the Innovative Health Initiative program (project 101132356); RCP – P50 AG016574 and P30 AG062677; OP – is supported by a National Health and Medical Research Council of Australia Leadership Fellowship (GNT2008020); RR – P50 AG016574, R01 NS080882, R01 NS065782, P50 NS72187, and the Consortium for Frontotemporal Dementia; IR and ER – Grant of the Italian Ministry of the University (MUR), project "Dipartimenti di Eccellenza 2023–2027" to the Department of Neuroscience "Rita Levi Montalcini", University of Torino, Italy. ER – the Canadian Consortium on Neurodegeneration in Aging; HR – AG032306 and K24 AG045333; JBR – was supported by the NIHR Cambridge Biomedical Research Centre (NIHR203312). The views expressed are those of the author(s) and not necessarily those of the NIHR or the Department of Health and Social Care' Wellcome Trust (220258; 103838). For the purpose of open access, the authors have applied a CC BY public copyright licence to any Author Accepted Manuscript version arising from this submission; NIHR Cambridge Biomedical Research Centre (NIHR203312); the views expressed are those of the author and not necessarily those of the NIHR or the Department of Health and Social Care. Cambridge Centre for Parkinson-plus; SWS – was supported in part by the intramural research program of the National Institute of Neurological Disorders and Stroke, National Institutes of Health (program #: ZIANS003154); WWS – NIH AG023501, AG019724, Consortium for Frontotemporal Dementia Research; MS – is supported by the Italian Ministry of Health, grant GR-2019-12369100; ABS – was supported in part by the Intramural Research Program of the NIH, National Institute on Aging (NIA), National Institutes of Health, Department of Health and Human Services; project number ZIAAG000534, as well as the National Institute of Neurological Disorders and Stroke. This work utilized the computational resources of the NIH HPC Biowulf cluster (http://hpc.nih.gov); SS – RICATENEO2023; PStG-H – the Canadian Consortium on Neurodegeneration in Aging; MMXT – is employed by Oslo University Hospital. She has received grant support from Parkinson's UK, the Michael J Fox Foundation, and South-Eastern Norway Regional Health Authority (Helse Sør-Øst); CVB – was in part funded by the MetLife Foundation Award for Medical Research, the Methusalem Excellence Program of the Flemish Government and the University of Antwerp Research Fund, Belgium; VMVD – P01 AG-066597, P30-AG-072979; JvdZ – was in part funded by the MetLife Foundation Award for Medical Research, the Methusalem Excellence Program of the Flemish Government and the University of Antwerp Research Fund, Belgium; KJ – NIA grant R01-AG037491; SJVdL – The Alzheimer Center Amsterdam is supported by Stichting Alzheimer Nederland and Stichting VUmc fonds. The clinical database structure was developed with funding from Stichting Dioraphte. Genotyping of the Dutch case-control samples was performed in the context of EADB (European Alzheimer DNA biobank) funded by the JPco-fuND FP-829-029 (ZonMW projectnumber 733051061); is recipient of funding from ZonMW (#733050512), Health~Holland, Topsector Life Sciences & Health (PPP-allowance; #LSHM20106). Stichting Dioraphte, the Edwin Bouw Fonds and Stichting VUmc fonds. SvdL is recipients of ABOARD, which is a public-private partnership receiving funding from ZonMW (#73305095007) and Health~Holland, Topsector Life Sciences & Health (PPP-allowance; #LSHM20106). The Alzheimer Center Amsterdam is supported by Stichting Alzheimer Nederland and Stichting VUmc fonds. The clinical database structure was developed with funding from Stichting Dioraphte. Genotyping of the Dutch case-control samples was performed in the context of EADB (European Alzheimer DNA biobank) funded by the JPco-fuND FP-829-029 (ZonMW projectnumber 733051061); S.L. is recipient of funding from ZonMW (#733050512), Health~Holland, Topsector Life Sciences & Health (PPP-allowance; #LSHM20106). Stichting Dioraphte, the Edwin Bouw Fonds and Stichting VUmc

fonds; WF, SvdL, HHolstege, CT and PhS are recipients of ABOARD, which is a public-private partnership receiving funding from ZonMW (#73305095007) and Health~Holland, Topsector Life Sciences & Health (PPP-allowance; #LSHM20106). More than 30 partners participate in ABOARD (www.aboard-project.nl). ABOARD also receives funding from de Hersenstichting, Edwin Bouw Fonds and Gieskes-Strijbisfonds; LMR – was funded by the NIH National Institute on Aging (NIA) R21 AG072390 and by the Memorabel fellowship (ZonMW projectnumber: 10510022110012); YALP – is a recipient of funding from NWO (KICH1.GZ02.20.004), Stichting ZABAWAS, Hersenstichting and Health~Holland. IdR is supported by a national grant from the Instituto de Salud Carlos III (ISCIII) FI20/00215.
